# Elaborate the Mechanism of Ancient Classic Prescriptions (Erzhi Formula) in Reversing GIOP by Network Pharmacology Coupled with Zebrafish Verification

**DOI:** 10.1155/2022/7019792

**Published:** 2022-01-10

**Authors:** Zhihui Cai, Huajun Wang, Jun Jiang, Shichang Xiao, Jianpeng Xiao, Jinjin He, Zihan Zhao, Jiangning Yin

**Affiliations:** ^1^School of Pharmacy, Jiangsu University, No. 301 Xuefu Road, Zhenjiang, Jiangsu 212013, China; ^2^Affiliated Hospital of Jiangsu University, No. 438 Jiefang Road, Zhenjiang, Jiangsu 212001, China; ^3^The Affiliated Jiangning Hospital of Nanjing Medical University, Nanjing, Jiangsu 211100, China

## Abstract

Osteoporosis is a degenerative disease that endangers human health. At present, chemical drugs used for osteoporosis have serious side effects. Therefore, it is valuable to search herbs with high safety and good curative effect in antiosteoporosis. Erzhi formula (EZF), an ancient classic compound, has been reported to have a beneficial effect in antiosteoporosis, but its mechanism is unclear. In this paper, the active compounds of EZF were found in Systems Pharmacology Database, and gene targets related to osteoporosis were obtained in GeneCards. The GO functional and KEGG pathway enrichment analysis were performed by Metascape. The network of “components-targets-signal pathway” was constructed by Cytoscape. Next, molecular docking between the active components and hub genes related to the PI3K-Akt signaling pathway was conducted by Autodock. In the verification experiment, the zebrafish induced by prednisolone (PNSL) was used to reproduce glucocorticoid-induced osteoporosis (GIOP) model, and then the reversal effects of EZF were systematically evaluated according to the behavior, skull staining area, bone mineralization area (BMA), average optical density (AOD), and cumulative optical density (COD). Finally, it was shown that 24 components in EZF could regulate 39 common gene targets to exert antiosteoporosis effect. Besides, the main regulatory mechanisms of EZF were 4 signaling pathways: PI3K-Akt, JAK-STAT, AGE-RAGE, and cancer pathway. In PI3K-Akt signaling pathway, wedelolactone, dimethyl wedelolactone, specnuezhenide, ursolic acid, acacetin, beta-sitosterol, apigenin, and kaempferol can bind tightly with EGF, IL-2, and IL-4 genes. Compared with the model group, the moving distance, swimming speed, and cumulative swimming time of zebrafish in EZF group were significantly increased (*P* < 0.05). Meanwhile, the BMA and COD of zebrafish were significantly improved after the intervention of EZF (*P* < 0.05). In summary, the 24 components of EZF exert their antiosteoporosis effects by regulating 39 related gene targets, among which the PI3K signaling pathway is crucial. EZF can promote bone formation and reversed GIOP through “multicomponent/multitarget/multipathway” and the medium dose of EZF may be the most suitable concentration for the treatment of GIOP in zebrafish model.

## 1. Introduction

Osteoporosis is a silent disorder characterized by reduced bone density and structural deterioration [1], which is caused by the change of bone microstructure and makes patients vulnerable to have brittle fractures. Osteoporosis leads to a remarkable decrease of life quality and increases the mortality and disability rate at the same time. According to the World Health Organization (WHO), more than 200 million people are affected worldwide, and it is more prevalent in postmenopausal women, with about 25 to 30 percent prevalence in the United States and Europe [2]. It is a chronic condition that affects one in three women and one in five men over the age of 50 [3]. Glucocorticoids (GCs) are widely used in chronic noninfectious inflammatory diseases, allergic diseases, and organ transplantation. Even physiological doses of GCs can cause bone loss, so osteoporosis is one of the most serious side effects of GCs. Generally, this kind of osteoporosis caused by long-term use of GCs is called glucocorticoid-induced osteoporosis (GIOP) [4, 5], which may lead to up to 20% of osteoporosis cases [6]. Generally, it develops in a time-dose-dependent manner, and an increased risk of brittle fractures may be observed within the first month of treatment even at low (<2.5 mg prednisone equivalent) GC daily dose [7]. Published guidelines for the treatment of patients with chronic GCs recommend calcium and vitamin D supplementation, bone mineral density testing, and bisphosphonate therapy [8–10]. However, according to the guidelines for treating GIOP, even if it is treated with the recommended drugs, patients may face the threat of systemic calcium loss, erosive esophagitis, ulcer bleeding, hypocalcemia, decline of renal function, jaw necrosis, or atypical femoral fracture caused by reduced gastrointestinal absorption and renal tubular reabsorption [11–13]. In conclusion, it is urgent to find a relatively safe and effective drug for the treatment of GIOP.

As a well-known formula in China, EZF is mixed of *Fructus Ligustri Lucidi* (FLL) and *Ecliptae herba* (EP) with a ratio of 1 : 1. It has been commonly used for treating menopausal diseases. FLL was first recorded in “The Medical Focus Explanation” in the Qing Dynasty [14]. FLL is the fruit of *Ligustrum lucidum* Ait., which is commonly used to nourish liver and kidney system, improve eyesight, and strengthen muscles and bones in Chinese medicine. EP, the dry aerial part of *Eclipta prostrata* L., has been widely used since ancient times to nourish the liver and kidney, strengthen teeth, darken hair and beard [15, 16]. There have been many studies on EZF in the remedy of GIOP. For example, Yang et al. reported that the combination of EZF and Epimedium can significantly prevent glucocorticoid-induced bone loss, increase the content of BMP-2 which is the bone formation marker, reduce serum TRACP, and inhibit bone resorption [12, 13]. What is more, EZF, as an ancient classic prescription for chronic diseases like GIOP, has been playing an undeniable role since ancient times. Therefore, the advantages of EZF in the treatment of GIOP are not only reflected in its safety and effectiveness but also reflected in the excavation and inheritance of effective classic prescriptions. However, the relationship among its pharmacological effects, therapeutic targets, and signaling pathways for GIOP remains unclear, which limits the wide use of EZF in clinical practice.

Chinese materia medica is a complex system of multicomponent, multitarget, and synergistic action among components. Clearly explaining the relationship among these components, targets, and pathways is a hot and difficult problem all the time. Fortunately, network pharmacology, which has been continuously improved and developed since Li's first proposal [17, 18], is a new discipline that systematically reveals the action of Chinese medicine on human body and predicts the potential mechanism by constructing the complex network of “drug-active ingredients-gene targets-disease” [19]. The greatest advantage of the development and application of this technology is that the “network” combines the evaluation of network topology and dynamics, so as to provide visual analysis of complex drug components of EZF [20]. In conclusion, network pharmacology can be applied to the research on the potential mechanism of EZF for GIOP, which provides a new perspective and strategy for the research of ancient classic prescriptions. Besides, zebrafish is a full sequence model organism with highly conserved innate immune system, including cell types and signaling molecules [21]. The advantages of zebrafish such as optical clarity, development speed, and fertility make it a popular vertebrate model for developmental biology research and animal model for studying disease processes [22]; in particular, its bone morphology can be observed directly by staining. In this paper, a network-pharmacology approach was followed to explore the antiosteoporotic mechanism of EZF, and then the medicinal efficacy was confirmed by zebrafish model.

## 2. Materials and Methods

### 2.1. Screening Active Components and Corresponding Targets

Active ingredients of EZF were searched based on Traditional Chinese Medicine Systems Pharmacology Database and Analysis Platform (TCMSP, http://tcmspw.com/tcmsp.php, PubChem, https://pubchem.ncbi.nlm.nih.gov, and SwissTargetPrediction database, http://www.swisstargetprediction.ch). Notably, the screening conditions in TCMSP were oral bioavailability (OB) ≥ 30% and drug-likeness (DL) ≥ 0.18 [23]. Immediately, the corresponding drug targets were searched according to the captured active ingredients. Finally, the target proteins reviewed in human were transformed into gene symbols by UniProt database (https://www.uniprot.org/) [24].

### 2.2. Collection of Osteoporosis-Related Targets

The collection of disease genes depends on the utilization of GeneCards database (https://www.genecards.org/) [25]. “Osteoporosis” and “glucocorticoid osteoporosis” were set as key words in the database and their related targets were obtained.

### 2.3. Screening the Common Targets between Drug and Disease

The function of COUNTIF was used to capture the common targets between genes related to active components in EZF and the disease target genes of osteoporosis. Then, we imported the common targets into the Bioinformatics platform (bioinformatics.com.cn) [26]. Lastly, a Venn diagram of the EZF-GIOP-related targets was obtained successfully.

### 2.4. Protein-Protein Interaction (PPI) Network Construction and Hub Gene Screening

PPI is composed of proteins through their interactions to participate in various links of life processes such as biological signal transmission, gene expression regulation, and energy and material metabolism [27]. The STRING database (https://string-db.org) was used to perform a PPI network analysis based on the common targets obtained [28]. The species was limited to “*Homo sapiens*” with parameter of moderate confidence greater than 0.4. Then we obtained a network of PPI. Next, TSV files were imported into CytoHubba, which is the plug-in unit in Cytoscape (3.6.0). One of the CytoHubba algorithms (Degree, Maximal Clique Centrality (MCC), Clustering Coefficient, Density of Maximum Neighborhood Component (DMNC), BottleNeck, Maximum Neighborhood Component (MNC), Radiality, Edge Percolated Component (EPC), Eccentricity, Closeness, Betweenness, and Stress) was used to find the top 10 hub genes.

### 2.5. Enrichment Analysis

The enrichment analysis mainly included two parts: Gene Ontology (GO) functional enrichment and the Kyoto Encyclopedia of Genes and Genomes (KEGG) pathway enrichment. Before using Metascape platform (http://metascape.org/gp/index.html#/main/step1) for analysis [29], it is also necessary to import common targets into the “Multiple Gene List” and then click “Custom Analysis.” GO functional enrichment analysis contains biological process (BP), cellular component (CC), and molecular function (MF) [30]. Moreover, the top 16 signaling pathways are significantly related to drug disease selected from the results of KEGG enrichment analysis [31].

### 2.6. Construction of the Network of “Active Components-Gene Targets-Disease”

Two Excel files named “Network” and “Type” should be established, respectively, before analysis. Next, the two files were imported into the Cytoscape 3.6. 0 software successively [32]. Finally, the “active components-gene targets-disease” network [33] was successfully constructed by Cytoscape.

### 2.7. Molecular Docking Verification

On the one hand, we obtained the active components' structure from PunChem website (https://pubchem.ncbi.nlm.nih.gov) and saved it in SDF form. Then we used ChemBio3D software to minimize the binding energy and converted it into 3D structure. On the other side, the protein structures of hub genes were found from PDB database (https://www.rcsb.org/search) and saved as PDB structure. Hydrogenation of proteins and small molecular ligands was performed with PyMOL. Finally, Autodock Vina was used for molecular docking and calculating the binding energy [34].

### 2.8. Experimental Animals

The wild type AB strain zebrafish embryo with 3 days post fertilization (3 DPF) was supplied by Yi Shu Li Hua company (Nanjing, China) and maintained according to standard conditions (14 : 10 h light/dark cycle at 28°C) [35]. Animal experiments were conducted in accordance with the Guidelines for Animal Experiments of Jiangsu University and approved by the Animal Ethics Committee.

### 2.9. Preparation of EZF

Firstly, the EP and FLL were crushed, respectively, and passed through a 60-mesh sieve, and then they were mixed according to the ratio of 1 : 1 to prepare EZF. The mixture was refluxed with 10 times ethanol (50%) twice for 1 hour each time, and the two filtrates were combined. After refluxing, the collected extracts were evaporated in a rotary evaporator until there was no alcohol. Lastly, samples were lyophilized in a freeze dryer.

### 2.10. Animal Grouping and Intervention

Zebrafish embryos with normal morphology were placed in incubator and cultured in 6-well plate containing E_3_ culture medium. The culture environment was maintained at 14 h/10 h light/dark cycle, and the room temperature was controlled at 28.5 ± 0.5°C. After 3 days post fertilization (3 DPF), the zebrafish larvae were divided into seven groups: blank control group (*E*_3_), blank DMSO group (*E*_3_ + 0.5% DMSO), model group (*E*_3_ + 0.5% DMSO + 25 *μ*M PNSL), EZF low-dose group (*E*_3_ + 0.5% DMSO + 25 *μ*M PNSL+ 0.1 *μ*g/mL EZF), EZF medium-dose group (*E*_3_ + 0.5% DMSO + 25 *μ*M PNSL + 1.0 *μ*g/mL EZF), EZF high-dose group (*E*_3_ + 0.5% DMSO + 25 *μ*M PNSL + 10.0 *μ*g/mL EZF), and positive control group (PC, *E*_3_ + 0.5% DMSO + 25 *μ*M PNSL+ 15 *μ*M etidronate disodium) randomly (*n* = 15). The specific culture and administration methods were shown in the previous published article [36].

### 2.11. Observation of Behavior and Bone Mineralization

The groups of zebrafish mentioned above were placed in 6-well plates with 6 fish per hole, and then the data of swimming behavior of zebrafish were recorded and analyzed by animal behavior analyzer. After 10 DPF, zebrafish were anesthetized with MS-222 solution, fixed with 4% paraformaldehyde solution, and dyed with Alizarin Red S. The final step of pretreatment was bleaching and decolorizing [37]. Lastly, the bone staining of zebrafish was observed under the microscope. All zebrafish samples were stored in glycerin solution. After obtaining the image of the ventral side of the skull by microscope, BMA, AOD, and COD of zebrafish skull were quantitatively analyzed by ImageJ software.

### 2.12. Statistical Analysis

All data were presented as the mean ± SD and statistically analyzed with statistical software SPSS 19.0 (SPSS, Inc., Chicago, IL). One-way analysis of variance was used for difference analysis, and *P* < 0.05 was considered as significant difference. [38].

## 3. Results

### 3.1. Common Targets between “Active Ingredients” and “Osteoporosis”

**Figure 1:**
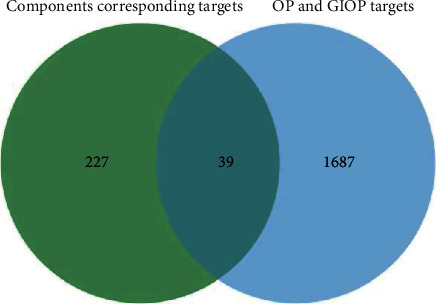
Venn diagram of drug targets and disease common targets.

53 active compounds of EZF were obtained in TCMSP, and their 266 corresponding gene symbols were found. Furthermore, 1276 gene symbols related to the prevention of osteoporosis were acquired from GeneCards database. Venn analysis was used to obtain the common gene targets of “active ingredients” and “glucocorticoid osteoporosis,” as shown in Figure 1. Finally, the 24 screened out chemical components corresponding to 39 gene targets of EZF are given in Table 1. The results showed that EZF could regulate 39 of the related targets to intervene in osteoporosis.

### 3.2. PPI Network and Hub Gene

**Figure 2:**
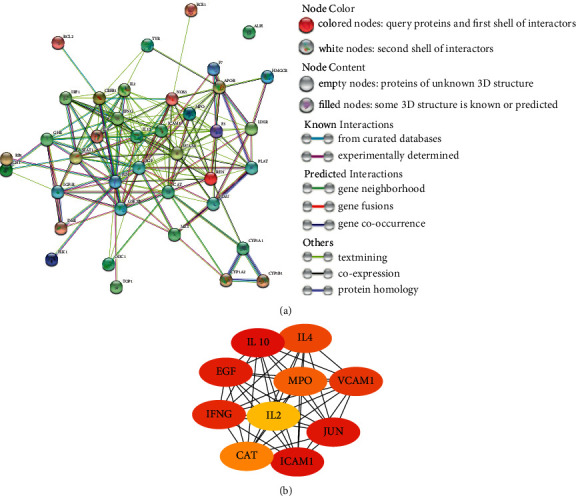
Protein-protein interaction (PPI) network and top 10 hub genes. (a) Protein-protein interaction (PPI) network; (b) top 10 hub genes selected by MCC method.

39 common targets were imported into the STRING website to obtain the interaction relationships with each other. As shown in Figure 2(a), we can get a PPI network diagram by setting parameters. The nodes represent proteins and edges represent protein-protein associations, from which we can learn that there are 39 nodes and 187 edges and the average node degree is 9.59. Using CytoHubba plug-in of Cytoscape, the top 10 core target genes were selected through MCC algorithm calculation (Figure 2(b) and Table 2).

### 3.3. GO and KEGG Pathway Enrichment Analysis

**Figure 3:**
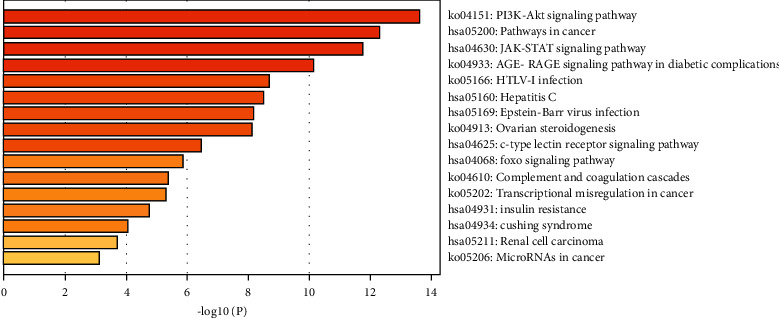
KEGG enrichment analysis for signal pathways. The *y*-axis shows top significantly enriched KEGG categories, and the *x*-axis displays the number of enrichment genes of these terms (*P* < 0.05), and the color represents the adjusted *P* value; the redder, the more significant the enrichment. The height of the column is related to *P* value. The higher the column is, the more significant the enrichment is.

39 EZF-GIOP related targets were imported into metascape, and then the results of KEGG pathway analysis and GO-MF, GO-BP, and GO-CC analysis were obtained one by one. According to the results, EZF could regulate GIOP through several signaling pathways, such as PI3K-Akt, cancer, JAK-STAT, AGE-RAGE, HTLV-I infection, Hepatitis C, Epstein-Barr virus infection, ovarian steroidogenesis, C-type lectin receptor, FOXO, complement and coagulation cascades, transcriptional misregulation in cancer, insulin resistance, cushing syndrome, renal cell carcinoma, and microRNAs in cancer. In addition, the main signaling pathways were PI3K-Akt and pathways in cancer, JAK-STAT, and AGE-RAGE (Figure 3).

**Figure 4:**
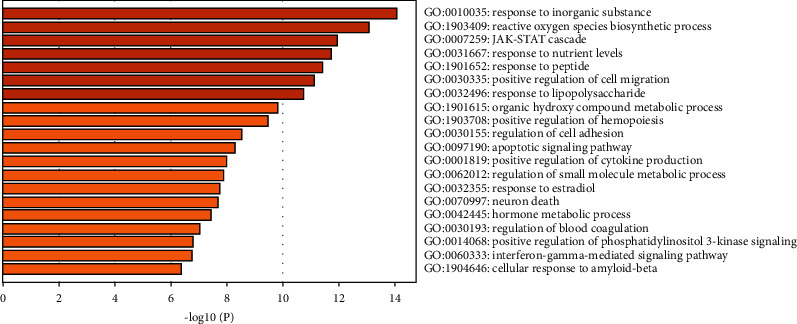
GO-BP biological process of enrichment analysis. The *y*-axis shows top significantly enriched GO-BP categories, and the *x*-axis displays the number of enrichment genes of these terms (*P* < 0.05), and the color represents the adjusted *P* value; the redder, the more significant the enrichment. The height of the column is related to *P* value. The higher the column is, the more significant the enrichment is.

**Figure 5:**
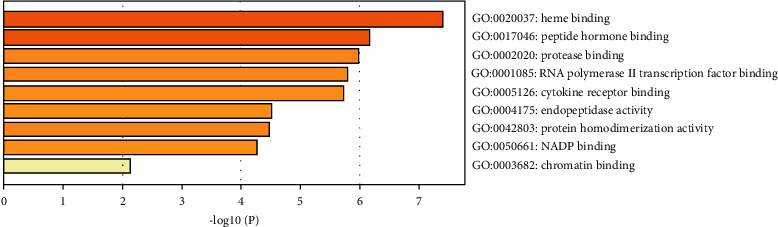
GO-MF molecular function of enrichment analysis. The *y*-axis shows top significantly enriched GO-MF categories, and the *x*-axis displays the number of enrichment genes of these terms (*P* < 0.05), and the color represents the adjusted *P* value; the redder, the more significant the enrichment. The height of the column is related to *P* value. The higher the column is, the more significant the enrichment is.

**Figure 6:**
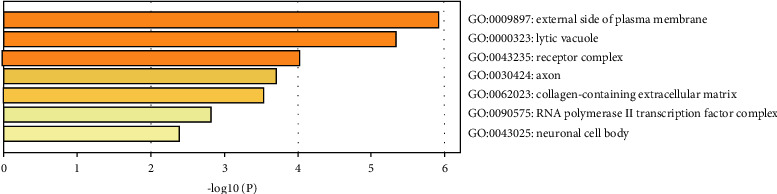
GO-CC cellular component of enrichment analysis. The *y*-axis shows top significantly enriched GO-CC categories, and the *x*-axis displays the number of enrichment genes of these terms (*P* < 0.05), and the color represents the adjusted *P* value; the redder, the more significant the enrichment. The height of the column is related to *P* value. The higher the column is, the more significant the enrichment is.

Interestingly, GO-BP analysis showed that EZF regulated biological process to treat GIOP including response to inorganic substance, reactive oxygen species biosynthetic process, JAK-STAT cascade, response to nutrient levels, response to peptide, positive regulation of cell migration, and response to lipopolysaccharide (Figure 4). Besides, GO-MF analysis manifested that EZF regulated the molecular functions of heme binding and peptide hormone binding as shown in Figure 5. GO-CC analysis indicated that the regulation of the external side of plasma membrane, lytic vacuole, and receptor complex were beneficial to the therapeutic effect of EZF (Figure 6).

### 3.4. Network of “Chemical Components-Target Genes-Signaling Pathways”

**Figure 7:**
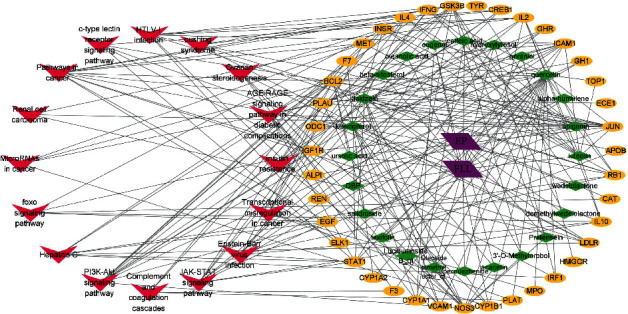
The “chemical components-target genes-signal pathway” network of EZF in treating osteoporosis. FLL: Fructus Ligustri Lucidi; EP: *Ecliptae herba*.

In order to elucidate the internal relationship between the chemical components, key targets, and signaling pathways of EZF clearly, the Cytoscape 3.6.0 software was successfully used to establish the “chemical components-target genes-signaling pathways” network (Figure 7).

**Figure 8:**
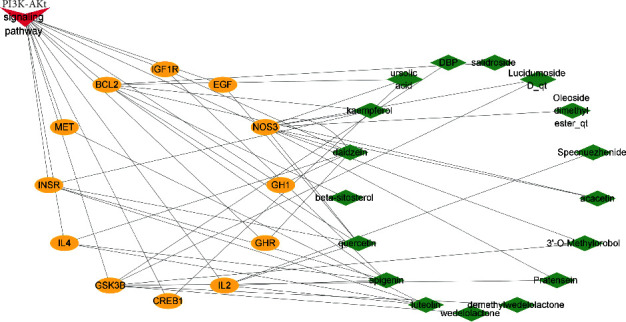
PI3K-Akt pathway in preventing osteoporosis mediated by EZF.

PI3k-Akt signaling pathway played an important role in cell signal transduction and metabolism [39]. Protein kinase B (PKB), also known as Akt, was a serine/threonine kinase [40] which protected cells from apoptosis followed by activating PI3K. Moreover, PI3K-Akt signaling pathway could reduce osteoblast apoptosis and prevent osteoporosis by antagonizing oxidative stress [41–43]. This result showed that there were 17 active components in EZF which acted on PI3K-Akt pathway (Figure 8), indicating that PI3K-Akt signaling pathway was one of the important regulatory pathways of EZF in antiosteoporosis.

### 3.5. Molecular Docking

**Figure 9:**
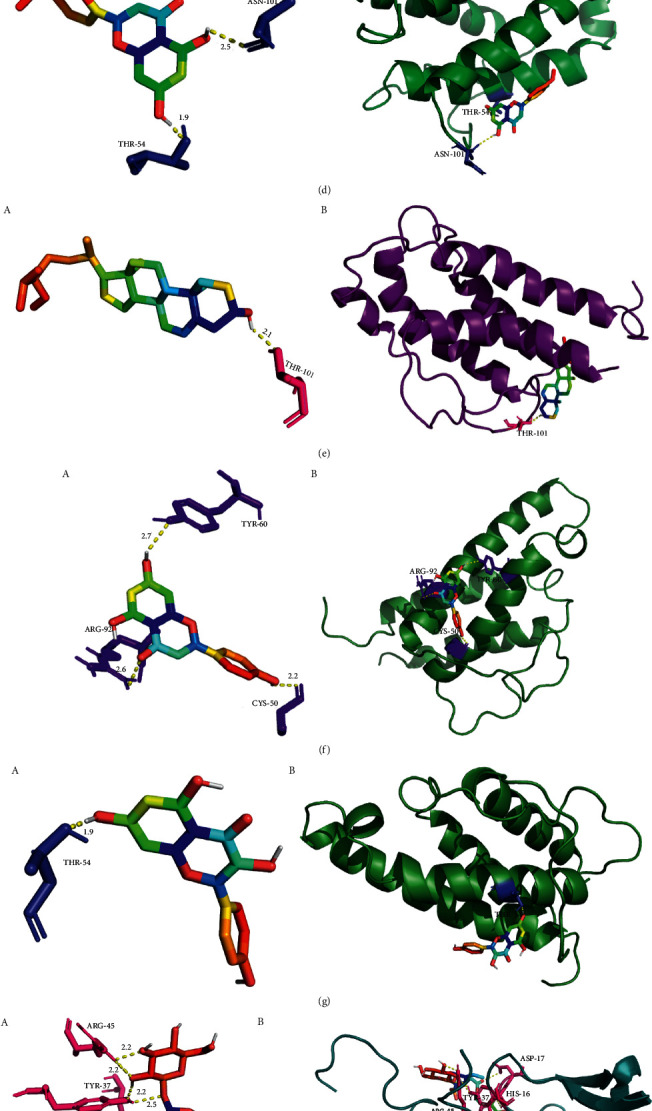
Top ten molecular dockings diagram of EZF for treating GIOP. (a) IL4, wedelolactone; (b) EGF, ursolic-acid; (c) IL2, ursolic acid; (d) IL4-, acacetin; (e) IL2, beta-sitosterol; (f) IL4, apigenin; (g) IL4, kaempferol; (h) EGF, specnuezhenide; (i) IL4, dimethyl wedelolactone; (j) IL4, specnuezhenide. “a” shows the amino acid residues and hydrogen bond lengths attached to the active component (ligand), where the colored rainbow represents the ligands and the pure color represents the amino acid residues. “b” is the complete docking of protein and ligand.

IL2, IL4, and EGF exist in both hub genes and PI3K-Akt signaling pathway (Figure 2(b) and Figure 8), so we selected the corresponding proteins of these genes as receptors. Similarly, we selected the 17 active components in EZF and PI3K-Akt signaling pathway as ligands. A total of 51 times were docked, and the results were put in Supplementary Files 1 and 2. Then we selected the top ten conformations with the lowest binding energy and optimal conformation to display (Table 3 and Figures 9(a)–9(j)). Through the docking results, we speculate that the components of EZF such as wedelolactone, dimethyl wedelolactone, specnuezhenide, ursolic acid, acacetin, beta-sitosterol, apigenin, and kaempferol play an important role in the treatment of GIOP.

### 3.6. Behavioral Observation of Zebrafish

**Figure 10:**
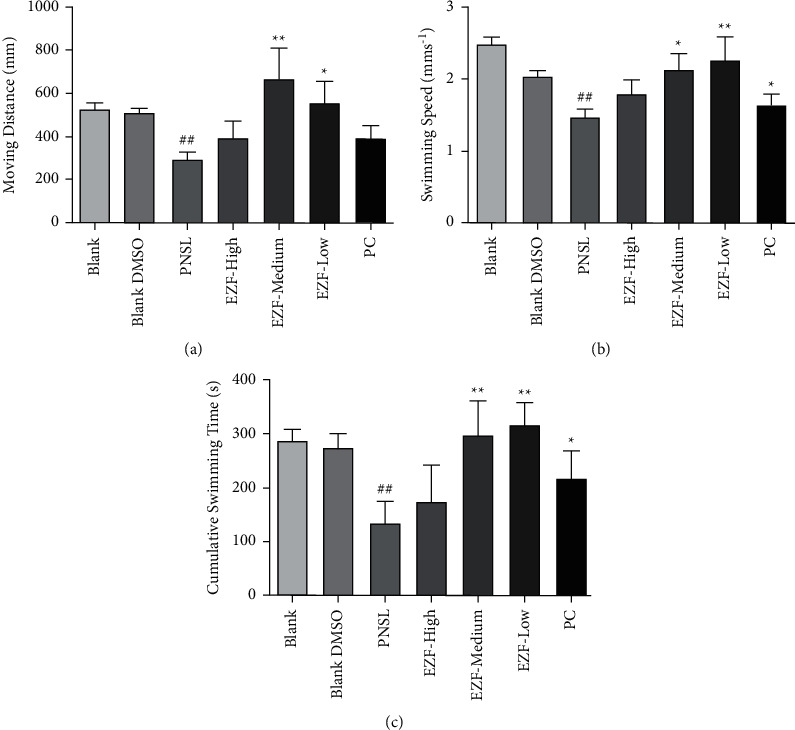
Effect of EZF extract on the behavior of GIOP zebrafish (*n* = 15). (a) The moving distance detected by animal behavior analyzer in each group. (b) The swimming speed detected by animal behavior analyzer in each group. (c) Cumulative swimming time detected by animal behavior analyzer in each group. Compared with the blank group, ^##^*P* < 0.01; compared with the model group, ^*∗*^*P* < 0.05 and ^*∗∗*^*P* < 0.01.

The moving distance, swimming speed, and cumulative swimming time of each group were obtained by Noldus DanioVision tracking chamber and EthoVision XT (Wageningen, Netherlands) version 8.0 monitoring software. Obviously, the activity of PNSL group more significantly decreased compared to that of the blank group and the blank DMSO group (*P* < 0.01). Compared with the model group, the moving distance, swimming speed, and cumulative swimming time of the EZF medium-dose group, EZF low-dose group, and and positive control group were significantly increased (*P* < 0.05), while there was no significant difference in these indexes between EZF high-dose group and model group. The quantitative results are shown in Figure 10.

### 3.7. Staining Observation

**Figure 11:**
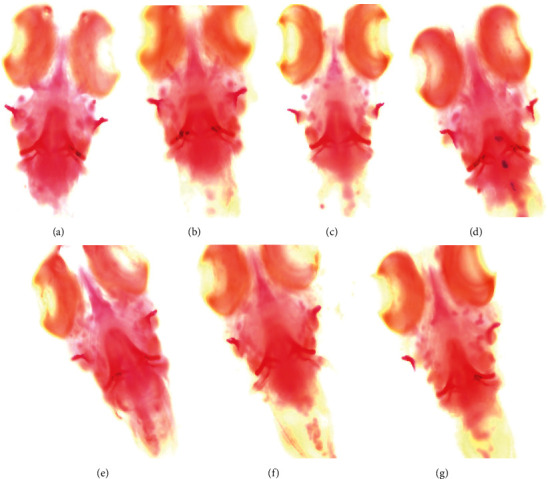
Reversal effect of EZF on bone mineralization in GIOP zebrafish (*n* = 15). (a) Blank control group. (b) Blank DMSO group. (c) Model group. (d) EZF high-dose group. (e) EZF medium-dose group. (f) EZF low-dose group. (g) Positive control group.

**Figure 12:**
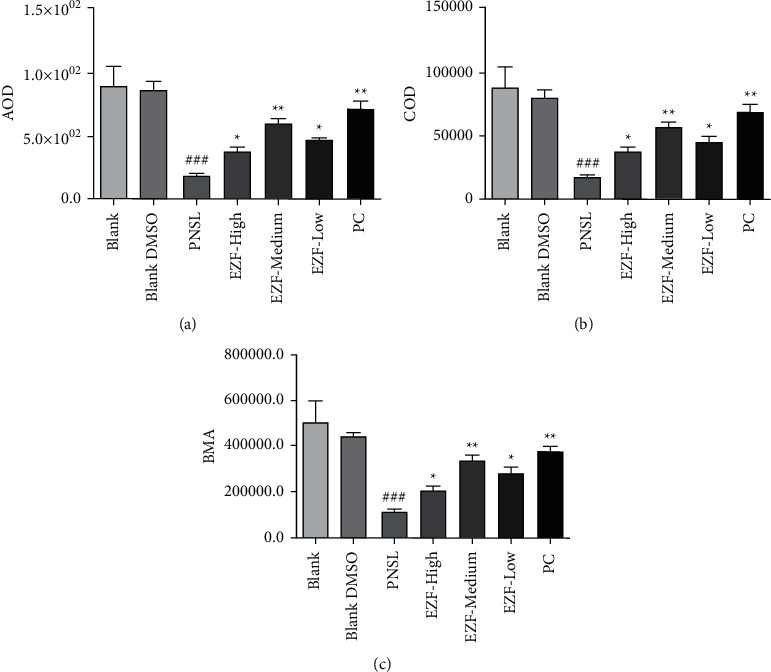
Quantitative analysis of the effect of EZF in reversing GIOP in zebrafish (*n* = 15). (a) The average optical density in different groups. (b) Cumulative optical density in different groups. (c) Staining area in different groups. Compared with the blank group, ^###^*P* < 0.001; compared with model group, ^*∗*^*P* < 0.05 and ^*∗∗*^*P* < 0.01.

According to microscopic observation (Olympus IX71/IX81, Olympus Corporation, Japan) of the staining results (Figure 11), the BMA, AOD, and COD were significantly decreased after the intervention of PNSL (*P* < 0.001) compared with the blank group. Compared with the model group, the BMA, AOD, and COD in the positive drug group were significantly improved (*P* < 0.01). Each group of EZF also had significant improvement (*P* < 0.05); in particular, the EZF medium-dose group had the most powerful effect in reversing GIOP (*P* < 0.01, Figure 12). The results suggest that medium dose of EZF may be the most appropriate concentration for the treatment of GIOP in zebrafish model.

## 4. Discussion

Hormone changes, physical disability, accelerated aging, and inflammation-related osteoclast activation were the main potential factors of osteoporosis [44, 45]. As known to all, the use of GCs was a recognized cause of secondary OP. GIOP led to decreased bone strength and osteoporotic fracture and further aggravated the dysfunction and disability of patients. A meta-analysis from randomized controlled trials and control groups found that the annual incidence of vertebral fractures was 2.8% to 8.2% in patients after taking oral GCs [46]. Zebrafish has many special advantages in establishing osteoporosis model, such as transparent embryo and rapid bone development. Most importantly, it is highly homologous with human related genes, tissues, organs, and development, in which the gene similarity is as high as 87% [47]. Therefore, zebrafish has significant advantages in the establishment of osteoporosis model. The inhibitory effect of GCs on bone formation has been clear, and it can induce an osteoporotic phenotype in many animal models, such as mouse, rat, rabbits, ewes, beagles, pigs, and zebrafish [48]. GCs had little effect on neurological, vascular, and muscular development of zebrafish. Therefore, they can be used to construct many pathological models. For example, sleep deprivation model was induced by light stimulation [49], depression model was induced by reserpine [50], thrombus model was induced by phenylhydrazine (PHZ) [51], epilepsy model was induced by pentylenetetrazol (PTZ) [52], vascular insufficiency model was induced by tyrosine kinase inhibitor [53], vascular defect model was induced by axitinib [54], and *sapje* strain model was always used for muscle atrophy [55]. GCs are only recognized to induce osteoporosis and cannot be used to induce other diseases in zebrafish. Therefore, GC is mainly used as a model drug to induce osteoporosis.

EZF had significantly antiosteoporotic effect on ovariectomized rats [39, 40]. In this paper, we focus on constructing the network of “chemical components-target genes-signaling pathways” through network pharmacology, so as to clarify its main mechanism in treating GIOP. Then the therapeutic effect of EZF was verified by GCs-induced zebrafish model. GCs had multiple pharmacological targets, so they had many advantages, including promoting osteoclast, inhibiting osteoblast, inhibiting the synthesis of sex hormone, and inhibiting the absorption of calcium and phosphorus [56–59]. This study found that the 39 common characteristic targets of EZF and GIOP are as follows: IL10, RB1, JUN, TOP1, ICAM1, IL2, TYR, IFNG, IL4, INSR, MET, F7, Bcl-2, PLAU, ODC1, IGF1R, ALPI REN, EGF, ELK1, STAT1, CYP1A2, F3, CYP1A1, VCAM1, NOS3, CYP1B1, PLAT, MPO, IRF1, HMGCR, LDLR, CAT, APOB, ECE1, GH1, GHR, CREB1, and GSK-3B. Through systematic literature search, it was found that IL10 decreased bone loss in inflammatory diseases [60, 61], the expression of Bcl-2 gene could inhibit the proliferation of osteoblasts and apoptosis of osteoclasts [62], and GSK-3*β* had an irreplaceable role in bone metabolism. Therefore, this work is consistent with the existing reports, and, more importantly, we have explored and predicted many targets that have not been reported.

Among the 24 constituents corresponding to 39 common targets, 19 compounds were in FLL and 5 compounds were obtained from EP. It had been reported that luteolin could inhibit the proliferation of osteoblasts by preventing the overproduction of ROS and then enhance the expression of osteoblastic markers to promote the differentiation of osteoblasts [63]. Apigenin could significantly reduce trabecular bone loss in OVX mice [64]. Quercetin widely existed in Chinese herbal medicine, which reduced osteoporosis induced by ovariectomy through regulating autophagy and apoptosis of rat osteoblasts [65]. EP and its component wedelolactone inhibited the proliferation and differentiation of osteoclasts [66]. These potential active ingredients contained in EZF had latent promising clinical application prospect for the curation of GIOP. Besides, the zebrafish experiment we chose further verified the pharmacological effect of EZF in reversing GIOP.

KEGG enrichment analysis screened out 4 signaling pathways with high correlation, which were PI3K-Akt, pathways in cancer, JAK-STAT, and AGE-RAGE signaling pathway. Among them, the correlation degree of PI3K-Akt signaling pathway was the highest one, which could be considered as a potential target [67–69]. Revealed research indicated that the protein expression of PI3K, p-AKT, and p-GSK-3 could significantly reverse the GIOP in mice [70]. Moreover, PI3K-Akt pathway could act on specific target genes such as FOXO and GSK-3*β* to reduce the oxidative damage of osteoblasts and osteoclasts [71]. Our present study listed out the 17 active components of EZF which could adjust 12 gene targets mediated by PI3K-Akt signaling pathway in the treatment of GIOP. Janus kinase (JAK)-signal transduction and activator of transcription (STAT) signaling pathway were important pathway that mediates the signal transduction of many cytokines, growth factors, and hormones [72, 73]. Recent reports indicate that inhibition of JAK-STAT can reconstruct normal bone mineral density in ovariectomized mice [74]. Some clinical diagnosis results of diabetic patients such as decreased bone mineral density, inhibition of bone turnover markers, and bone mass damage may be influenced by AGE-RAGE signaling pathway [75].

Among the 4 signaling pathways, PI3K-Akt signaling pathway shows the most obvious intensity. In this network (Figure 8), there were 17 active components and 3 hub genes (EGF, IL2, and IL4) related to it. By analyzing the results of 51 docking times, we can find that wedelolactone, dimethyl wedelolactone, specnuezhenide, ursolic acid, acacetin, beta-sitosterol, apigenin, and kaempferol had high activity. It had been reported that wedelolactone and dimethyl wedelolactone can enhance BMSC differentiation towards osteoblasts and promote bone formation [76]. Specnuezhenide is one of the iridoid glycosides and plays an important role in promoting the proliferation of bone marrow mesenchymal stem cells in vitro and inhibiting replicative aging [77]. Cao et al. reported that oleanolic acid and ursolic acid, as the active components of FLL, had a beneficial effect on calcium balance and calcium stimulating hormone circulation level, so it is expected to become a candidate drug for the prevention and treatment of osteoporosis [78]. Acacetin, beta-sitosterol, apigenin, and kaempferol, as active components of Chinese medicine, have been screened and reported for many times in the treatment of many diseases [79, 80]. In addition, EZF was quantitatively analyzed by HPLC, and the contents of active components such as specnuezhenide and wedelolactone were detected. The related results are shown in Supplementary File 3. The prediction with network pharmacology helped us accurately locate the molecular mechanism of EZF in antiosteoporosis.

Despite the wide spread of this technology, network pharmacology still has some limitations. Firstly, the existing databases are not complete enough, so we have to use different platforms to improve the accuracy of researchers' speculation. Secondly, different model algorithms have been formed and developed in Cytoscape and Autodock, which may cause the experiment to be more difficult. Hence, it is necessary to select appropriate algorithms according to different purposes to ensure the accuracy of the results. Thirdly, the method is mainly used in qualitative research. It cannot be ignored that there is a dose-response relationship between drugs and diseases, but the current network pharmacology is still difficult to quantify the target compounds [81]. In the future, combining the development of instrument analysis with data analysis, we hope to find a fast and nondestructive method to effectively solve the above limitations. It is expected to be widely used in the development of ancient classic prescriptions.

## 5. Conclusion

EZF has a good effect on reversing the inhibition of glucocorticoid-induced bone formation. The 24 components of EZF were found to be the material basis of antiosteoporosis by regulating 39 related gene targets and multiple signaling pathways.

## Figures and Tables

**Table 1:** Chemical components corresponding to gene targets of EZF.

Source	Chemical components	Gene targets
FLL	Luteolin	IL10, RB1, JUN, TOP1, ICAM1, IL2, TYR, IFNG, IL4, INSR, MET
FLL	Apigenin	F7, Bcl-2, PLAU, RB1, JUN, ODC1, IGF1R, ICAM1, IL2, IFNG, IL4, INSR, ALPI
FLL	Alpha-humulene	REN
FLL	Quercetin	F7, Bcl-2, PLAU, IL10, EGF, RB1, JUN, ELK1, ODC1, TOP1, STAT1, CYP1A2, F3, CYP1A1, ICAM1, VCAM1, NOS3, IL2, CYP1B1, PLAT, IFNG, MPO, INSR, IRF1
FLL	Geraniol	HMGCR
FLL	Hydroxytyrosol	PLAU, STAT1, IRF1
FLL	Caffeic acid	PLAU, CYP1A1
FLL	Eugenol	PLAU, CYP1A1, CYP1B1
FLL	Oleanolic acid	ICAM1
FLL	Beta-sitosterol	Bcl-2, JUN
FLL	Daidzein	JUN, NOS3, LDLR, CAT, IGF1R, STAT1, ICAM1, APOB, VCAM1, NOS3, ECE1, IL4, GH1, GHR
FLL	Kaempferol	NOS3, F7, Bcl-2, JUN, STAT1, CYP1A2, CYP1A1, ICAM1, VCAM1, CYP1B1, INSR
FLL	Ursolic acid	PLAU, Bcl-2, JUN, ICAM1, CREB1, NOS3
FLL	DBP	GSK-3*β*
FLL	Salidroside	Bcl-2
FLL	Taxifolin	ICAM1, APOB
FLL	Lucidumoside D_qt (灵芝苷)	NOS3, GSK-3*β*
FLL	Oleoside dimethyl ester_qt (油苷二甲酯)	NOS3
FLL	Specnuezhenide	IL2, TYR
EP	Acacetin	NOS3, Bcl-2
EP	3′-O-Methylorobol (3′-O-甲酚)	GSK-3*β*, NOS3
EP	Pratensein (红三叶草素)	NOS3, GSK-3*β*
EP	Demethyl wedelolactone (去甲基蟛蜞菊内酯)	GSK-3*β*
EP	Wedelolactone (蟛蜞菊内酯)	GSK-3*β*

**Table 2:** Top 10 hub genes ranked by MCC method.

Rank	Name	Score
1	IL10	1146366
2	ICAM1	1140888
3	JUN	1135839
4	EGF	1056786
5	IFNG	1049040
6	VCAM1	979608
7	IL4	974160
8	MPO	897120
9	CAT	856930
10	IL2	605522

**Table 3:** Top 10 molecular dockings optimal binding energy.

Rank	Proteins and compounds	Mode	Affinity (kcal/mol)	Dist from rmsd	Best mode rmsd
1	IL4, wedelolactone	1	−7.6	0.000	0.000
2	EGF, ursolic acid	1	−7.6	0.000	0.000
3	IL2, ursolic acid	1	−7.5	0.000	0.000
4	IL4, acacetin	1	−7.3	0.000	0.000
5	IL2, beta-sitosterol	1	−7.3	0.000	0.000
6	IL4, apigenin	1	−7.2	0.000	0.000
7	IL4, kaempferol	1	−7.2	0.000	0.000
8	EGF, specnuezhenide	1	−7.1	0.000	0.000
9	IL4, demethyl wedelolactone	1	−7.1	0.000	0.000
10	IL4, specnuezhenide	1	−7.1	0.000	0.000

## Data Availability

The data used to support the findings of this study are available from the corresponding author upon request.

## Supplementary Materials

Supplementary Materials Supplementary File 1: 51 molecular docking results. Supplementary File 2: 51 molecular docking diagrams of EZF for treating GIOP. Supplementary File 3: determination of active components in EZF by HPLC.
