# Supplementary material for: Elaborate the Mechanism of Ancient Classic Prescriptions (Erzhi Formula) in Reversing GIOP by Network Pharmacology Coupled with Zebrafish Verification
Source: Evid Based Complement Alternat Med. 2022 Jan 10;2022:7019792. doi: 10.1155/2022/7019792 (PMC8763506; doi:10.1155/2022/7019792)
Supplement: Supplementary Materials — Supplementary File 1: 51 molecular docking results. Supplementary File 2: 51 molecular docking diagrams of EZF for treating GIOP. Supplementary File 3: determination of active components in EZF by HPLC. [file 7019792.f1.zip › 7019792.f1/Supplementary File 1.docx]

51 molecular docking results

1. EGF---3'-O-Methylorobol

mode | affinity | dist from best mode

| (kcal/mol) | rmsd l.b.| rmsd u.b.

-----+------------+----------+----------

1 -6.2 0.000 0.000

2 -6.0 10.908 14.060

3 -5.8 11.658 16.946

4 -5.8 1.175 2.840

5 -5.8 16.620 19.905

6 -5.8 1.315 7.366

7 -5.7 7.142 11.196

8 -5.7 12.566 16.465

9 -5.6 11.010 13.496

2. EGF---acacetin

mode | affinity | dist from best mode

| (kcal/mol) | rmsd l.b.| rmsd u.b.

-----+------------+----------+----------

1 -6.4 0.000 0.000

2 -6.3 13.236 15.624

3 -6.0 16.843 17.817

4 -5.9 16.560 17.698

5 -5.7 12.417 14.993

6 -5.7 8.651 11.074

7 -5.6 13.494 14.473

8 -5.6 17.539 21.000

9 -5.6 13.688 15.684

3. EGF---apigenin

mode | affinity | dist from best mode

| (kcal/mol) | rmsd l.b.| rmsd u.b.

-----+------------+----------+----------

1 -6.5 0.000 0.000

2 -6.1 13.335 15.496

3 -6.0 21.858 22.605

4 -6.0 17.986 19.911

5 -5.8 12.205 17.140

6 -5.8 13.084 18.181

7 -5.7 16.043 17.247

8 -5.6 13.067 14.293

9 -5.6 12.535 14.93

4. EGF---beta-sitosterol

mode | affinity | dist from best mode

| (kcal/mol) | rmsd l.b.| rmsd u.b.

-----+------------+----------+----------

1 -6.1 0.000 0.000

2 -6.1 1.542 3.011

3 -6.0 1.696 3.358

4 -5.9 4.687 9.422

5 -5.8 4.474 6.730

6 -5.7 1.279 1.794

7 -5.7 23.414 27.502

8 -5.6 3.406 9.060

9 -5.6 3.773 9.402

5. EGF---daidzein

mode | affinity | dist from best mode

| (kcal/mol) | rmsd l.b.| rmsd u.b.

-----+------------+----------+----------

1 -5.9 0.000 0.000

2 -5.9 13.209 15.559

3 -5.8 12.675 14.663

4 -5.7 20.109 23.368

5 -5.6 3.765 5.725

6 -5.6 2.340 6.771

7 -5.5 5.043 10.411

8 -5.5 3.255 5.070

9 -5.4 29.418 32.024

6. EGF---DBP

mode | affinity | dist from best mode

| (kcal/mol) | rmsd l.b.| rmsd u.b.

-----+------------+----------+----------

1 -4.8 0.000 0.000

2 -4.8 0.971 3.843

3 -4.7 14.165 16.393

4 -4.6 1.731 2.597

5 -4.6 14.114 16.284

6 -4.6 1.688 3.785

7 -4.5 1.346 2.503

8 -4.3 15.128 18.425

9 -4.3 14.550 17.828

7. EGF---demethylwedelolactone

mode | affinity | dist from best mode

| (kcal/mol) | rmsd l.b.| rmsd u.b.

-----+------------+----------+----------

1 -6.6 0.000 0.000

2 -6.4 13.758 14.521

3 -6.4 1.347 6.214

4 -6.3 13.531 15.607

5 -6.3 13.523 14.991

6 -6.3 12.020 16.329

7 -6.3 1.237 6.771

8 -6.3 11.898 16.351

9 -6.2 7.319 9.777

8. EGF---kaempferol

mode | affinity | dist from best mode

| (kcal/mol) | rmsd l.b.| rmsd u.b.

-----+------------+----------+----------

1 -6.8 0.000 0.000

2 -6.2 13.470 15.653

3 -5.8 27.892 29.773

4 -5.8 24.195 28.133

5 -5.8 2.539 3.173

6 -5.8 5.840 8.485

7 -5.7 2.233 6.179

8 -5.7 14.532 15.801

9 -5.6 8.415 10.467

9. EGF---LucidumosideD_qt

mode | affinity | dist from best mode

| (kcal/mol) | rmsd l.b.| rmsd u.b.

-----+------------+----------+----------

1 -6.5 0.000 0.000

2 -6.5 2.399 10.139

3 -6.3 9.446 14.350

4 -6.3 15.492 20.132

5 -6.2 1.806 2.616

6 -6.2 16.231 19.727

7 -6.1 4.022 9.028

8 -6.0 8.322 13.384

9 -6.0 2.447 3.417

10. EGF---luteolin

mode | affinity | dist from best mode

| (kcal/mol) | rmsd l.b.| rmsd u.b.

-----+------------+----------+----------

1 -6.6 0.000 0.000

2 -6.3 13.010 15.443

3 -6.2 1.323 2.813

4 -6.2 13.183 14.483

5 -6.1 13.819 14.878

6 -5.9 13.841 15.879

7 -5.9 12.595 17.746

8 -5.9 13.142 16.956

9 -5.8 11.713 16.803

11. EGF---Oleoside-dimethyl ester-qt

mode | affinity | dist from best mode

| (kcal/mol) | rmsd l.b.| rmsd u.b.

-----+------------+----------+----------

1 -6.1 0.000 0.000

2 -6.1 4.871 7.421

3 -5.9 17.847 20.377

4 -5.9 8.832 14.737

5 -5.8 20.353 24.404

6 -5.6 18.102 20.349

7 -5.5 1.572 1.963

8 -5.5 7.524 11.296

9 -5.5 5.863 9.186

12. EGF---Pratensein

mode | affinity | dist from best mode

| (kcal/mol) | rmsd l.b.| rmsd u.b.

-----+------------+----------+----------

1 -6.4 0.000 0.000

2 -6.1 1.875 7.303

3 -5.9 16.297 18.525

4 -5.9 1.472 7.465

5 -5.9 14.310 16.745

6 -5.9 17.002 17.883

7 -5.8 14.061 15.687

8 -5.8 14.947 17.231

9 -5.8 3.429 7.931

13. EGF---quercetin

mode | affinity | dist from best mode

| (kcal/mol) | rmsd l.b.| rmsd u.b.

-----+------------+----------+----------

1 -6.6 0.000 0.000

2 -6.6 0.736 1.467

3 -6.4 13.130 15.231

4 -6.1 2.610 3.350

5 -6.0 3.623 7.150

6 -5.9 23.879 27.844

7 -5.9 13.085 15.408

8 -5.8 2.389 3.759

9 -5.8 14.364 15.690

14. EGF---salidroside

mode | affinity | dist from best mode

| (kcal/mol) | rmsd l.b.| rmsd u.b.

-----+------------+----------+----------

1 -5.9 0.000 0.000

2 -5.8 16.222 17.191

3 -5.7 15.833 18.165

4 -5.7 24.166 26.452

5 -5.6 16.228 18.261

6 -5.5 15.889 18.150

7 -5.5 11.791 14.817

8 -5.5 25.717 27.902

9 -5.5 15.774 17.534

15. EGF---Specnuezhenide

mode | affinity | dist from best mode

| (kcal/mol) | rmsd l.b.| rmsd u.b.

-----+------------+----------+----------

1 -7.1 0.000 0.000

2 -6.8 1.649 4.436

3 -6.7 16.619 20.718

4 -6.7 16.076 20.006

5 -6.7 2.135 10.878

6 -6.6 6.520 12.294

7 -6.6 16.042 21.151

8 -6.6 2.809 5.779

9 -6.6 15.287 18.268

16. EGF---ursolic-acid

mode | affinity | dist from best mode

| (kcal/mol) | rmsd l.b.| rmsd u.b.

-----+------------+----------+----------

1 -7.6 0.000 0.000

2 -7.3 11.692 15.341

3 -7.3 17.805 20.293

4 -7.2 12.292 14.133

5 -7.0 12.390 14.550

6 -6.9 17.697 20.983

7 -6.8 13.496 15.490

8 -6.7 17.809 20.568

9 -6.5 16.919 19.147

17. EGF---wedelolactone

mode | affinity | dist from best mode

| (kcal/mol) | rmsd l.b.| rmsd u.b.

-----+------------+----------+----------

1 -6.5 0.000 0.000

2 -6.4 13.631 15.105

3 -6.3 7.355 12.137

4 -6.3 13.897 14.524

5 -6.2 24.428 27.421

6 -6.2 12.136 16.368

7 -6.2 6.487 11.358

8 -6.2 14.236 15.856

9 -6.1 1.595 6.683

18. IL-2---3'-O-Methylorobol

mode | affinity | dist from best mode

| (kcal/mol) | rmsd l.b.| rmsd u.b.

-----+------------+----------+----------

1 -6.8 0.000 0.000

2 -6.3 2.346 3.923

3 -6.1 9.867 10.732

4 -6.1 18.647 20.385

5 -6.1 34.282 37.405

6 -6.0 34.446 37.627

7 -5.9 20.457 25.145

8 -5.9 1.421 3.179

9 -5.8 15.919 20.275

19. IL-2---acacetin

mode | affinity | dist from best mode

| (kcal/mol) | rmsd l.b.| rmsd u.b.

-----+------------+----------+----------

1 -6.6 0.000 0.000

2 -6.4 3.167 6.824

3 -6.4 8.247 8.664

4 -6.3 8.064 10.699

5 -6.2 3.865 5.711

6 -6.2 11.116 13.495

7 -6.1 2.388 7.191

8 -6.0 23.493 25.302

9 -6.0 23.602 25.499

20. IL-2---apigenin

mode | affinity | dist from best mode

| (kcal/mol) | rmsd l.b.| rmsd u.b.

-----+------------+----------+----------

1 -6.7 0.000 0.000

2 -6.4 35.334 37.540

3 -6.4 2.724 6.323

4 -6.4 8.135 8.535

5 -6.3 2.798 4.952

6 -6.1 35.478 37.562

7 -6.1 23.193 24.978

8 -6.1 2.277 6.324

9 -5.9 7.557 8.395

21. IL-2---beta-sitosterol

mode | affinity | dist from best mode

| (kcal/mol) | rmsd l.b.| rmsd u.b.

-----+------------+----------+----------

1 -7.3 0.000 0.000

2 -7.1 1.423 3.070

3 -6.7 2.319 3.888

4 -6.6 3.239 8.377

5 -6.5 2.629 4.038

6 -6.4 26.452 28.726

7 -6.4 24.213 27.317

8 -6.4 3.505 8.798

9 -6.2 24.195 27.064

22. IL-2---daidzein

mode | affinity | dist from best mode

| (kcal/mol) | rmsd l.b.| rmsd u.b.

-----+------------+----------+----------

1 -6.2 0.000 0.000

2 -6.2 34.429 37.018

3 -6.1 23.310 24.906

4 -5.9 23.098 24.672

5 -5.9 2.981 6.782

6 -5.8 7.593 8.281

7 -5.8 3.196 4.755

8 -5.7 35.558 37.942

9 -5.6 22.834 24.185

23. IL-2---DBP

mode | affinity | dist from best mode

| (kcal/mol) | rmsd l.b.| rmsd u.b.

-----+------------+----------+----------

1 -5.1 0.000 0.000

2 -5.0 1.976 2.873

3 -4.9 2.507 4.274

4 -4.9 2.444 4.750

5 -4.9 2.549 5.377

6 -4.9 1.823 3.559

7 -4.8 21.936 23.688

8 -4.8 1.750 3.957

9 -4.7 23.219 25.124

24. IL-2---demethylwedelolactone

mode | affinity | dist from best mode

| (kcal/mol) | rmsd l.b.| rmsd u.b.

-----+------------+----------+----------

1 -6.9 0.000 0.000

2 -6.6 34.640 36.941

3 -6.5 2.122 7.182

4 -6.5 34.603 37.240

5 -6.4 2.184 4.407

6 -6.4 20.154 24.139

7 -6.2 33.525 35.732

8 -6.2 20.107 24.048

9 -6.0 9.970 10.176

25. IL-2---kaempferol

mode | affinity | dist from best mode

| (kcal/mol) | rmsd l.b.| rmsd u.b.

-----+------------+----------+----------

1 -6.7 0.000 0.000

2 -6.4 35.588 37.531

3 -6.1 2.811 6.403

4 -6.1 35.651 37.510

5 -6.0 1.790 6.809

6 -6.0 22.819 24.671

7 -5.9 1.844 6.755

8 -5.9 22.875 24.822

9 -5.9 2.395 3.105

26. IL-2---Lucidumoside D-qt

mode | affinity | dist from best mode

| (kcal/mol) | rmsd l.b.| rmsd u.b.

-----+------------+----------+----------

1 -6.5 0.000 0.000

2 -6.4 1.867 3.255

3 -6.4 3.098 7.769

4 -6.4 34.659 39.421

5 -6.4 1.367 2.724

6 -6.4 34.460 38.553

7 -6.2 5.174 9.042

8 -6.2 1.794 3.994

9 -6.1 1.970 4.114

27. IL-2---luteolin

mode | affinity | dist from best mode

| (kcal/mol) | rmsd l.b.| rmsd u.b.

-----+------------+----------+----------

1 -6.7 0.000 0.000

2 -6.5 35.725 38.125

3 -6.5 23.484 25.510

4 -6.3 7.774 8.442

5 -6.3 35.623 37.985

6 -6.1 30.588 32.333

7 -6.0 14.409 17.845

8 -5.9 35.578 37.735

9 -5.9 22.333 24.281

28. IL-2---Oleoside-dimethyl ester-qt

mode | affinity | dist from best mode

| (kcal/mol) | rmsd l.b.| rmsd u.b.

-----+------------+----------+----------

1 -5.6 0.000 0.000

2 -5.5 2.700 8.253

3 -5.4 35.440 38.242

4 -5.3 4.210 8.644

5 -5.3 3.552 7.034

6 -5.3 28.055 31.064

7 -5.2 3.206 8.077

8 -5.2 2.312 4.499

9 -5.2 3.803 8.392

29. IL-2---Pratensein

mode | affinity | dist from best mode

| (kcal/mol) | rmsd l.b.| rmsd u.b.

-----+------------+----------+----------

1 -6.8 0.000 0.000

2 -6.4 2.215 3.809

3 -6.1 1.426 2.836

4 -6.1 20.752 25.310

5 -6.0 17.864 20.959

6 -5.9 34.619 37.542

7 -5.9 34.590 37.624

8 -5.9 18.723 20.325

9 -5.9 9.789 10.583

30. IL-2---quercetin

mode | affinity | dist from best mode

| (kcal/mol) | rmsd l.b.| rmsd u.b.

-----+------------+----------+----------

1 -6.8 0.000 0.000

2 -6.7 35.831 37.873

3 -6.6 2.841 2.931

4 -6.6 2.049 3.608

5 -6.4 1.880 6.692

6 -6.4 4.128 6.180

7 -6.3 2.462 7.017

8 -6.1 12.623 14.749

9 -6.1 21.054 23.741

31. IL-2---salidroside

mode | affinity | dist from best mode

| (kcal/mol) | rmsd l.b.| rmsd u.b.

-----+------------+----------+----------

1 -6.6 0.000 0.000

2 -6.3 4.714 7.452

3 -5.9 5.831 7.480

4 -5.6 25.195 28.489

5 -5.6 31.636 34.045

6 -5.5 24.008 25.854

7 -5.5 30.911 33.058

8 -5.5 21.162 24.193

9 -5.4 21.269 25.039

32. IL-2---Specnuezhenide

mode | affinity | dist from best mode

| (kcal/mol) | rmsd l.b.| rmsd u.b.

-----+------------+----------+----------

1 -6.9 0.000 0.000

2 -6.9 3.974 7.041

3 -6.8 35.405 40.261

4 -6.8 3.008 6.097

5 -6.7 1.535 2.240

6 -6.6 7.516 13.691

7 -6.5 23.897 28.313

8 -6.5 2.920 5.323

9 -6.4 6.766 10.446

33. IL-2---ursolic acid

mode | affinity | dist from best mode

| (kcal/mol) | rmsd l.b.| rmsd u.b.

-----+------------+----------+----------

1 -7.5 0.000 0.000

2 -7.5 16.511 21.298

3 -7.4 24.644 27.700

4 -7.1 16.340 20.709

5 -7.1 3.465 8.015

6 -6.8 22.501 24.759

7 -6.8 26.128 28.787

8 -6.8 25.743 28.687

9 -6.8 25.417 28.409

34. IL-2---wedelolactone

mode | affinity | dist from best mode

| (kcal/mol) | rmsd l.b.| rmsd u.b.

-----+------------+----------+----------

1 -6.8 0.000 0.000

2 -6.6 1.768 7.149

3 -6.6 34.824 36.785

4 -6.5 2.544 4.190

5 -6.4 4.159 6.398

6 -6.2 10.878 13.458

7 -6.2 20.062 22.737

8 -6.1 30.328 34.840

9 -6.0 20.390 22.233

35. IL-4---3'-O-Methylorobol

mode | affinity | dist from best mode

| (kcal/mol) | rmsd l.b.| rmsd u.b.

-----+------------+----------+----------

1 -6.4 0.000 0.000

2 -6.1 27.227 29.787

3 -6.0 30.362 33.030

4 -5.9 28.814 30.714

5 -5.9 28.819 29.734

6 -5.8 28.442 30.333

7 -5.7 28.563 30.593

8 -5.7 17.503 19.607

9 -5.7 22.422 23.602

36. IL-4---acacetin

mode | affinity | dist from best mode

| (kcal/mol) | rmsd l.b.| rmsd u.b.

-----+------------+----------+----------

1 -7.3 0.000 0.000

2 -6.9 1.711 7.005

3 -6.6 22.391 23.563

4 -6.4 5.455 7.576

5 -6.4 15.100 18.328

6 -6.4 23.154 24.031

7 -6.4 24.143 24.895

8 -6.1 14.255 17.365

9 -5.9 15.199 17.560

37. IL-4---apigenin

mode | affinity | dist from best mode

| (kcal/mol) | rmsd l.b.| rmsd u.b.

-----+------------+----------+----------

1 -7.2 0.000 0.000

2 -7.2 1.656 6.476

3 -6.6 22.161 22.959

4 -6.3 14.908 18.136

5 -6.3 23.346 24.755

6 -6.2 20.975 22.300

7 -6.2 24.599 25.372

8 -6.2 16.259 18.346

9 -6.1 23.612 25.276

38. IL-4---beta-sitosterol

mode | affinity | dist from best mode

| (kcal/mol) | rmsd l.b.| rmsd u.b.

-----+------------+----------+----------

1 -6.8 0.000 0.000

2 -6.6 23.418 25.396

3 -6.5 13.267 17.792

4 -6.2 23.494 25.883

5 -6.2 22.759 25.684

6 -6.1 23.474 25.483

7 -6.0 4.277 10.807

8 -5.9 3.618 6.559

9 -5.9 4.377 6.895

39. IL-4---daidzein

mode | affinity | dist from best mode

| (kcal/mol) | rmsd l.b.| rmsd u.b.

-----+------------+----------+----------

1 -6.3 0.000 0.000

2 -6.3 12.159 13.909

3 -6.3 24.148 25.485

4 -6.1 23.592 25.429

5 -6.1 12.040 13.636

6 -6.1 31.485 34.105

7 -6.1 3.414 7.947

8 -6.0 0.917 2.220

9 -5.8 23.682 25.510

40. IL-4---DBP

mode | affinity | dist from best mode

| (kcal/mol) | rmsd l.b.| rmsd u.b.

-----+------------+----------+----------

1 -4.8 0.000 0.000

2 -4.7 24.021 26.089

3 -4.6 1.485 2.349

4 -4.5 2.055 3.836

5 -4.3 2.054 5.198

6 -4.3 3.295 7.202

7 -4.3 26.095 28.577

8 -4.2 2.153 4.201

9 -4.2 30.064 32.430

41. IL-4---demethylwedelolactone

mode | affinity | dist from best mode

| (kcal/mol) | rmsd l.b.| rmsd u.b.

-----+------------+----------+----------

1 -7.1 0.000 0.000

2 -6.6 22.905 26.561

3 -6.5 26.137 27.111

4 -6.4 20.436 24.317

5 -6.3 11.808 13.734

6 -6.3 26.357 27.135

7 -6.2 26.904 27.266

8 -6.2 2.053 2.840

9 -6.2 12.252 15.180

42. IL-4---kaempferol

mode | affinity | dist from best mode

| (kcal/mol) | rmsd l.b.| rmsd u.b.

-----+------------+----------+----------

1 -7.2 0.000 0.000

2 -6.8 1.418 6.352

3 -6.8 21.365 22.733

4 -6.4 14.171 15.967

5 -6.3 16.242 17.560

6 -6.2 20.394 21.531

7 -6.0 23.722 25.237

8 -6.0 23.129 25.046

9 -5.9 20.019 21.288

43. IL-4---LucidumosideD-qt

mode | affinity | dist from best mode

| (kcal/mol) | rmsd l.b.| rmsd u.b.

-----+------------+----------+----------

1 -6.2 0.000 0.000

2 -6.2 28.205 32.383

3 -5.9 1.616 2.233

4 -5.9 26.187 30.893

5 -5.8 3.614 9.747

6 -5.7 26.109 30.609

7 -5.7 2.346 4.092

8 -5.7 30.342 34.194

9 -5.6 27.148 30.260

44. IL-4---luteolin

mode | affinity | dist from best mode

| (kcal/mol) | rmsd l.b.| rmsd u.b.

-----+------------+----------+----------

1 -6.7 0.000 0.000

2 -6.6 12.443 15.212

3 -6.5 31.587 34.740

4 -6.5 31.332 33.968

5 -6.4 28.500 29.678

6 -6.3 20.198 21.351

7 -6.3 1.581 6.890

8 -6.3 31.456 34.121

9 -6.2 28.459 29.418

45. IL-4---Oleoside-dimethy lester-qt

mode | affinity | dist from best mode

| (kcal/mol) | rmsd l.b.| rmsd u.b.

-----+------------+----------+----------

1 -6.0 0.000 0.000

2 -5.9 27.576 30.034

3 -5.9 29.278 31.700

4 -5.8 28.237 30.739

5 -5.6 1.223 2.343

6 -5.5 2.413 8.358

7 -5.5 30.159 32.613

8 -5.4 2.225 3.776

9 -5.3 11.721 14.556

46. IL-4---Pratensein

mode | affinity | dist from best mode

| (kcal/mol) | rmsd l.b.| rmsd u.b.

-----+------------+----------+----------

1 -6.5 0.000 0.000

2 -6.2 12.072 12.638

3 -6.1 15.076 15.268

4 -6.1 3.112 7.315

5 -6.0 14.209 15.474

6 -6.0 11.781 12.084

7 -5.9 28.798 31.760

8 -5.8 2.552 3.509

9 -5.8 2.880 7.856

47. IL-4---quercetin

mode | affinity | dist from best mode

| (kcal/mol) | rmsd l.b.| rmsd u.b.

-----+------------+----------+----------

1 -7.0 0.000 0.000

2 -6.5 12.105 14.079

3 -6.5 30.803 33.271

4 -6.3 12.177 14.103

5 -6.1 32.026 34.161

6 -6.0 12.506 14.824

7 -5.9 31.930 34.381

8 -5.9 2.494 4.166

9 -5.8 2.866 8.149

48. IL-4---salidroside

mode | affinity | dist from best mode

| (kcal/mol) | rmsd l.b.| rmsd u.b.

-----+------------+----------+----------

1 -6.9 0.000 0.000

2 -6.2 4.779 8.993

3 -6.2 2.089 7.122

4 -6.2 5.424 10.016

5 -5.9 1.572 2.212

6 -5.8 14.851 17.863

7 -5.8 1.526 2.256

8 -5.5 5.001 8.874

9 -5.4 4.793 8.639

49. IL-4---Specnuezhenide

mode | affinity | dist from best mode

| (kcal/mol) | rmsd l.b.| rmsd u.b.

-----+------------+----------+----------

1 -7.1 0.000 0.000

2 -6.9 1.583 2.299

3 -6.7 3.008 10.792

4 -6.7 18.169 22.063

5 -6.6 3.694 8.247

6 -6.6 15.915 20.188

7 -6.6 26.652 29.746

8 -6.6 25.773 30.528

9 -6.6 25.753 29.188

50. IL-4---ursolic-acid

mode | affinity | dist from best mode

| (kcal/mol) | rmsd l.b.| rmsd u.b.

-----+------------+----------+----------

1 -7.0 0.000 0.000

2 -6.7 14.377 17.040

3 -6.5 29.144 33.230

4 -6.3 28.715 31.940

5 -6.3 13.563 16.955

6 -6.0 30.236 33.398

7 -6.0 2.289 8.237

8 -5.9 16.267 20.015

9 -5.9 16.983 20.222

51. IL-4---wedelolactone

mode | affinity | dist from best mode

| (kcal/mol) | rmsd l.b.| rmsd u.b.

-----+------------+----------+----------

1 -7.6 0.000 0.000

2 -7.3 1.100 6.506

3 -6.6 22.763 23.858

4 -6.6 21.729 23.169

5 -6.5 20.544 21.647

6 -6.3 24.873 25.373

7 -6.3 15.436 18.141

8 -6.2 13.322 17.340

9 -6.2 15.953 18.231
