# Supplementary material for: Elaborate the Mechanism of Ancient Classic Prescriptions (Erzhi Formula) in Reversing GIOP by Network Pharmacology Coupled with Zebrafish Verification
Source: Evid Based Complement Alternat Med. 2022 Jan 10;2022:7019792. doi: 10.1155/2022/7019792 (PMC8763506; doi:10.1155/2022/7019792)

**51 molecular docking diagrams of EZF for treating GIOP**

Pictures on the left show the amino acid residues and hydrogen bond lengths attached to the active component (ligand), where the colored rainbow are the ligands and the pure color are the amino acid residues. The right one is the complete docking of protein and ligand.

1. EGF---3'-O-Methylorobol


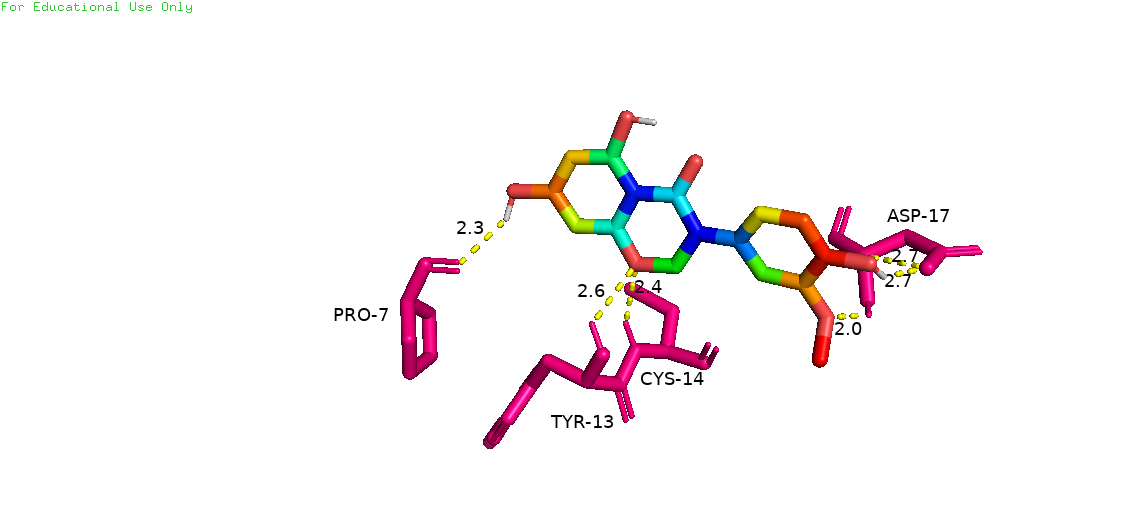

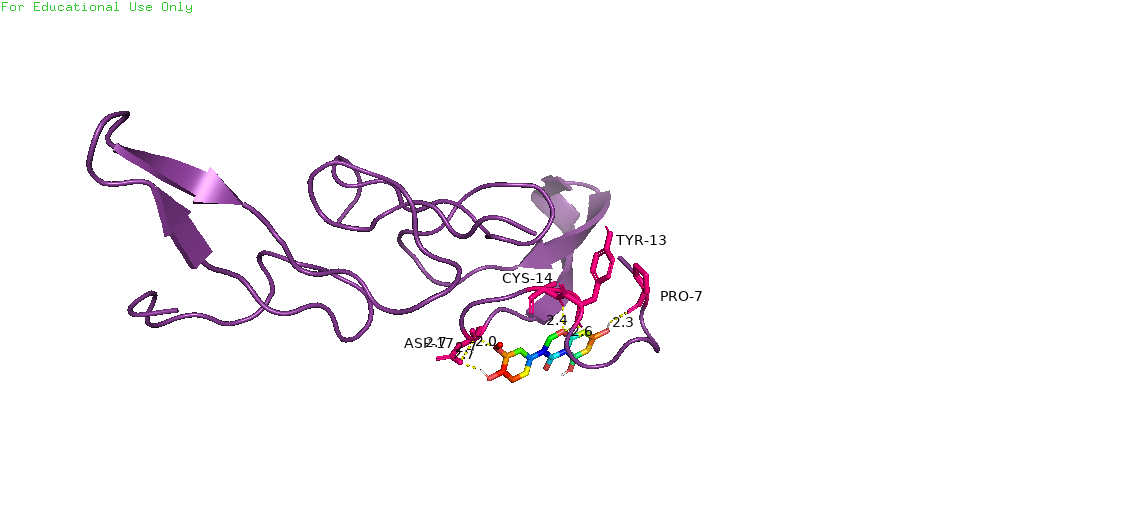


2. EGF---acacetin


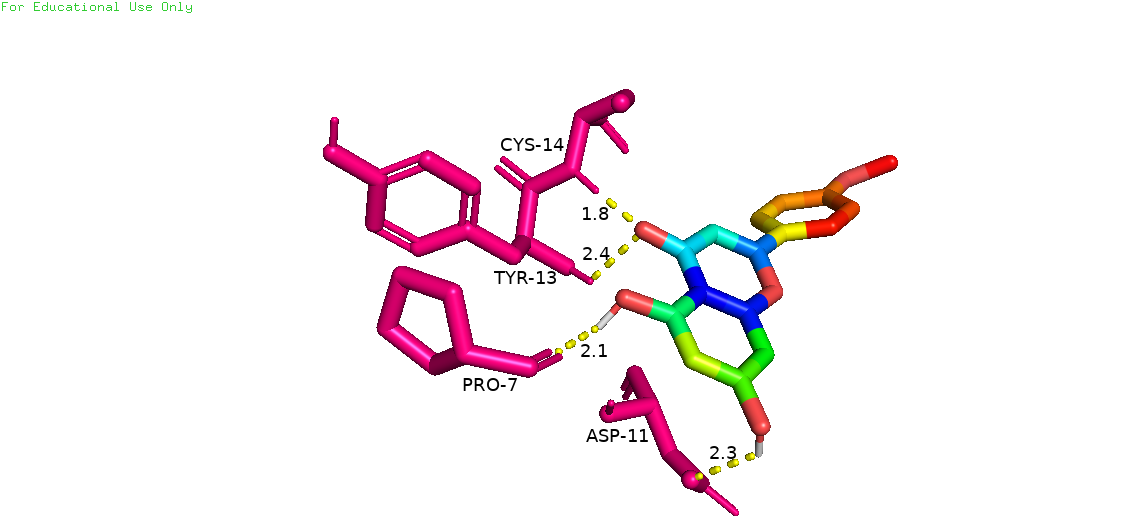

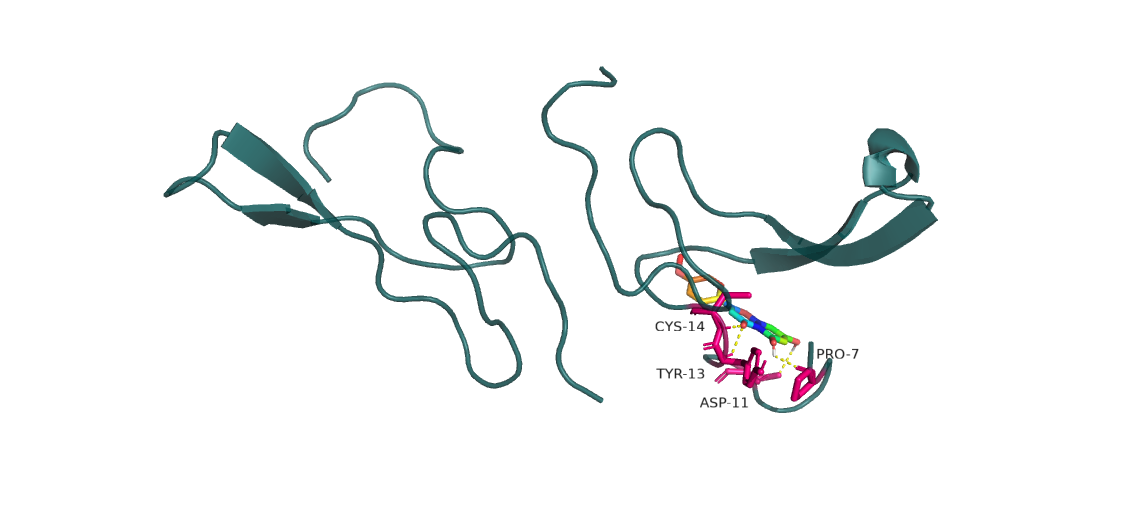


3. EGF---apigenin


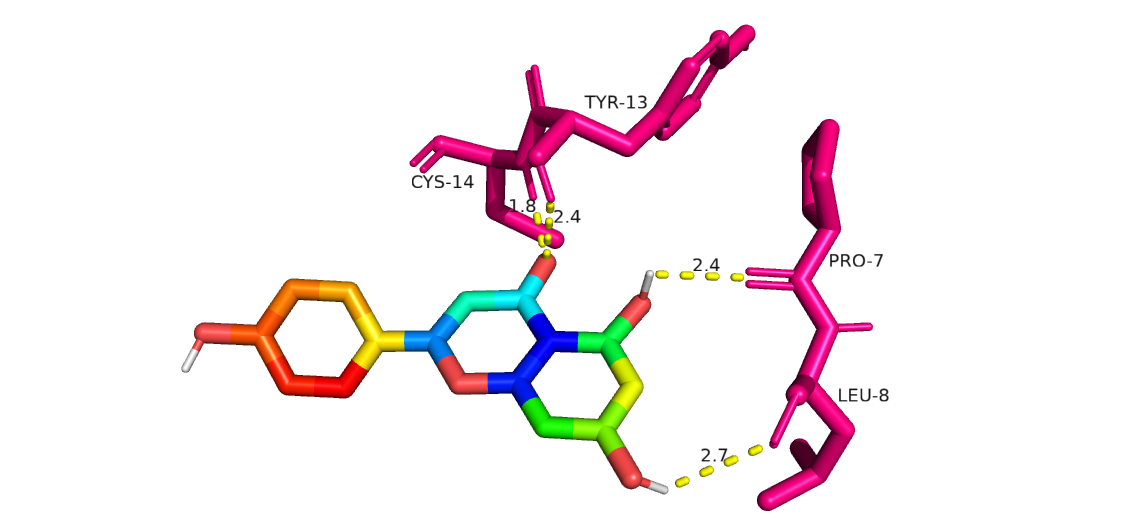

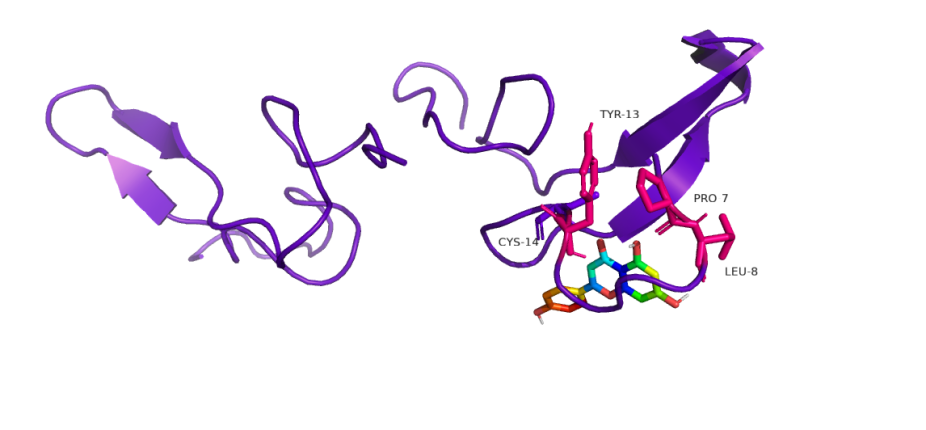


4. EGF---beta-sitosterol

（No hydrogen bond）


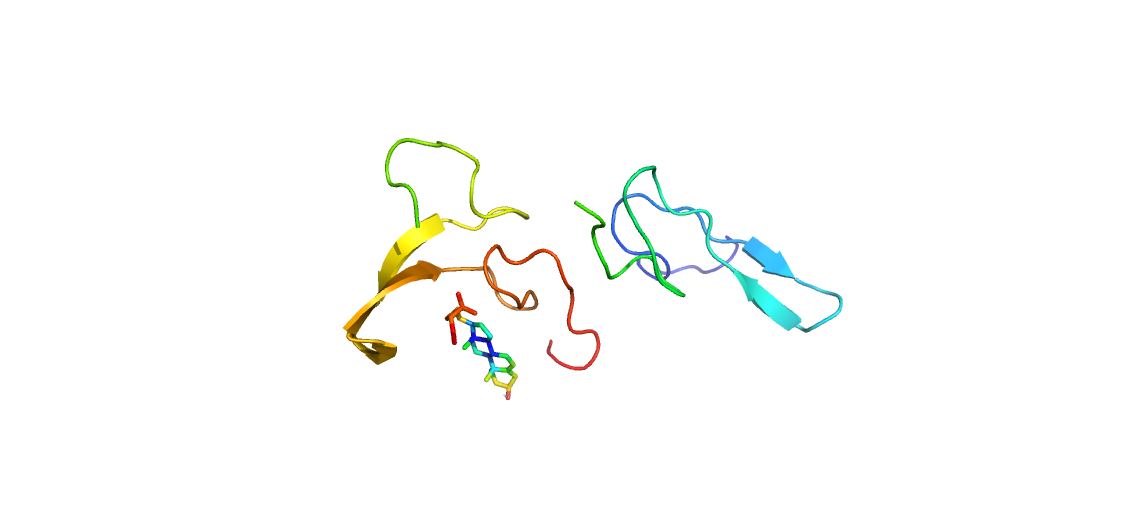


5. EGF---daidzein


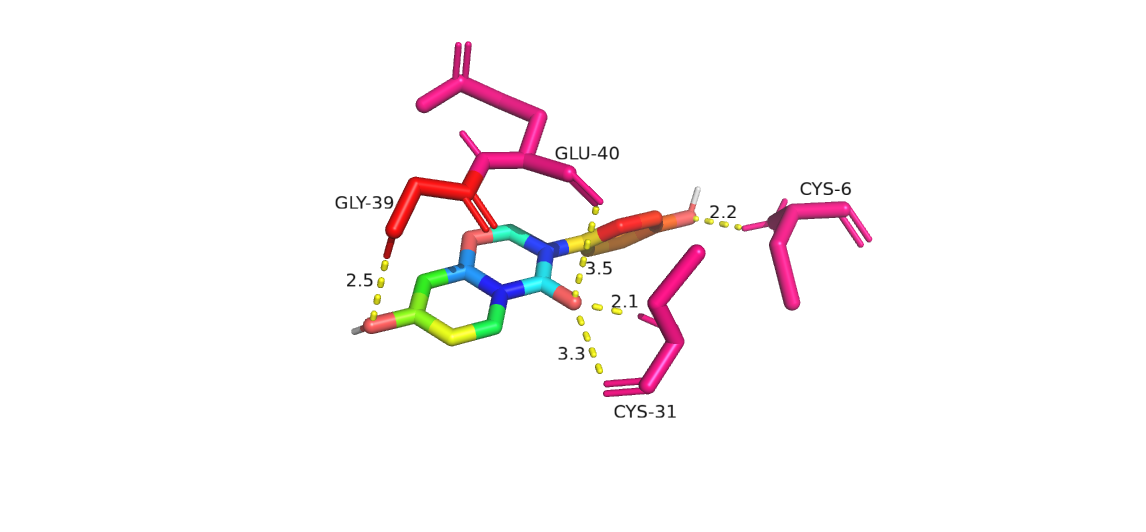

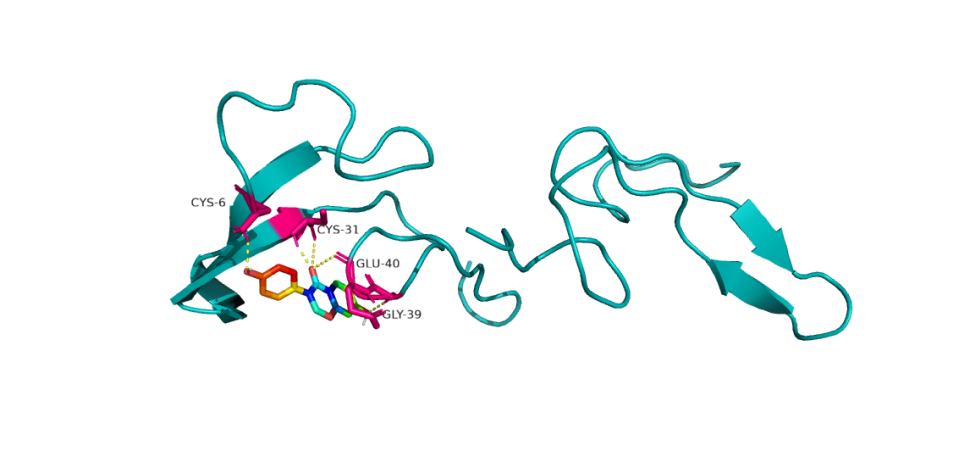


6. EGF---DBP


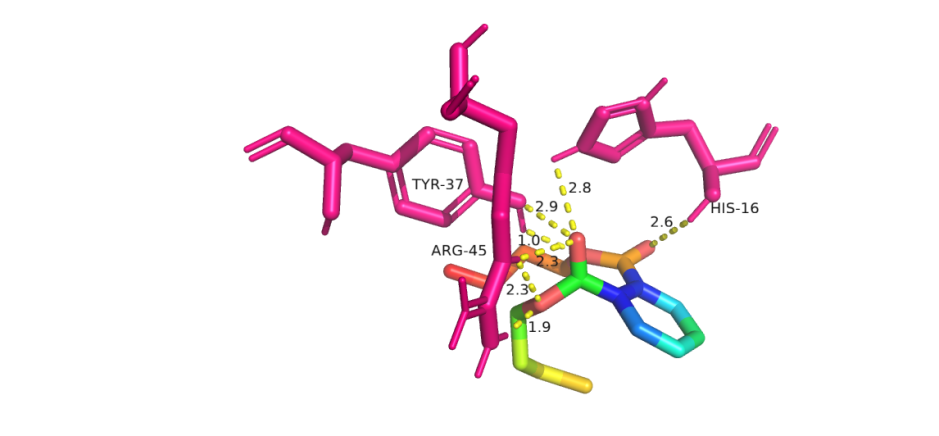

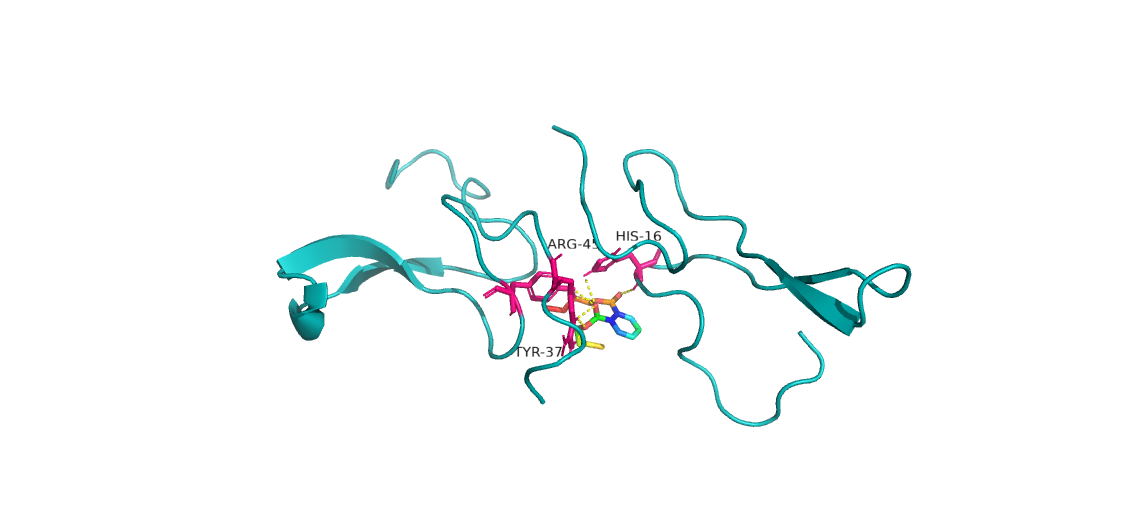


7. EGF---demethylwedelolactone


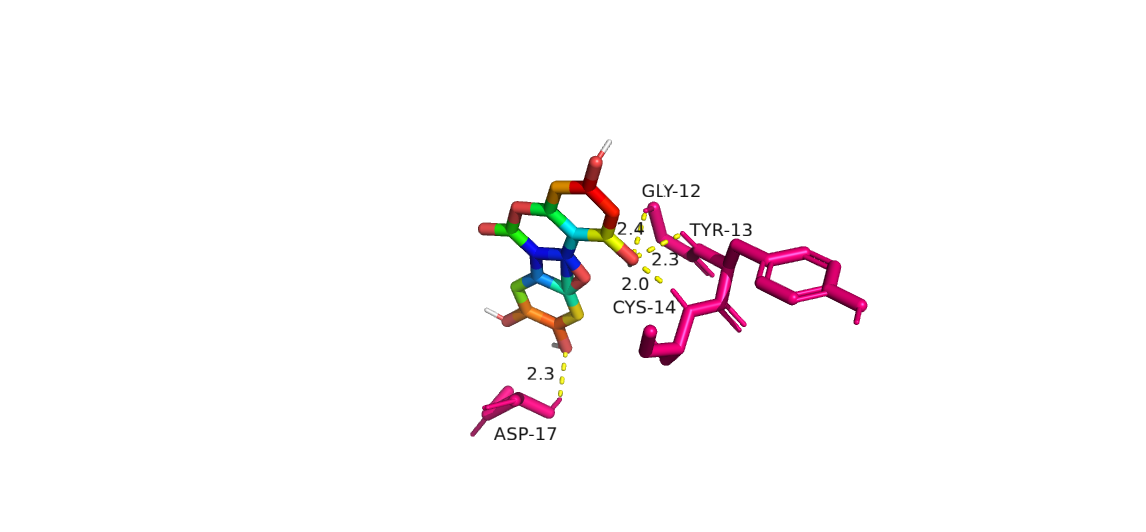

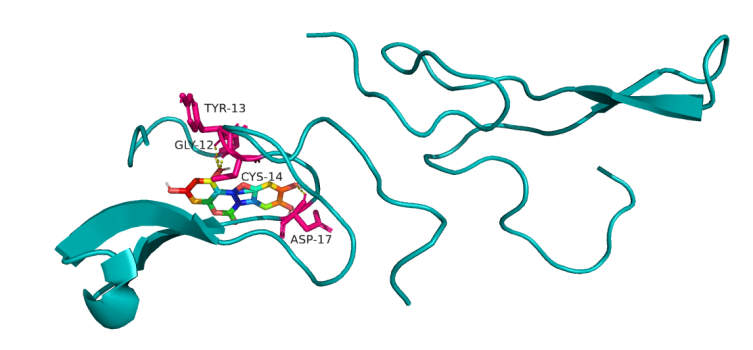


8. EGF---kaempferol


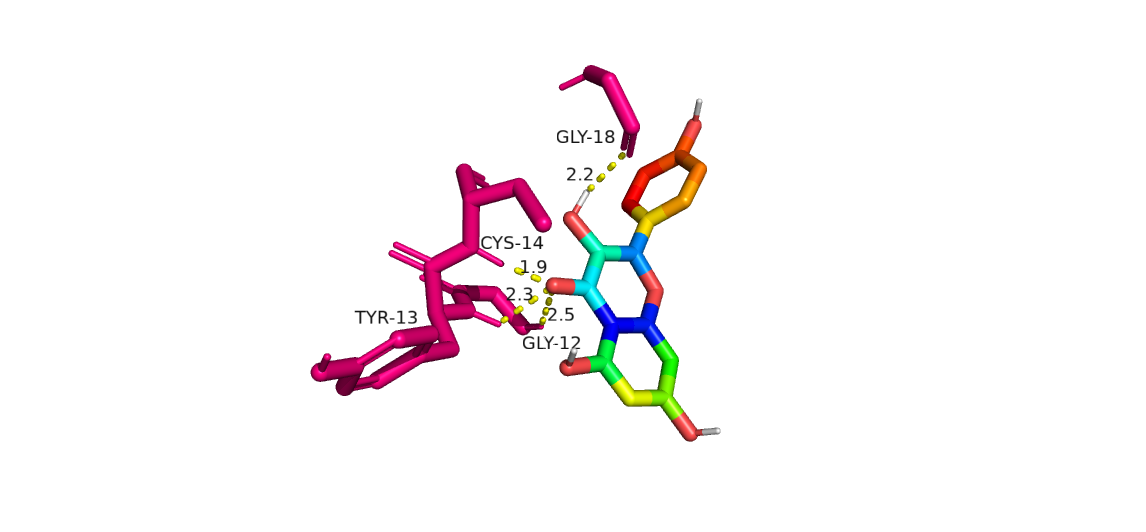

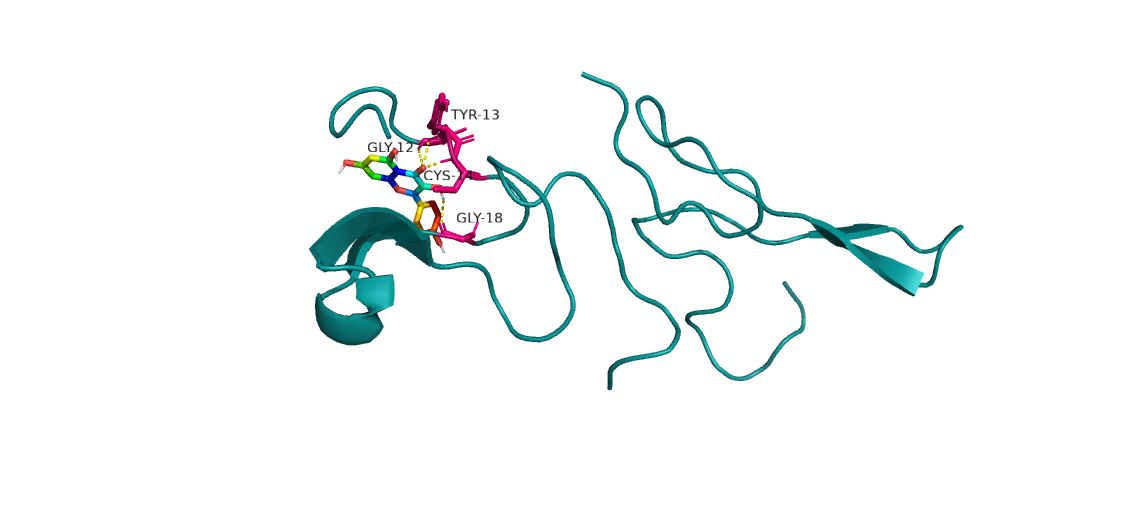


9. EGF---LucidumosideD_qt


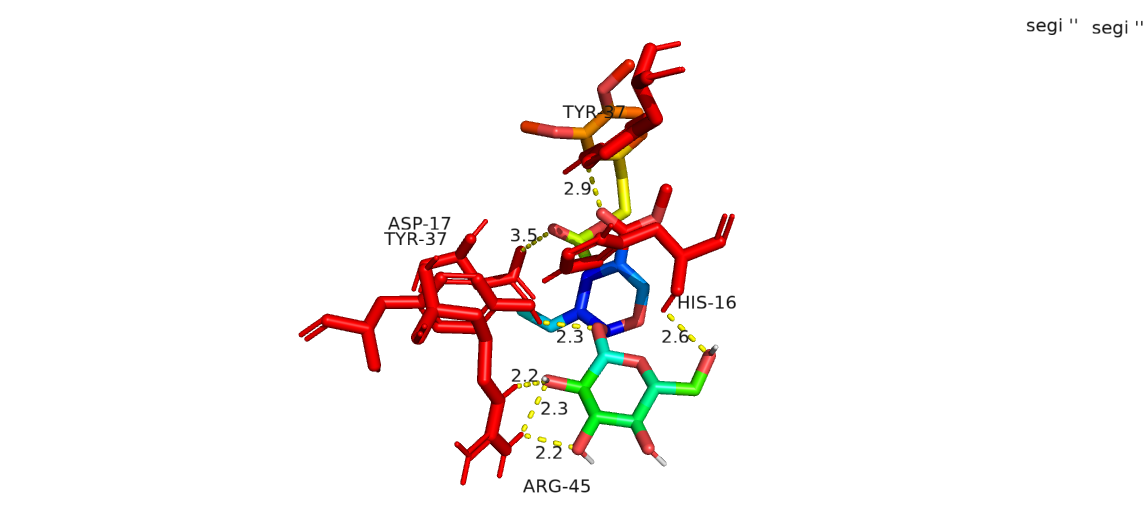

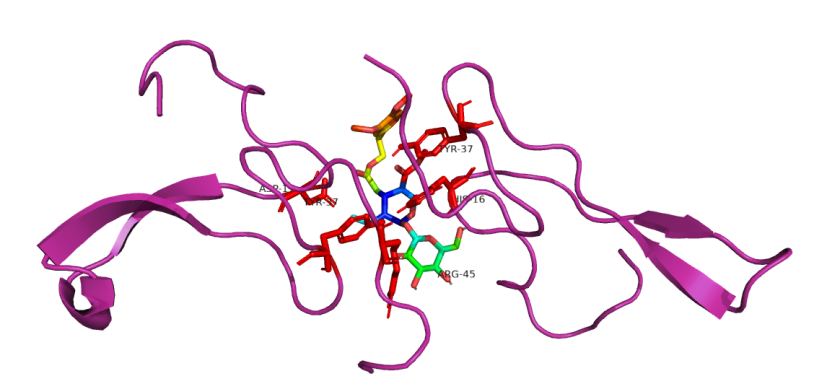


10. EGF---luteolin


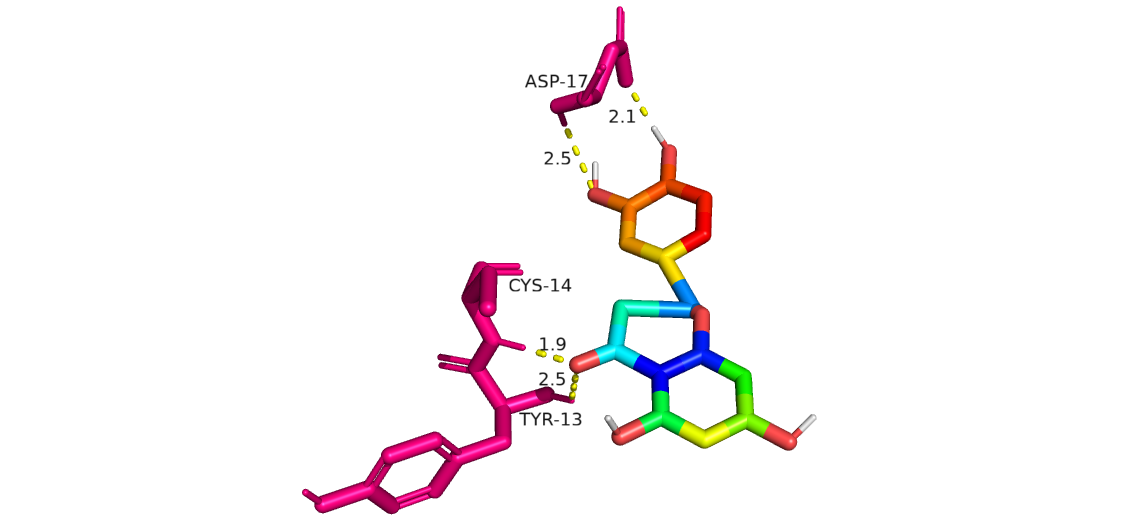

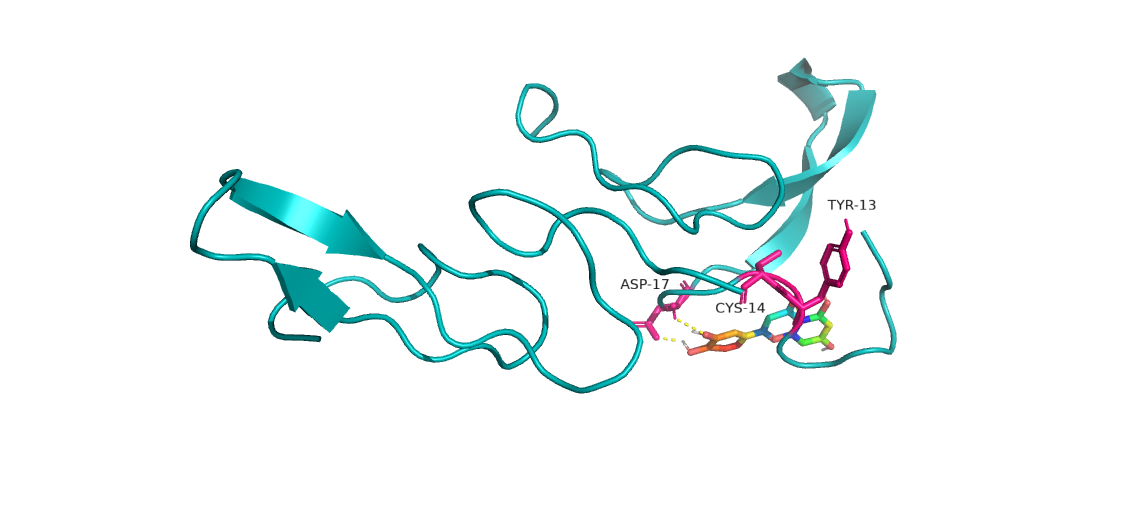


11. EGF---Oleoside-dimethyl ester-qt


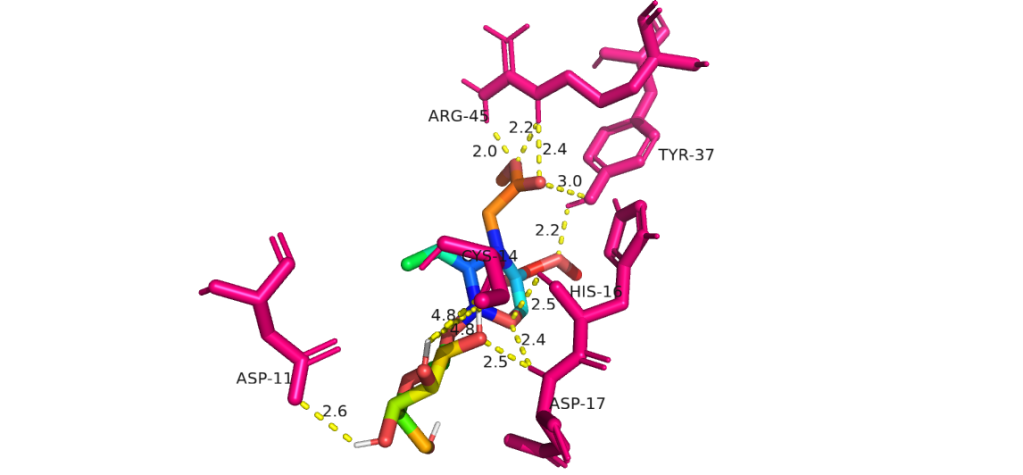

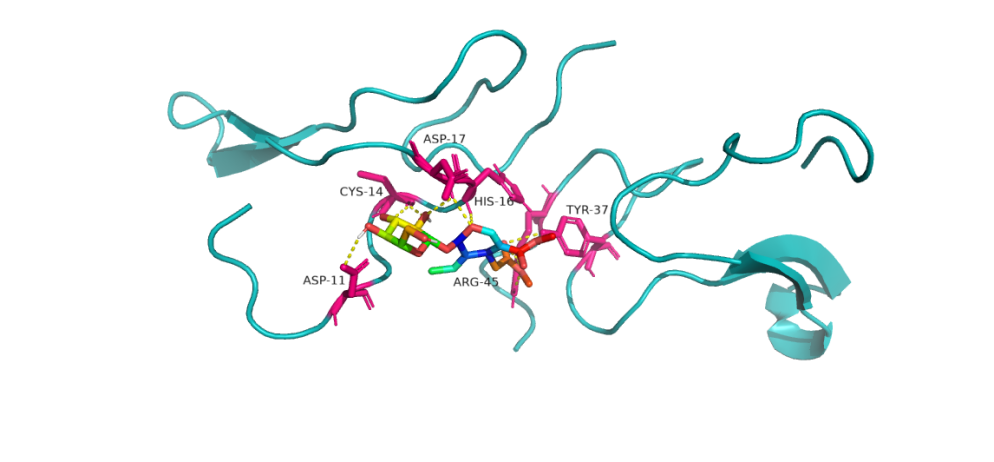


12. EGF---Pratensein


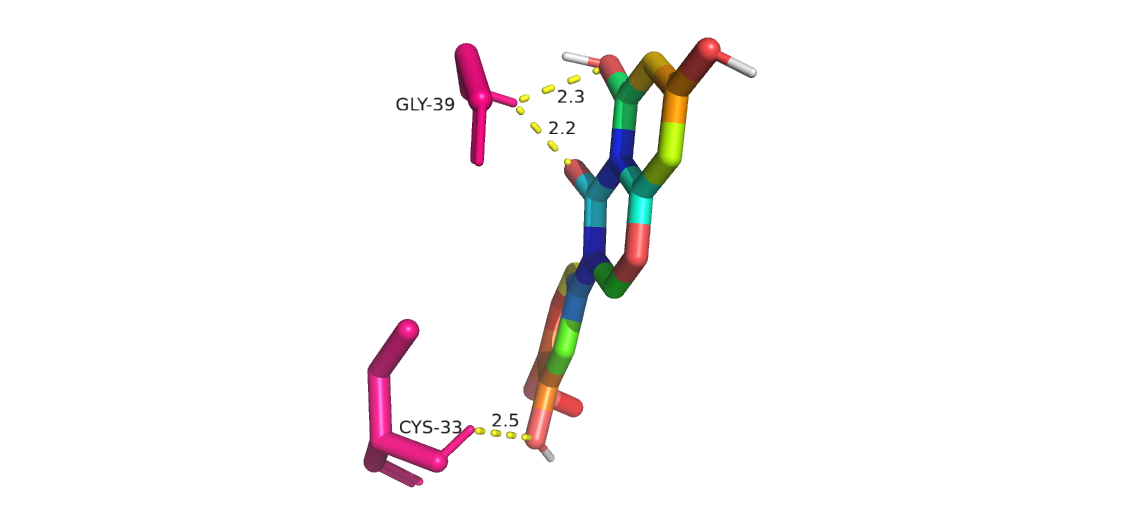

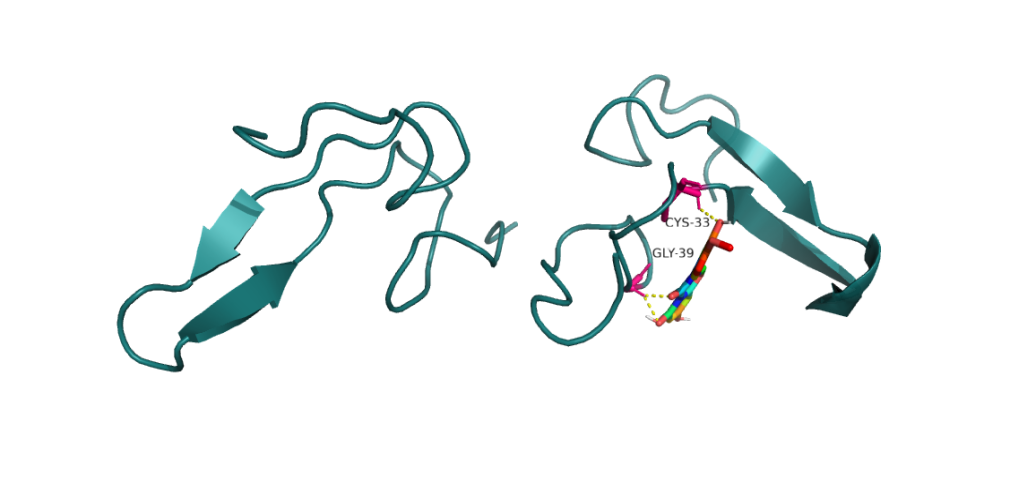


13. EGF---quercetin


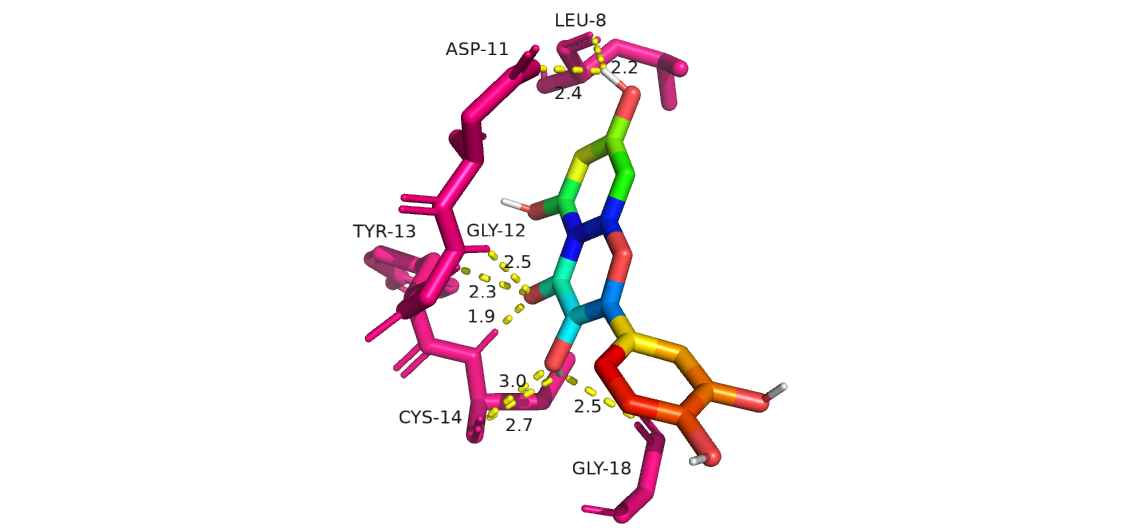

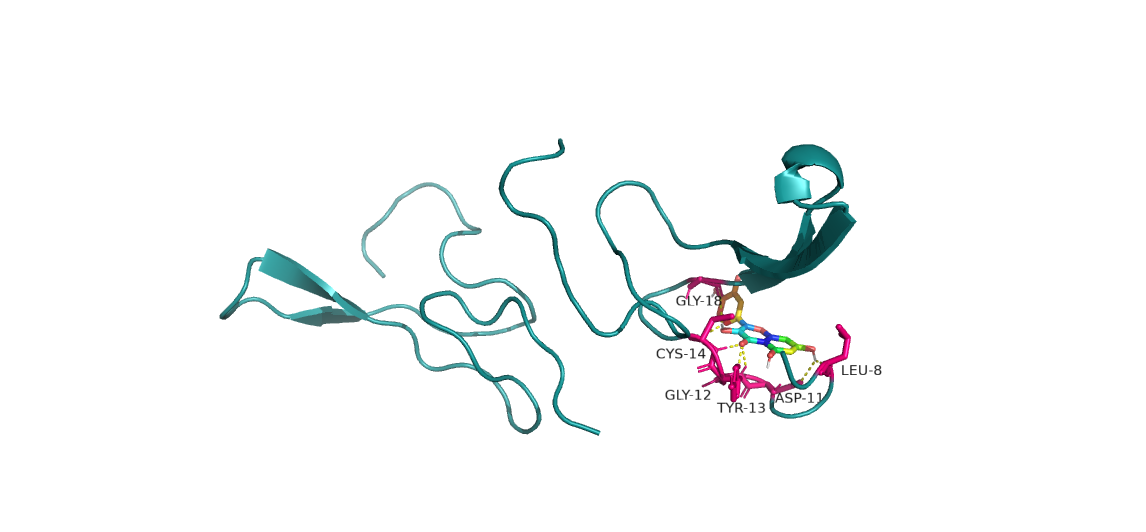


14. EGF---salidroside


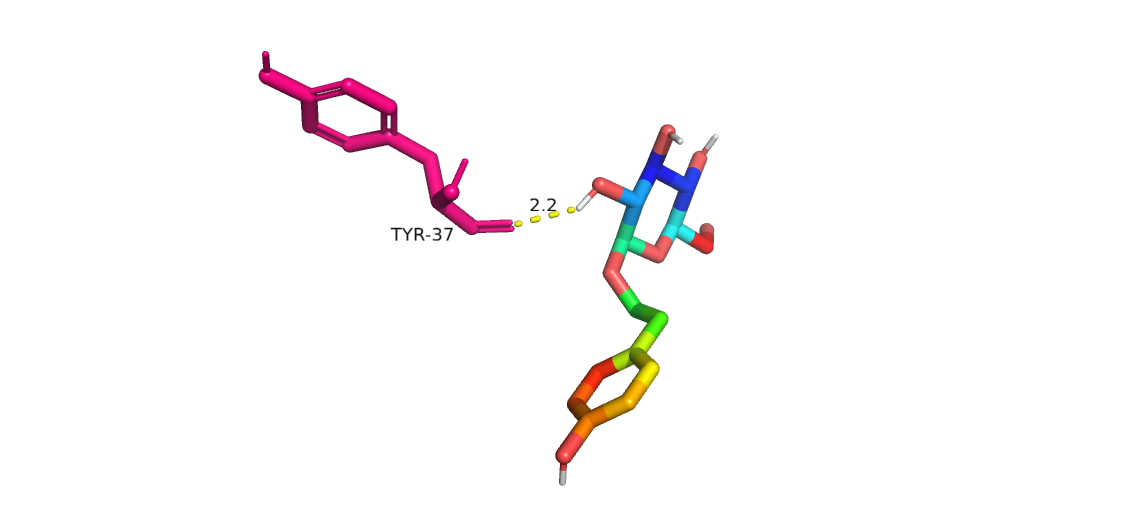

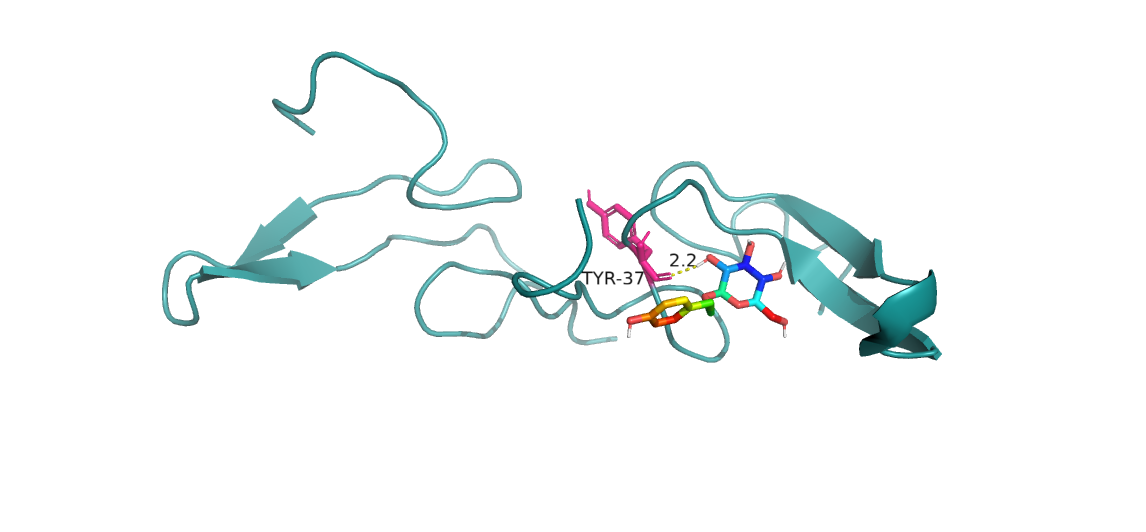


15. EGF---Specnuezhenide


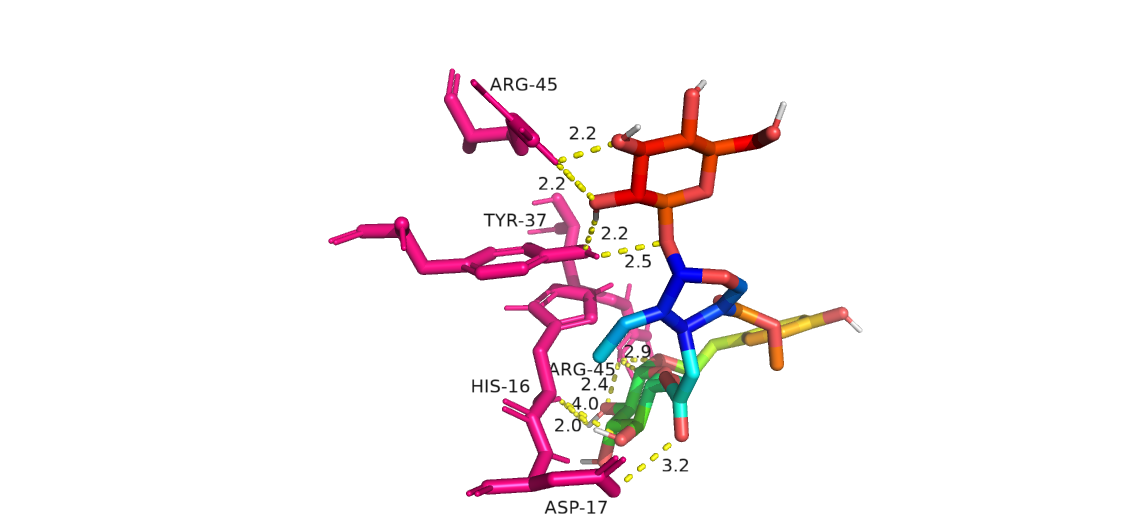

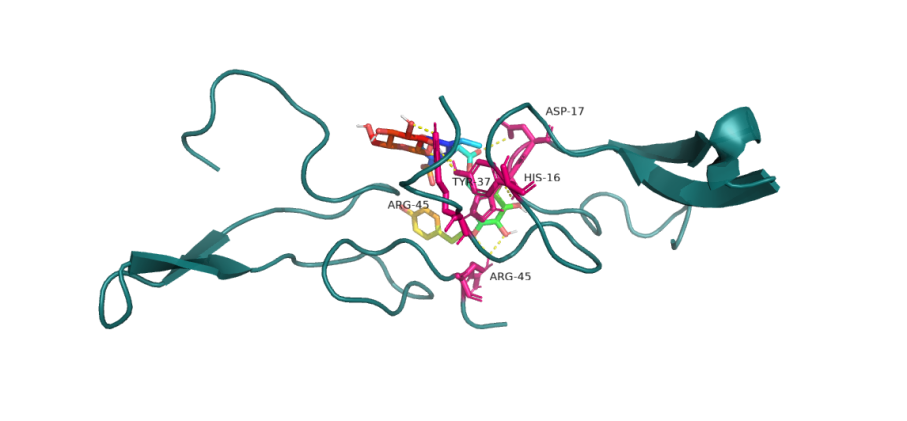


16. EGF---ursolic-acid


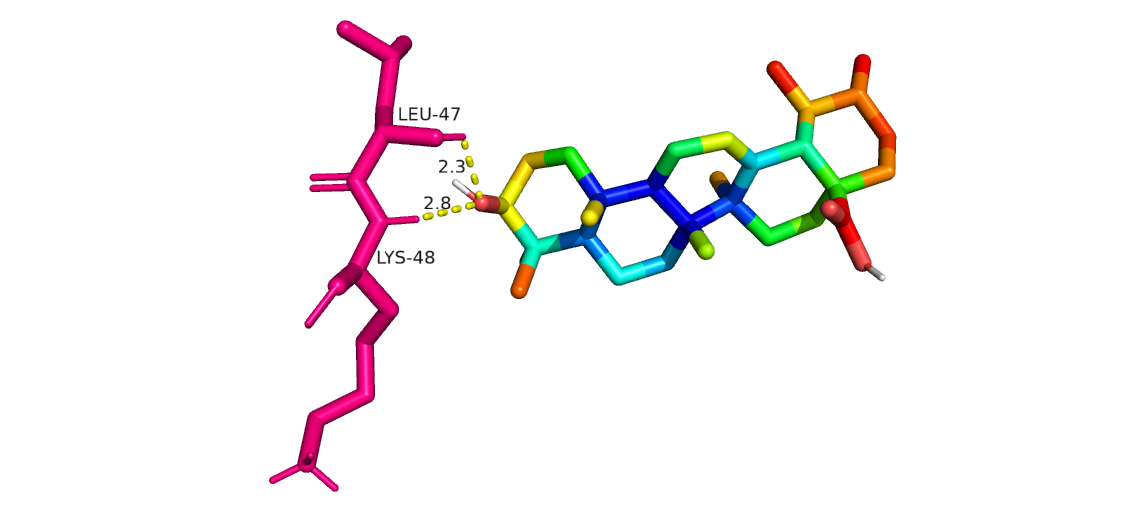

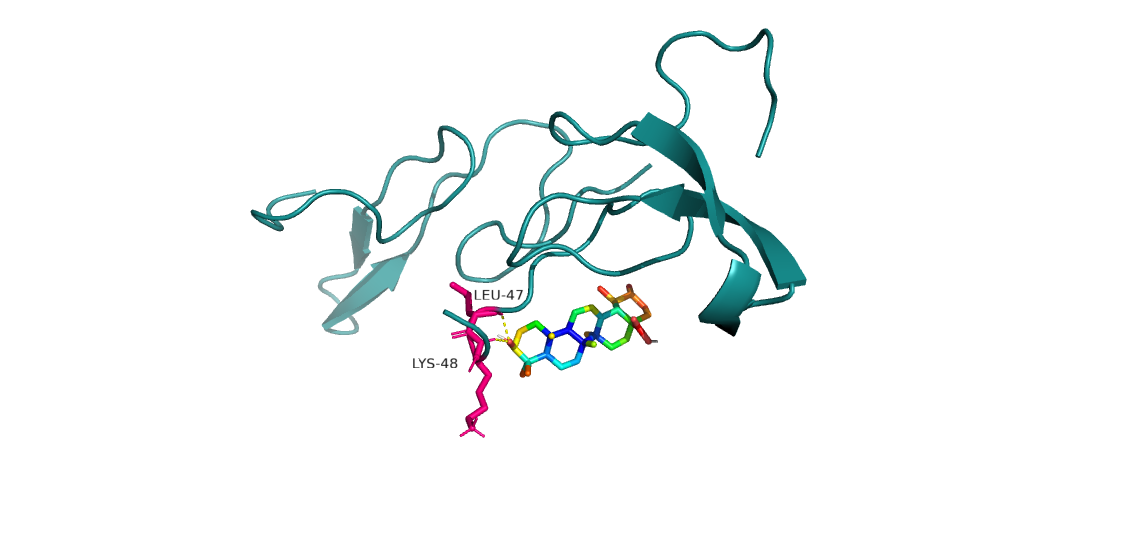


17. EGF---wedelolactone


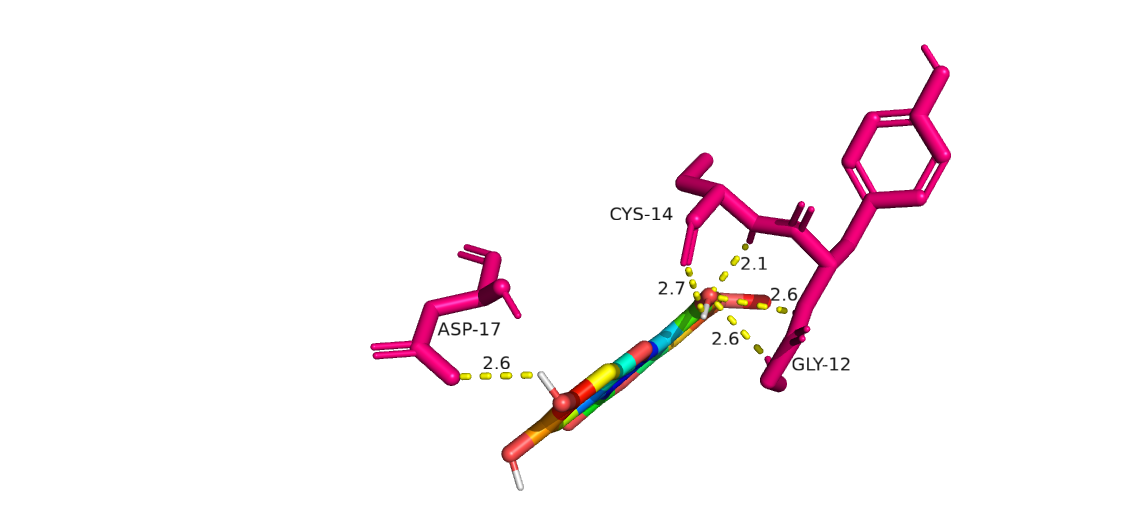

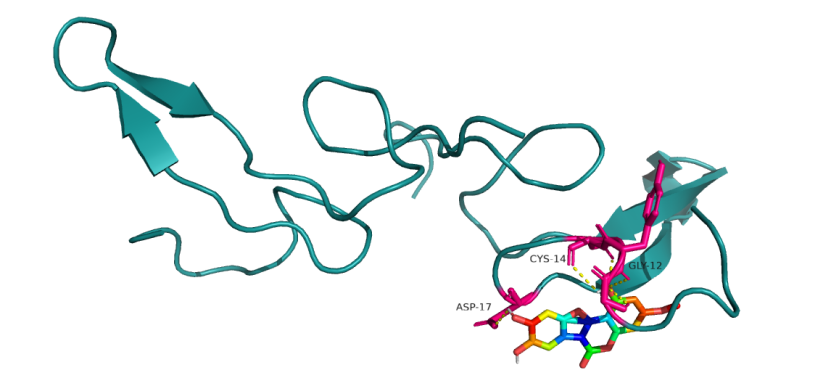


18. IL-2---3'-O-Methylorobol


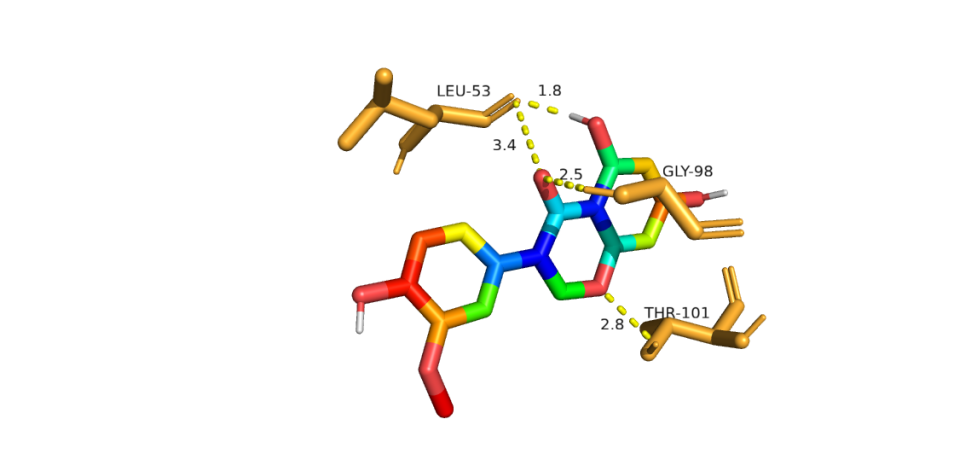

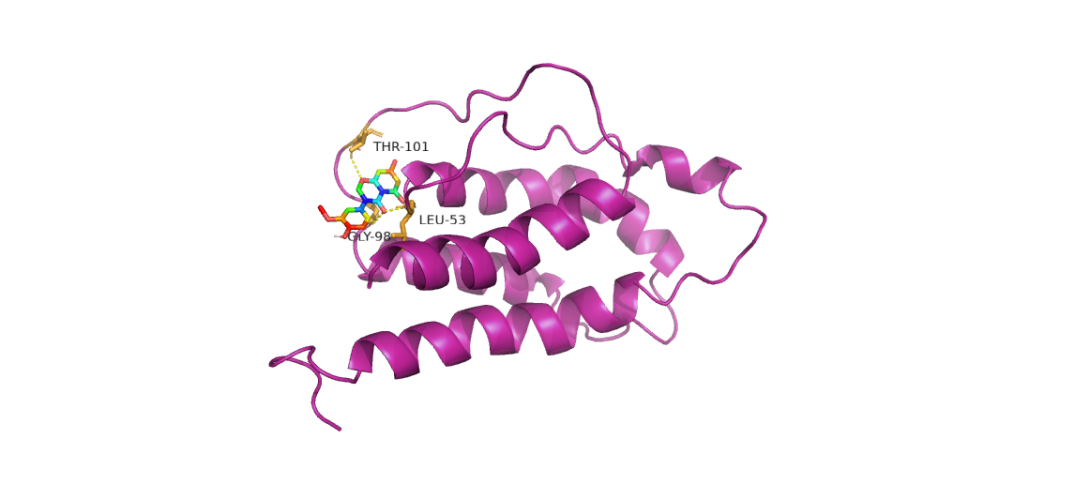


19. IL-2---acacetin


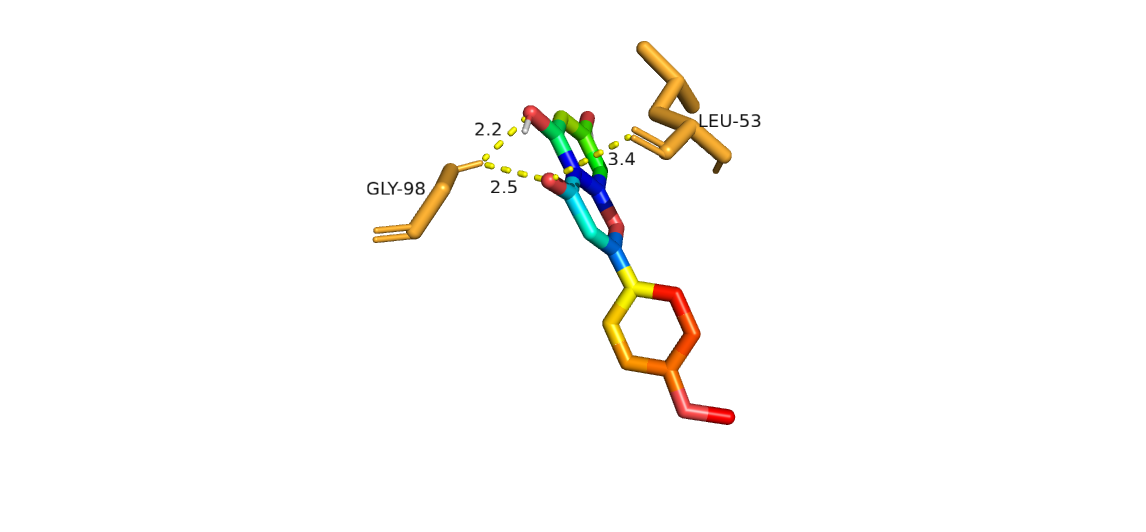

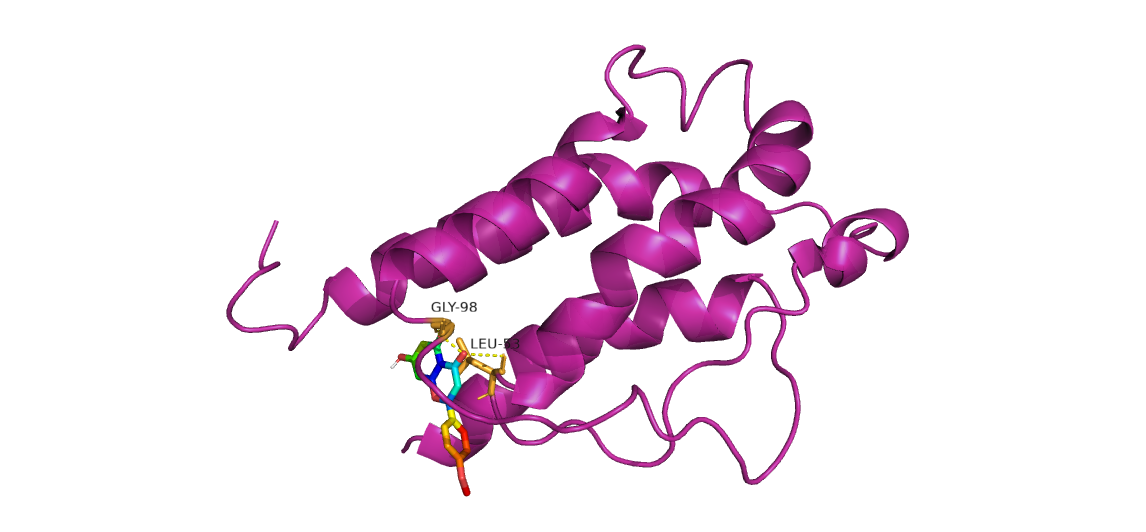


20. IL-2---apigenin


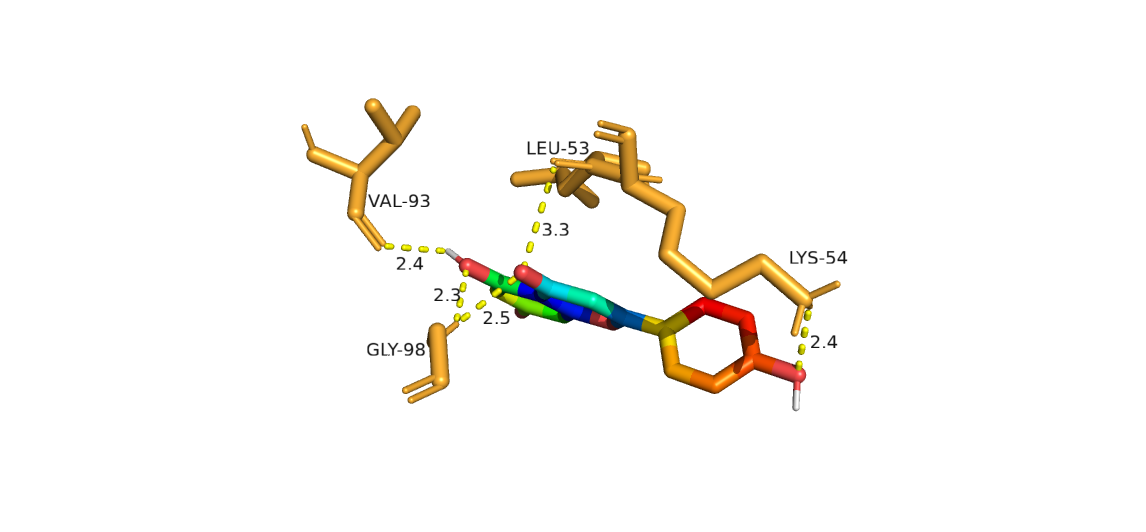

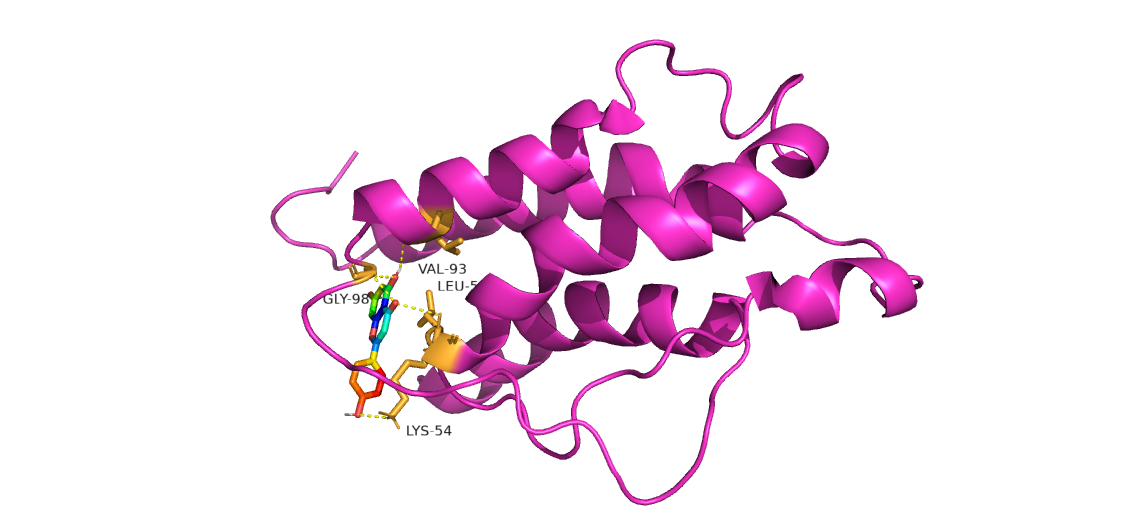


21. IL-2---beta-sitosterol


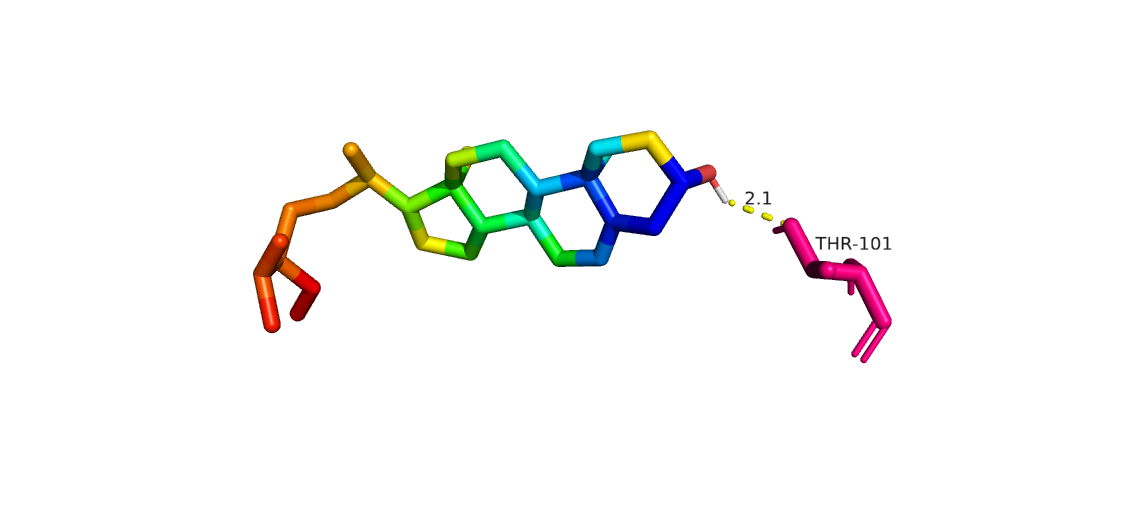

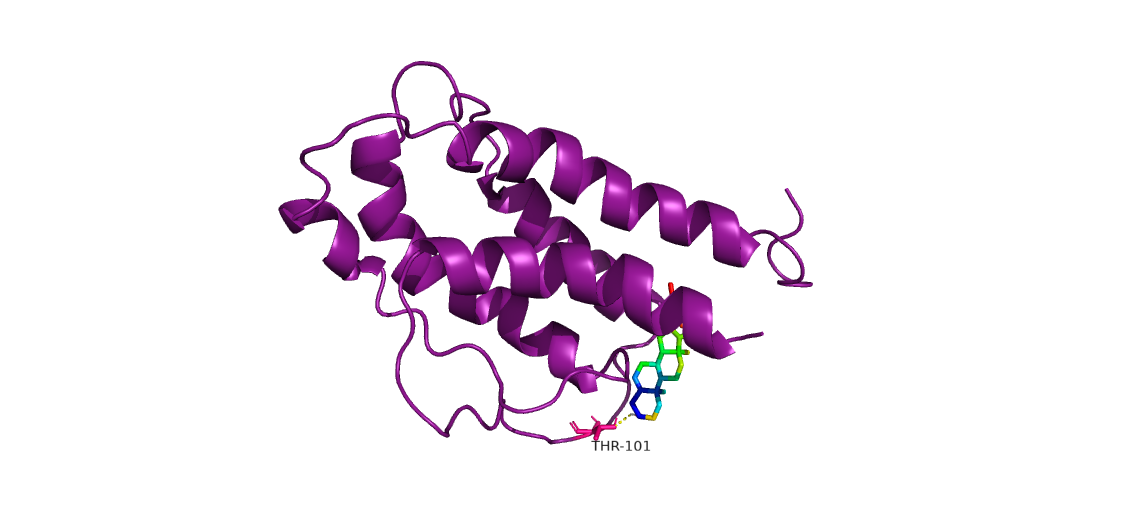


22. IL-2---daidzein


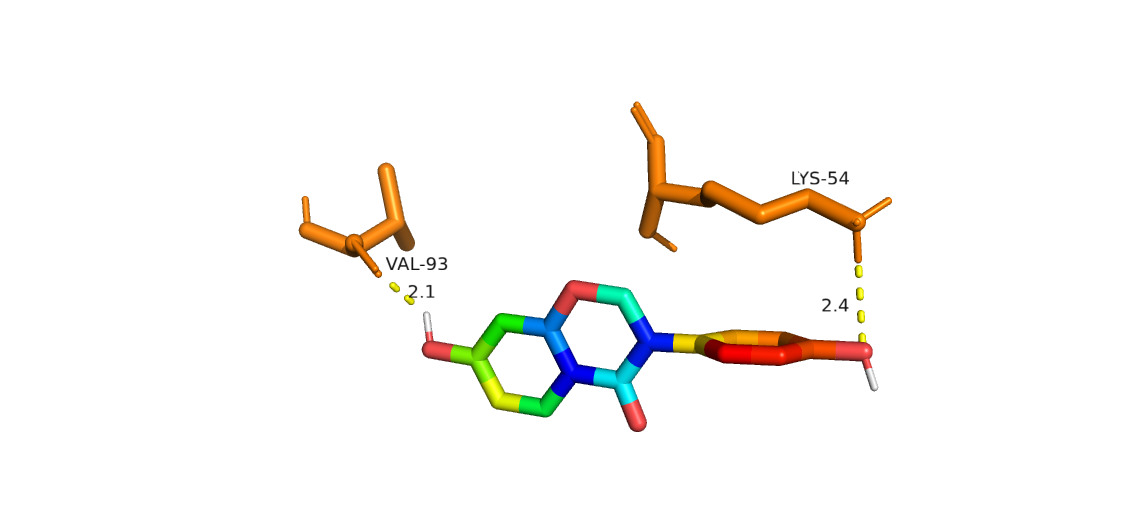

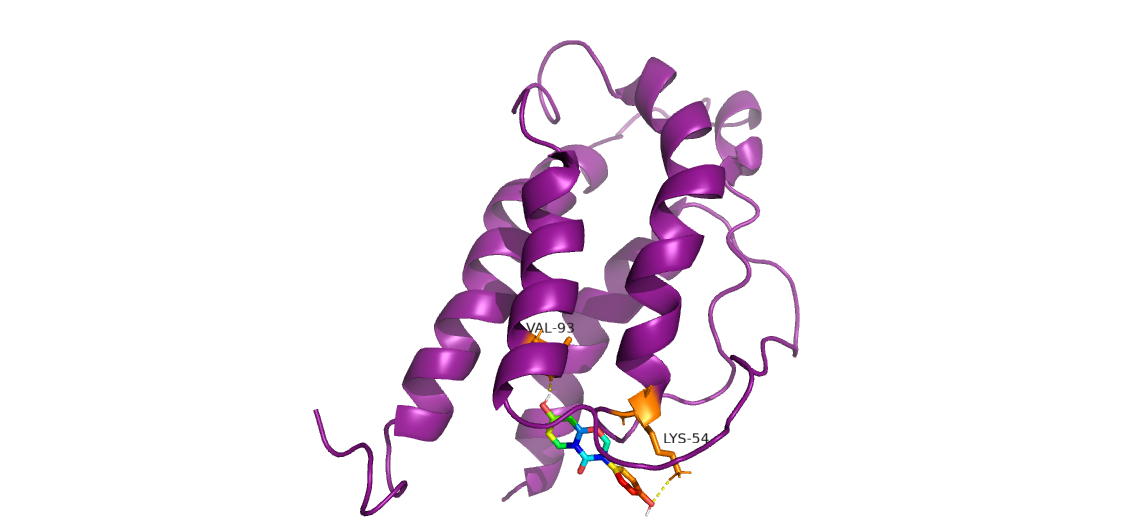


23. IL-2---DBP


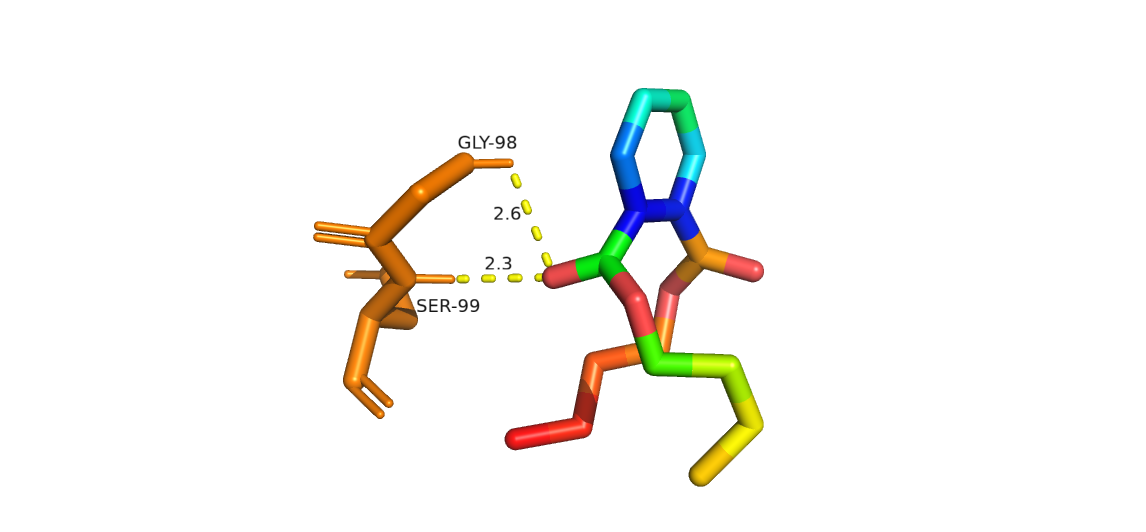

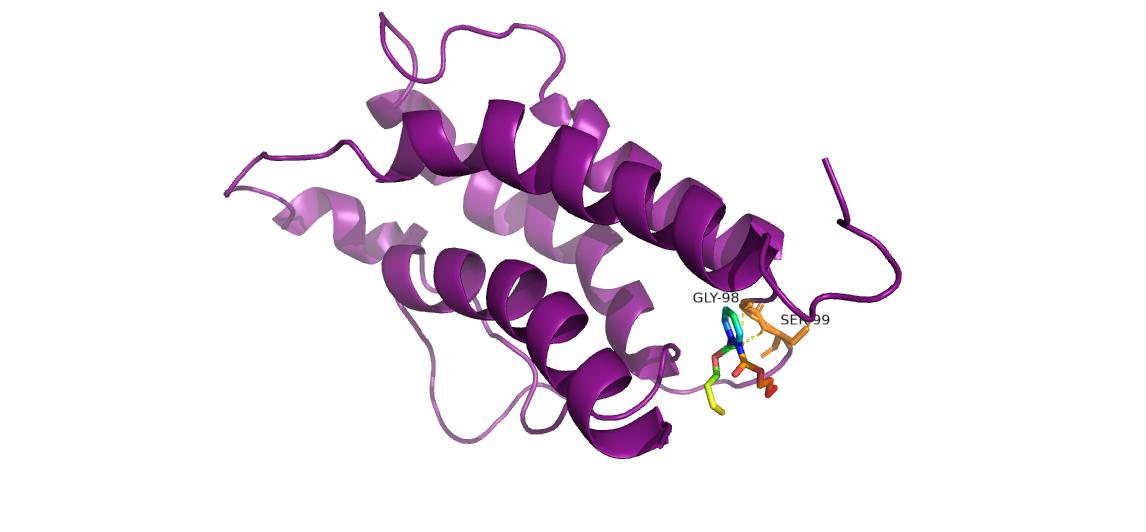


24. IL-2---demethylwedelolactone


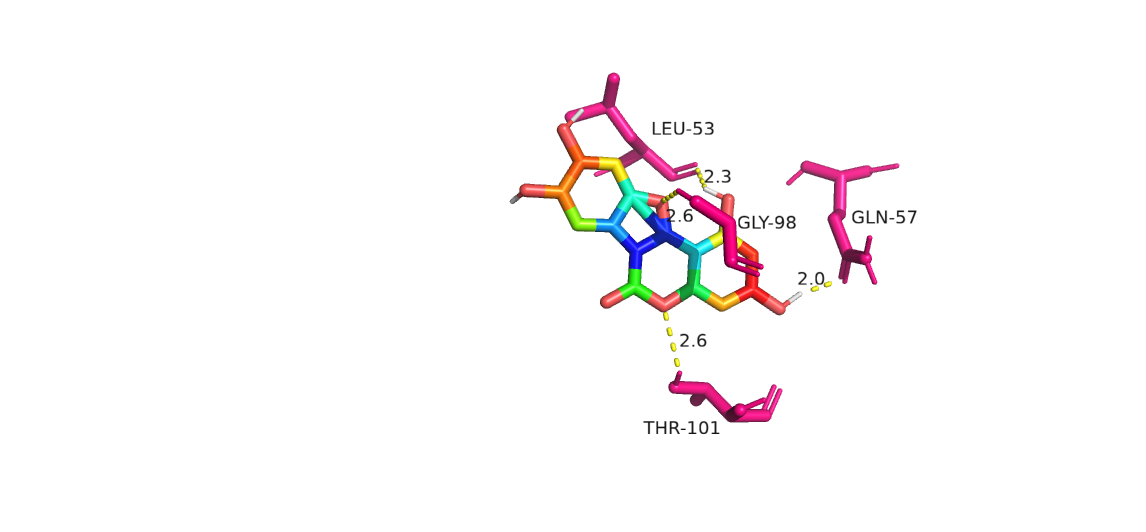

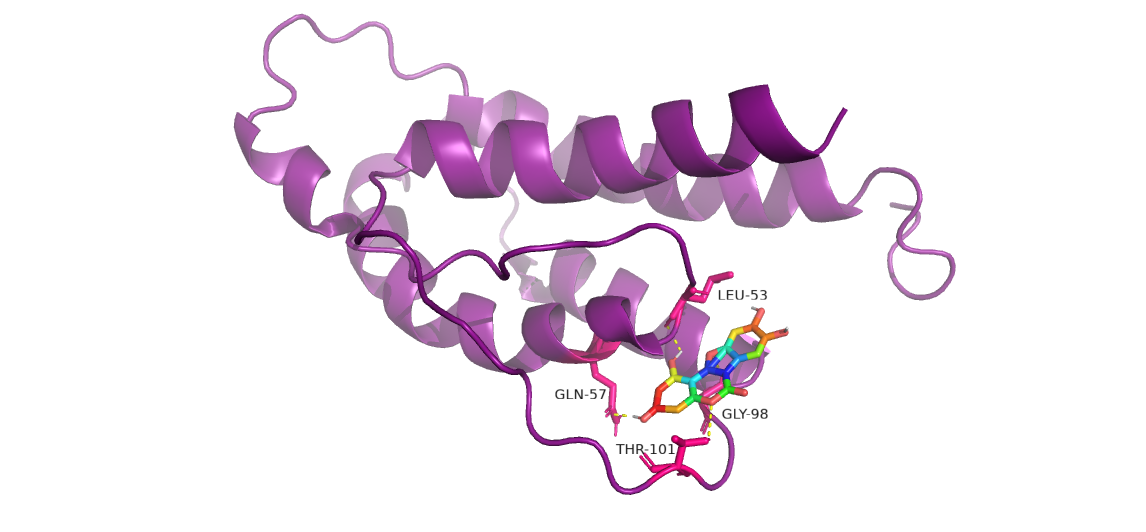


25. IL-2---kaempferol


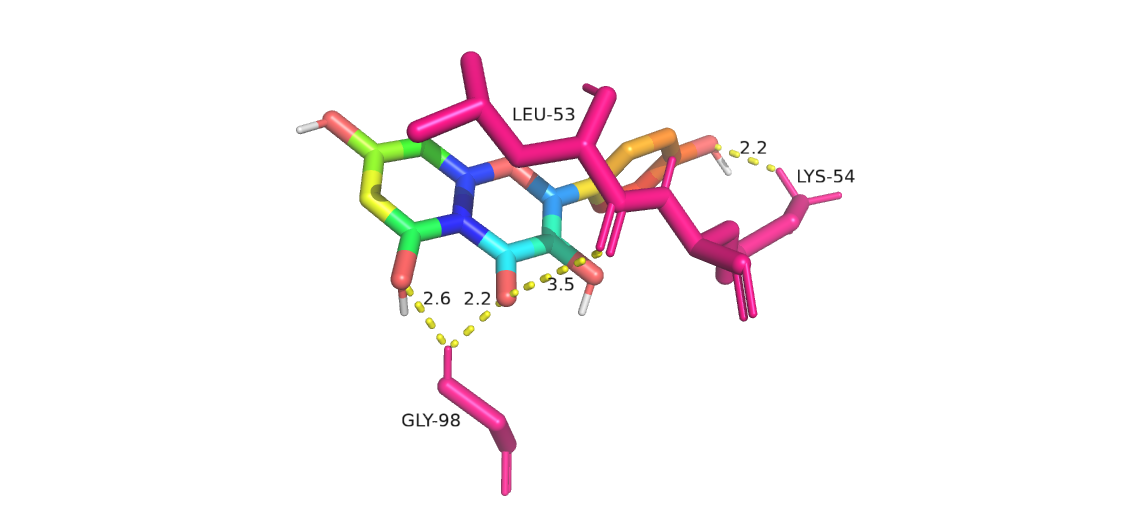

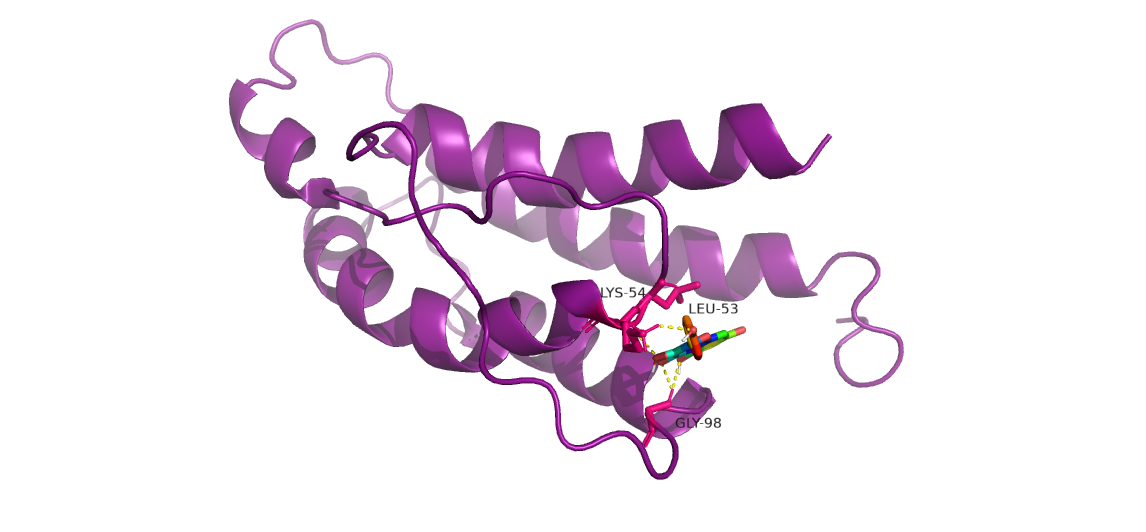


26. IL-2---Lucidumoside D-qt


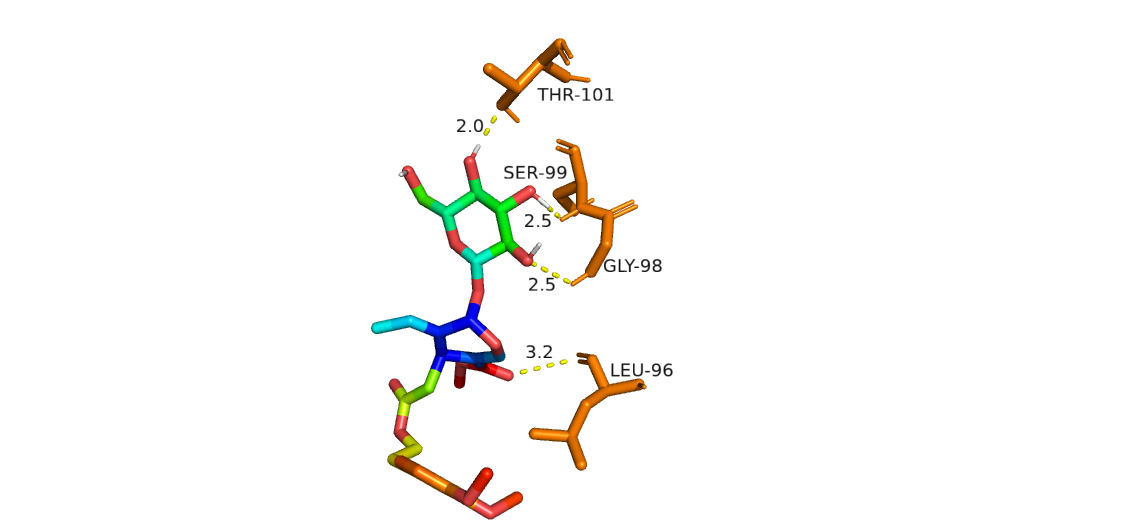

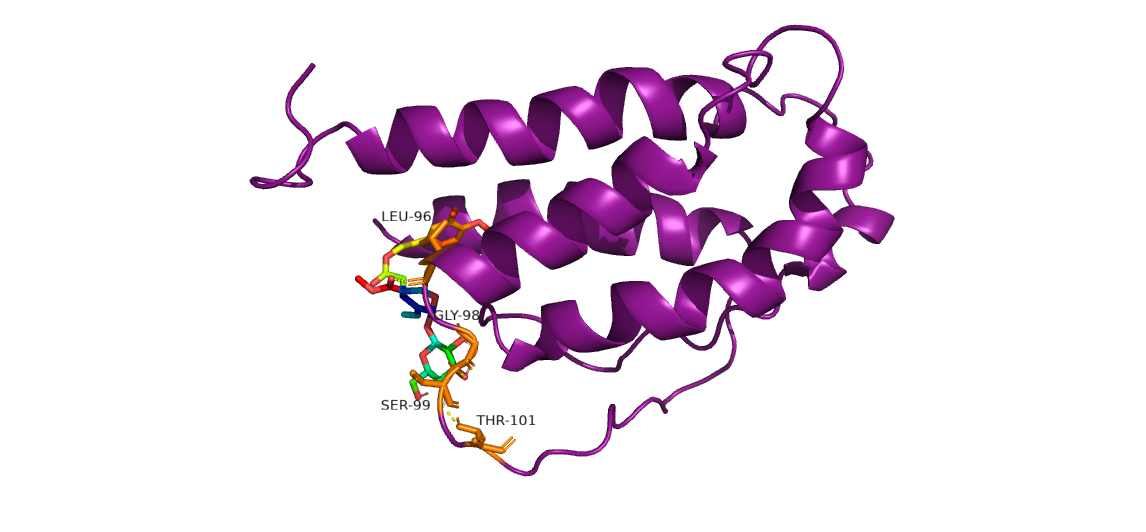


27. IL-2---luteolin


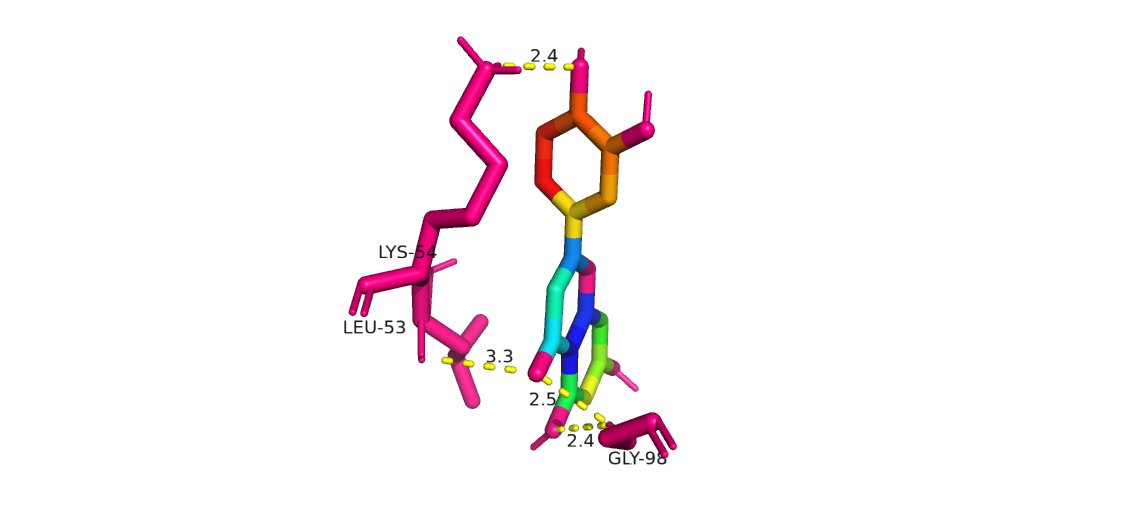

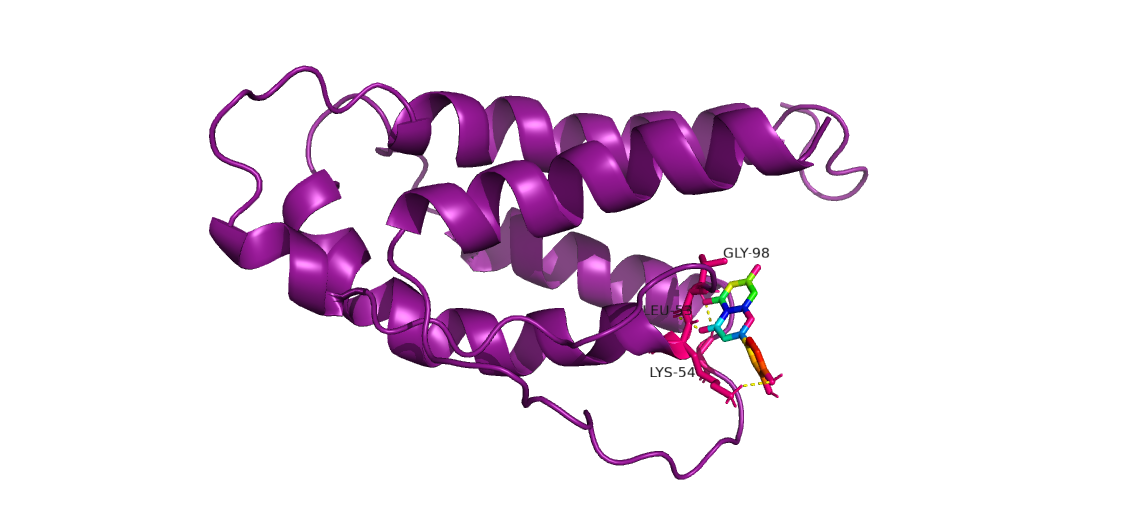


1. IL-2---Oleoside-dimethyl ester-qt


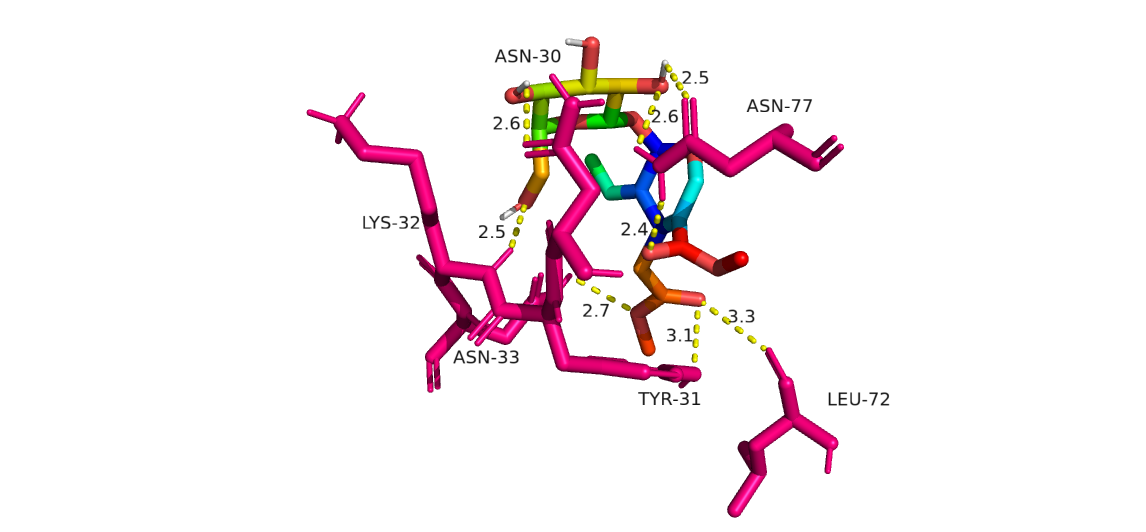

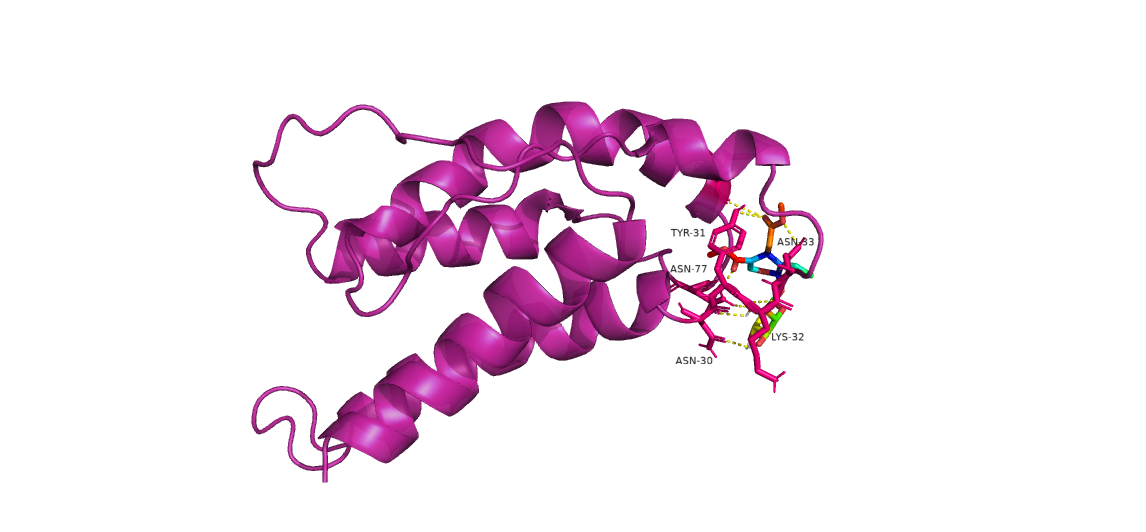


29. IL-2---Pratensein


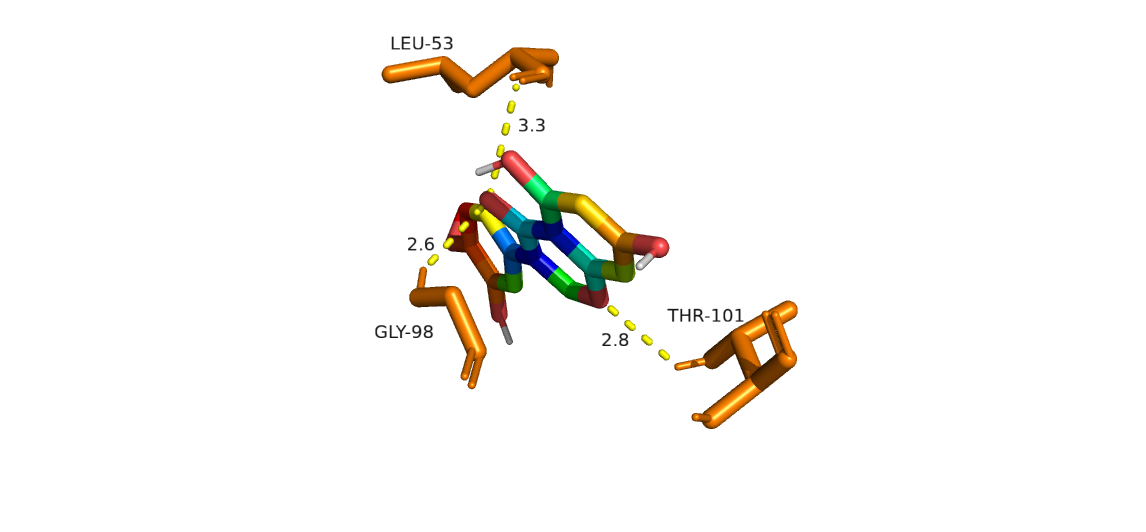

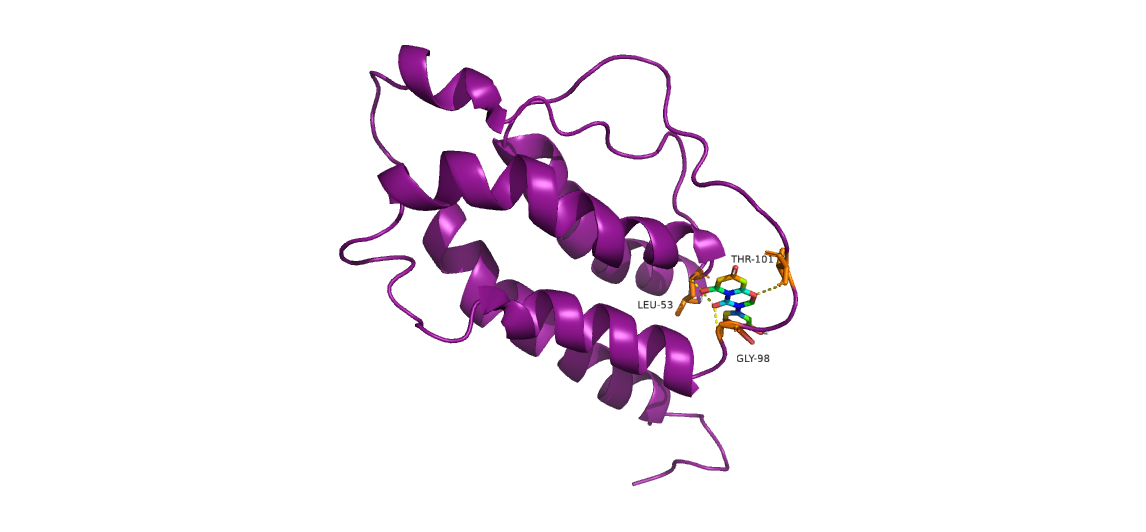


30. IL-2---quercetin


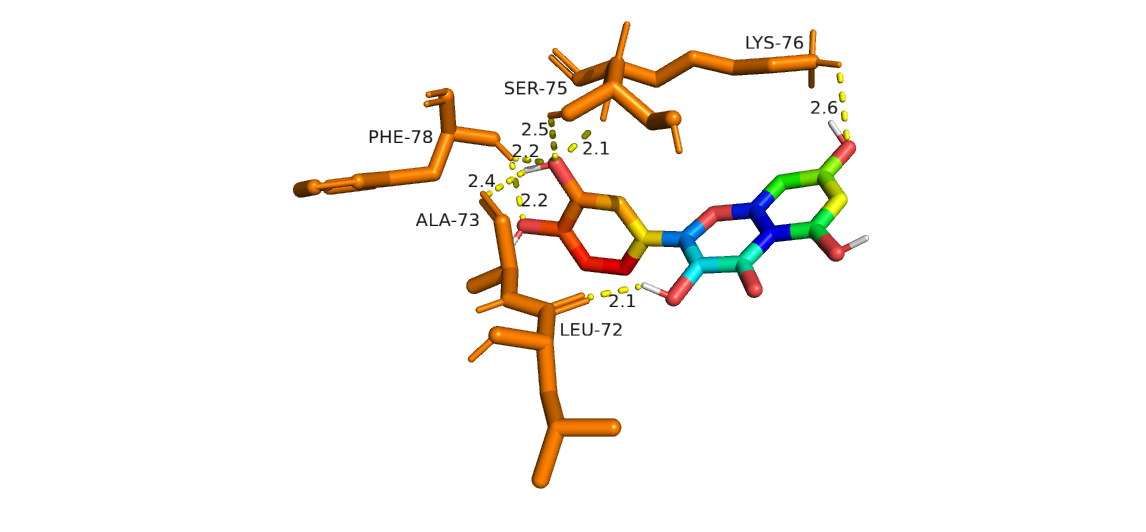

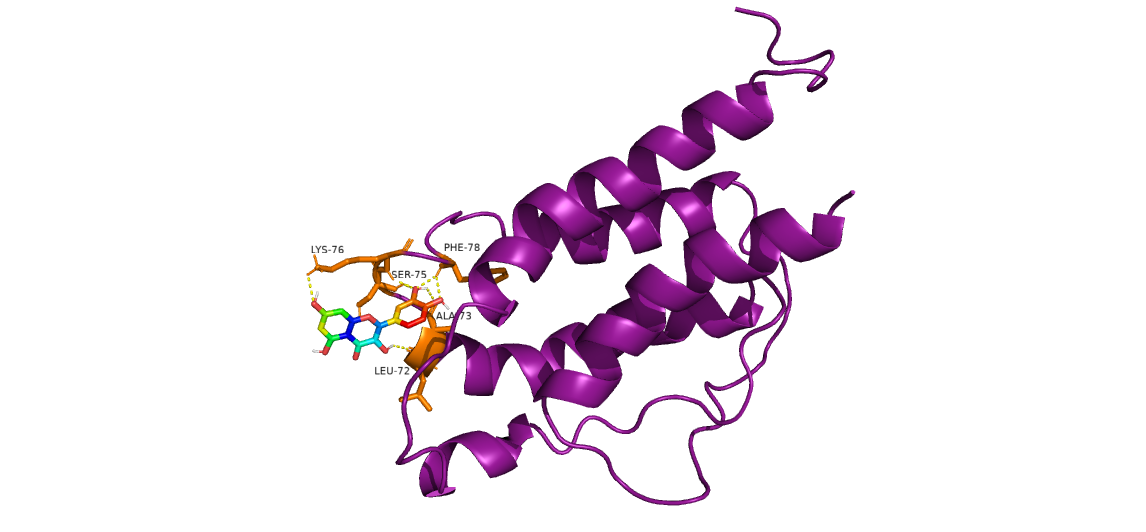


31. IL-2---salidroside


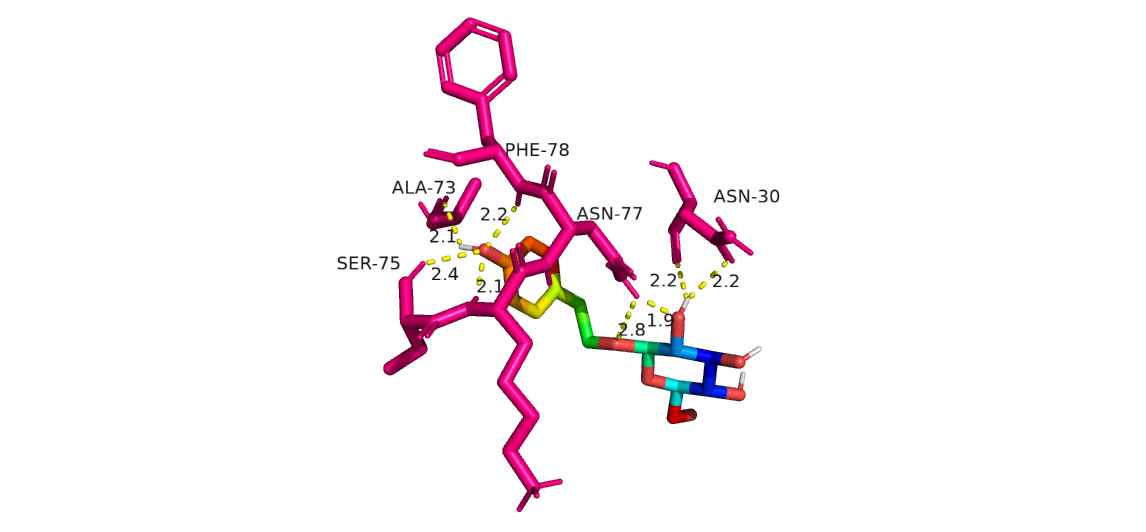

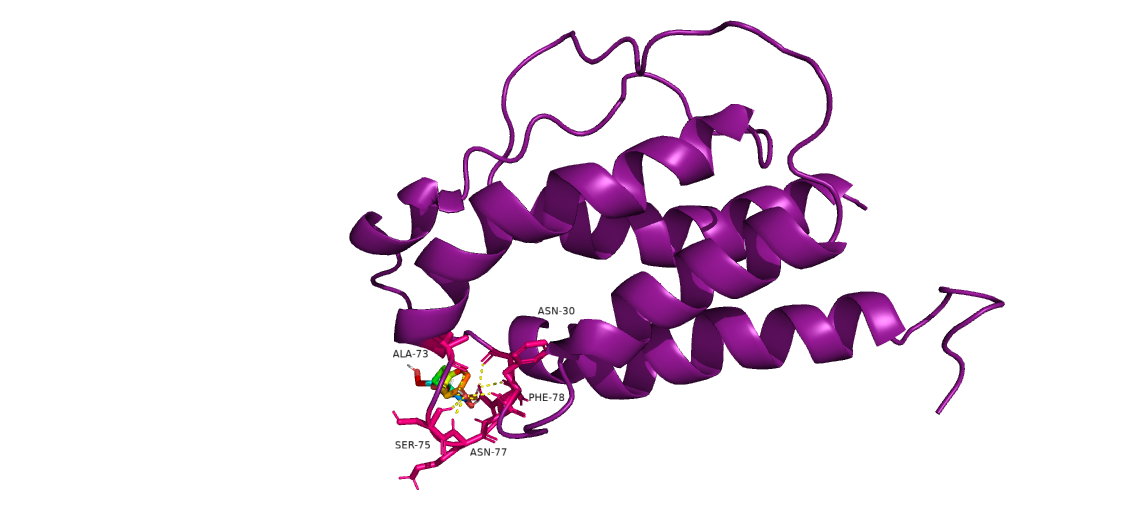


32. IL-2---Specnuezhenide


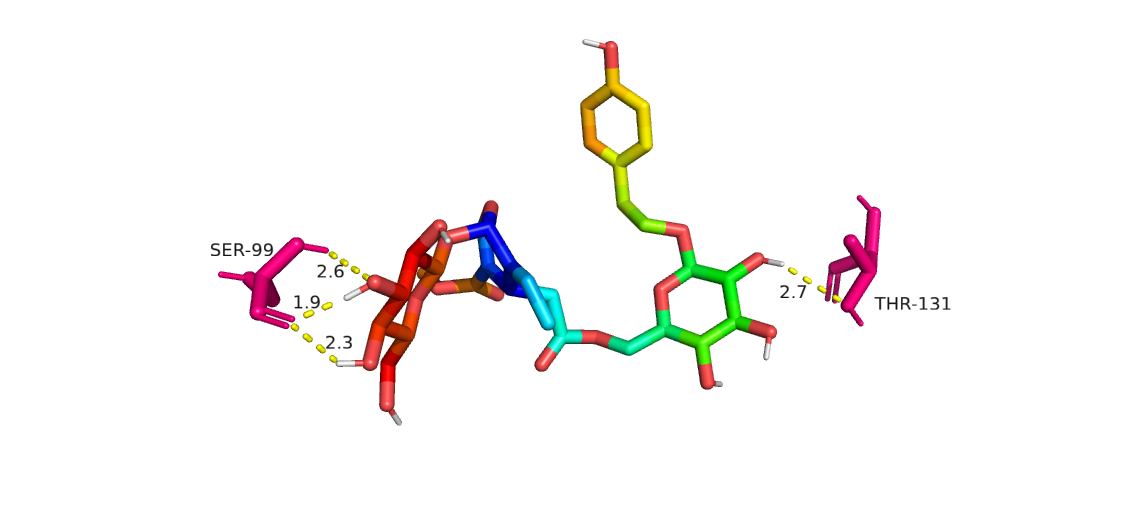

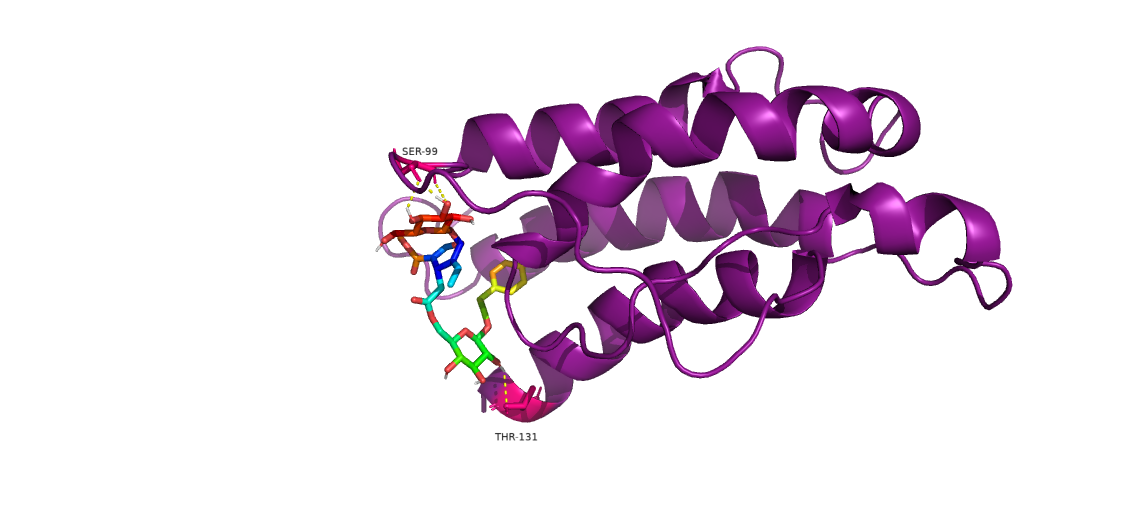


33. IL-2---ursolic acid


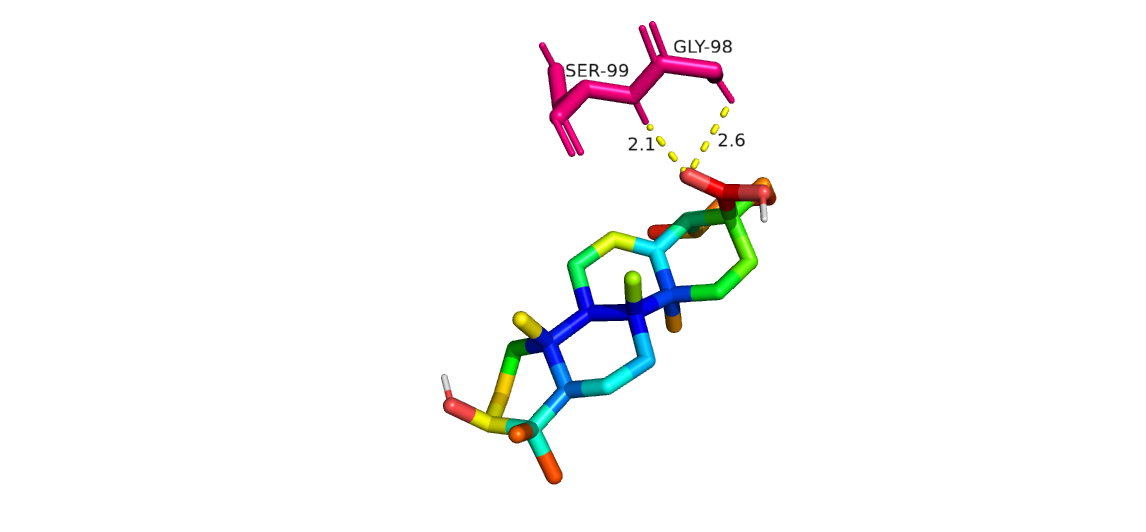

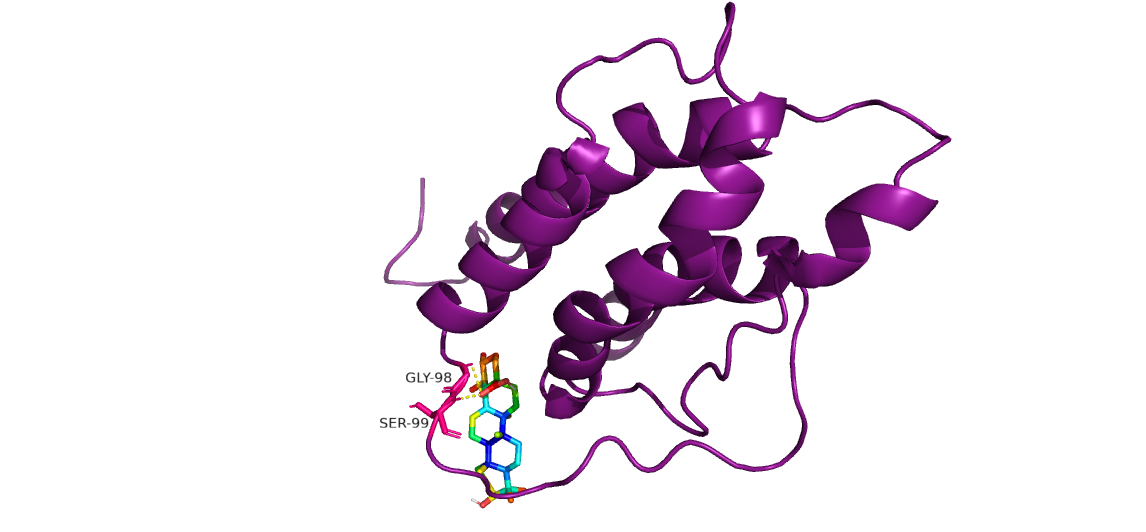


34. IL-2---wedelolactone


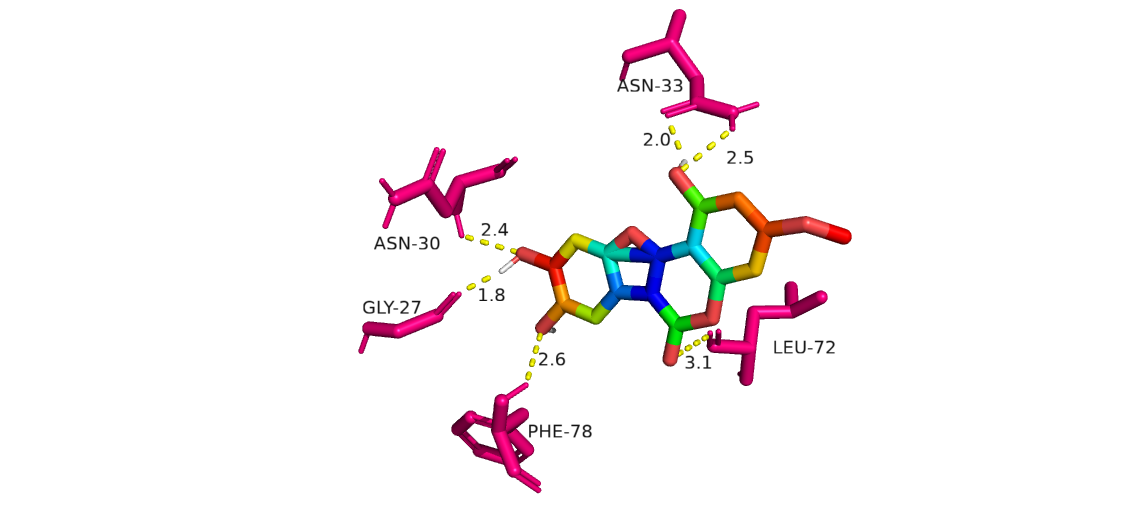

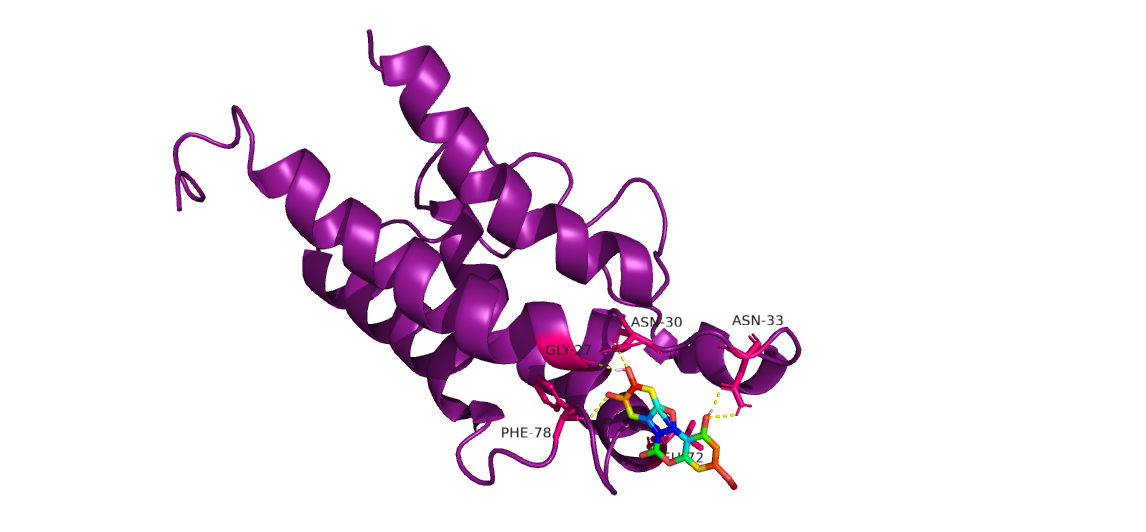


35. IL-4---3'-O-Methylorobol


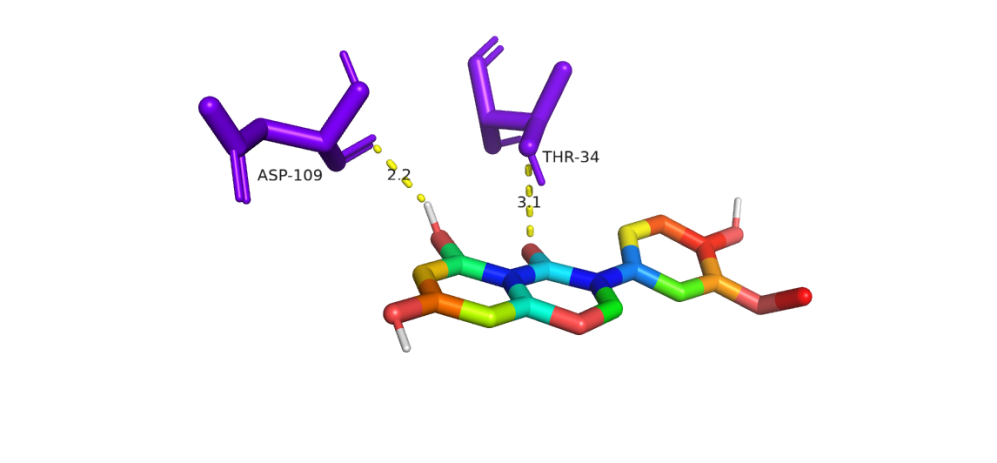

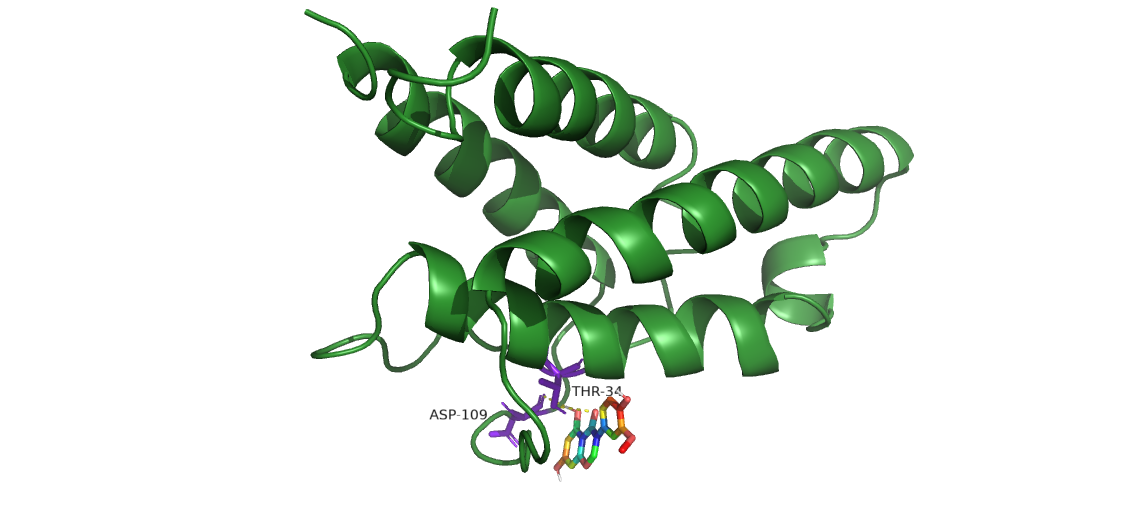


36. IL-4---acacetin


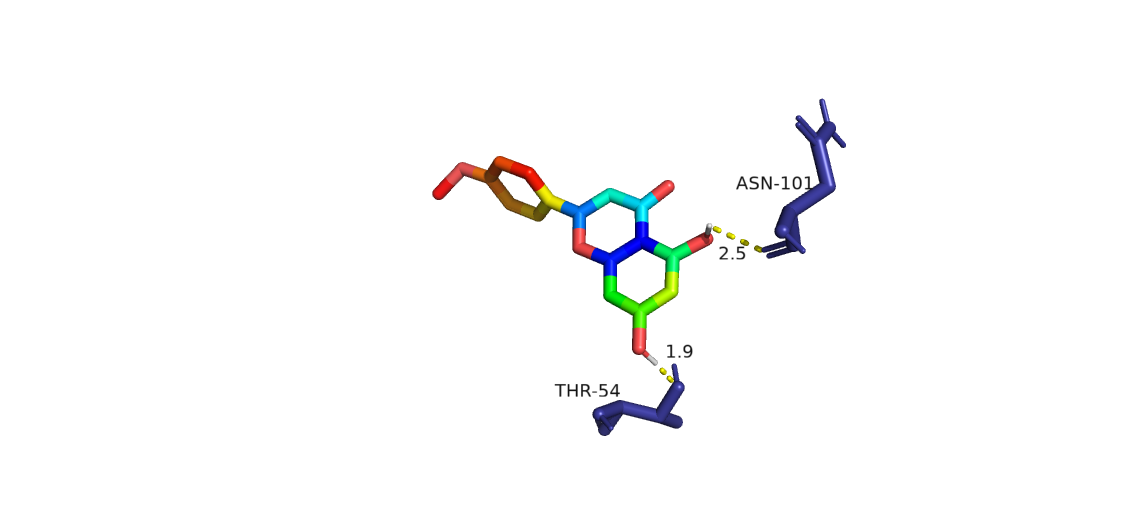

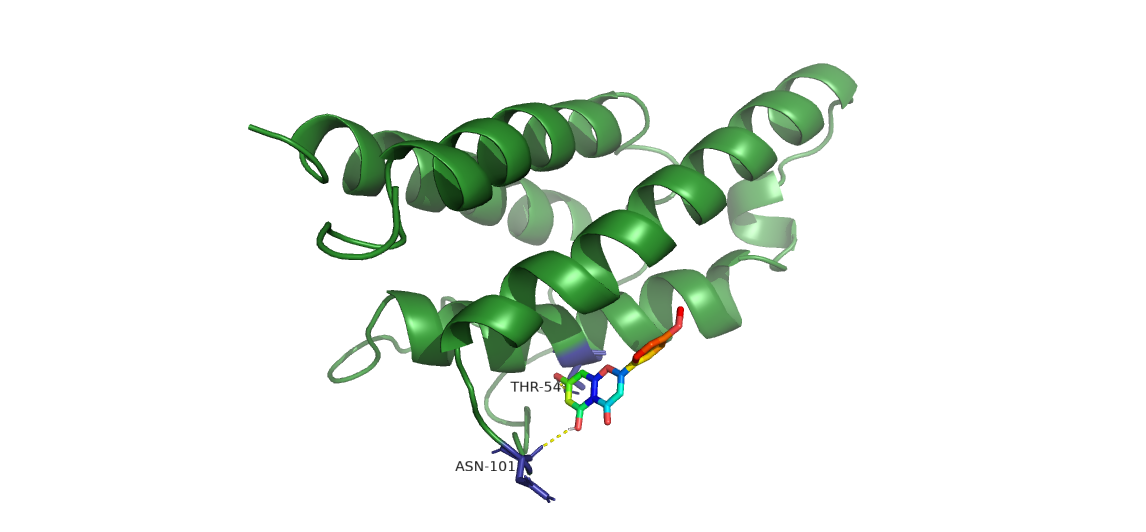


37. IL-4---apigenin


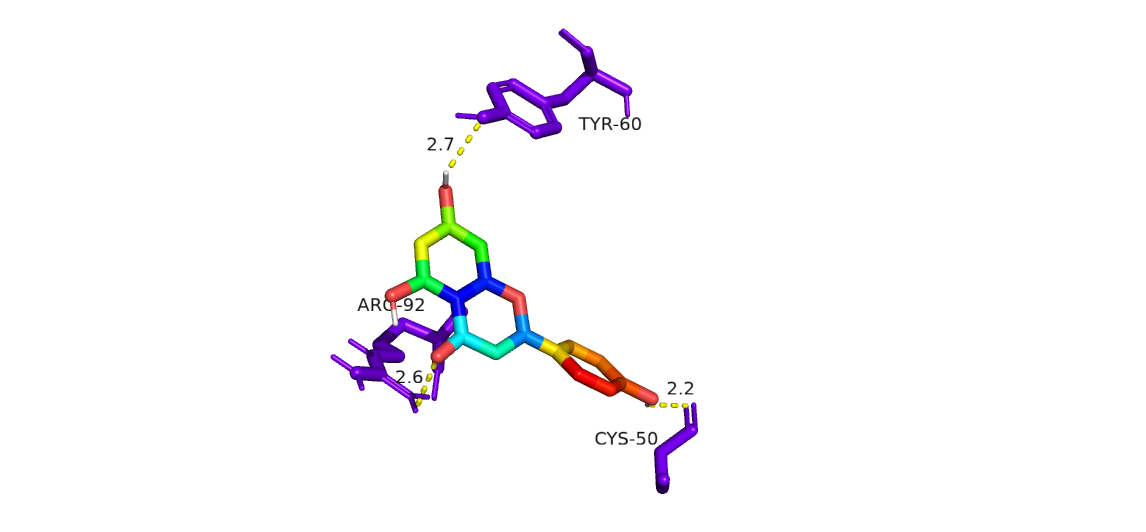

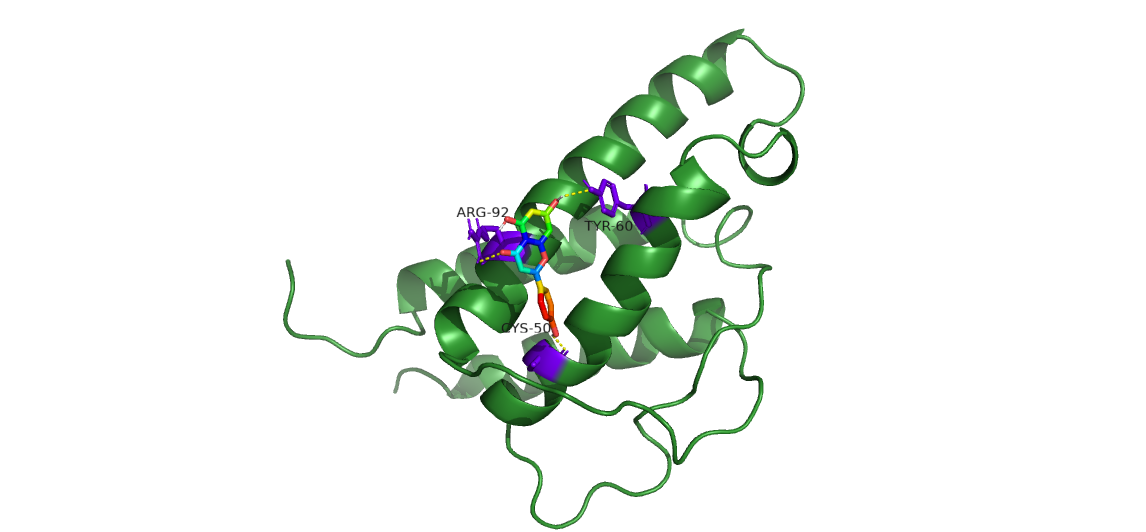


1. IL-4---beta-sitosterol

(No hydrogen bond)


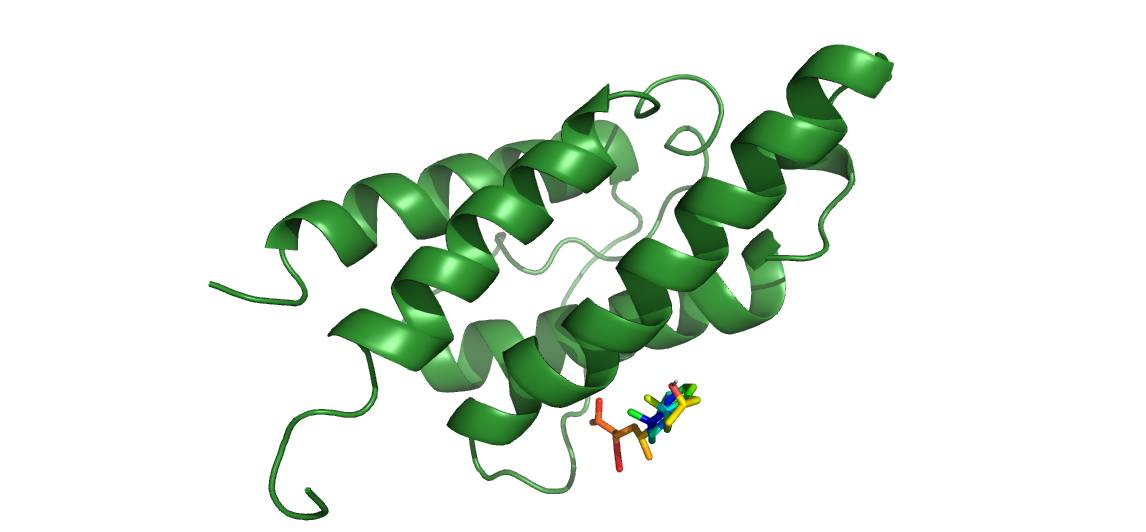


39. IL-4---daidzein


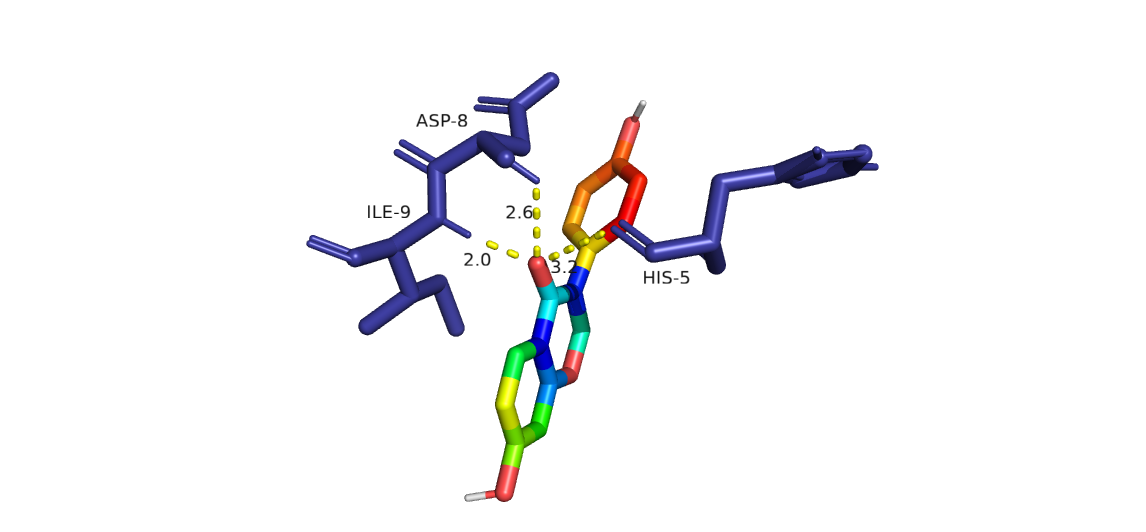

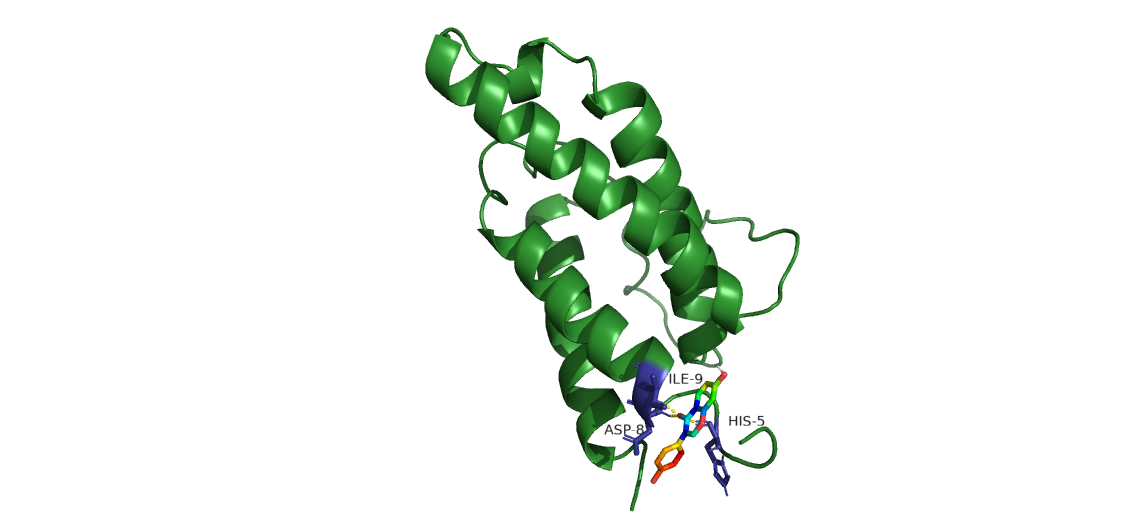


40. IL-4---DBP

(No hydrogen bond)


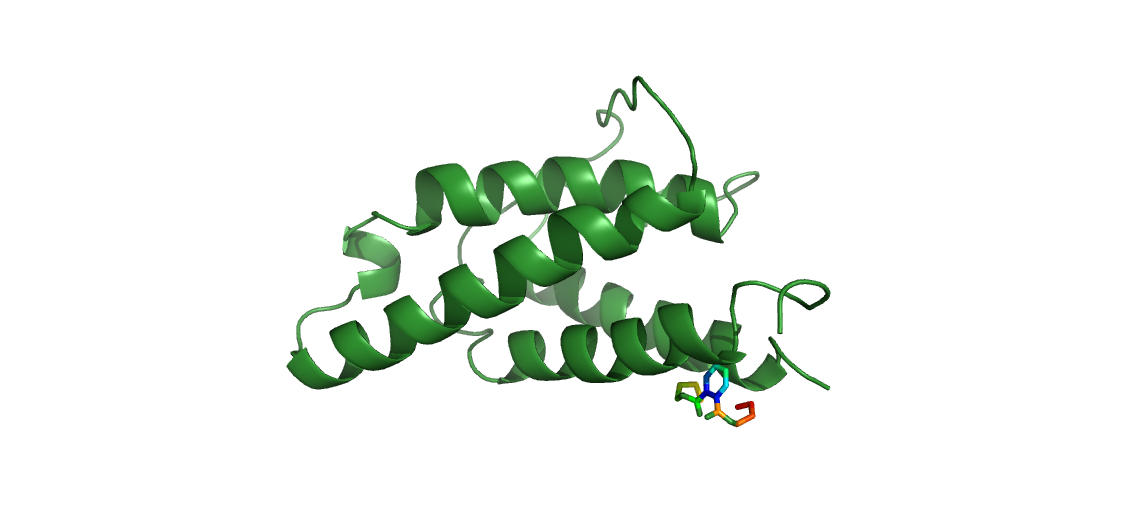


41. IL-4---demethylwedelolactone


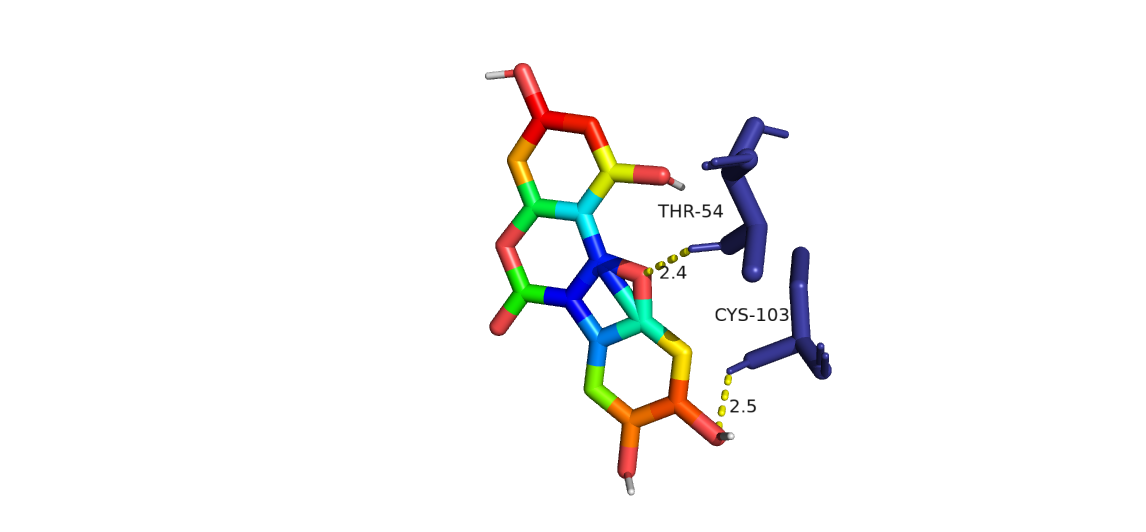

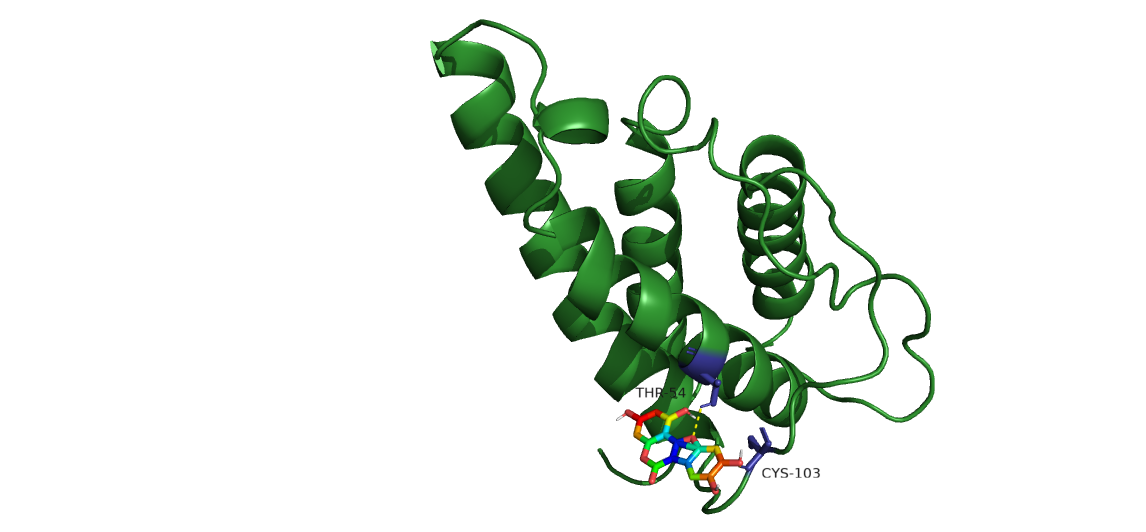


42. IL-4---kaempferol


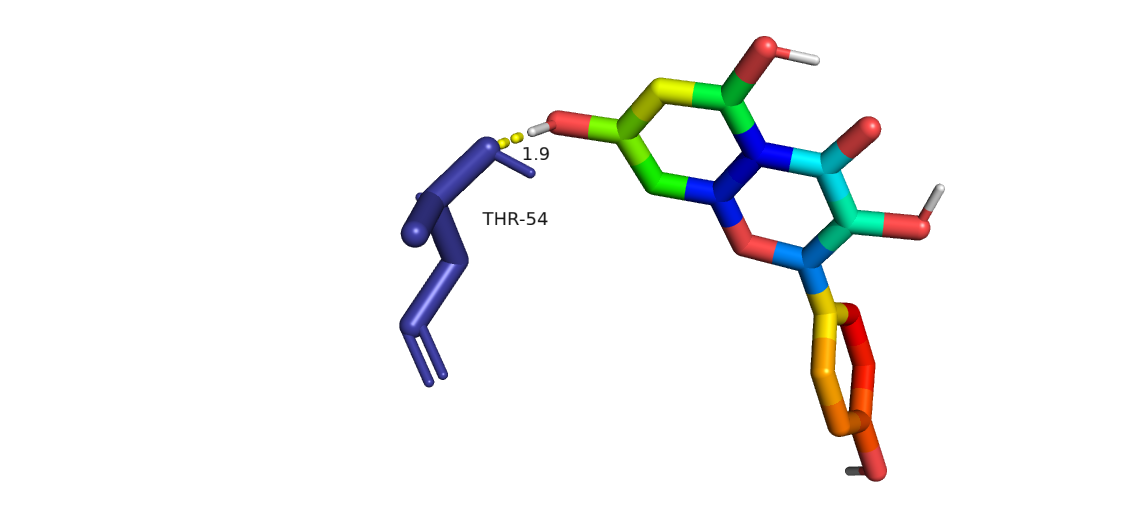

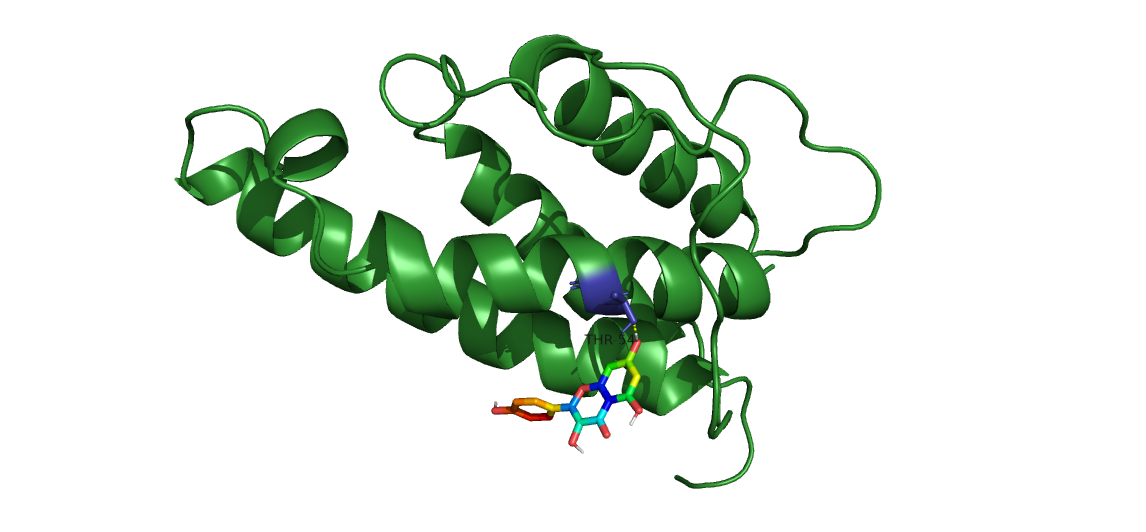


43. IL-4---LucidumosideD-qt


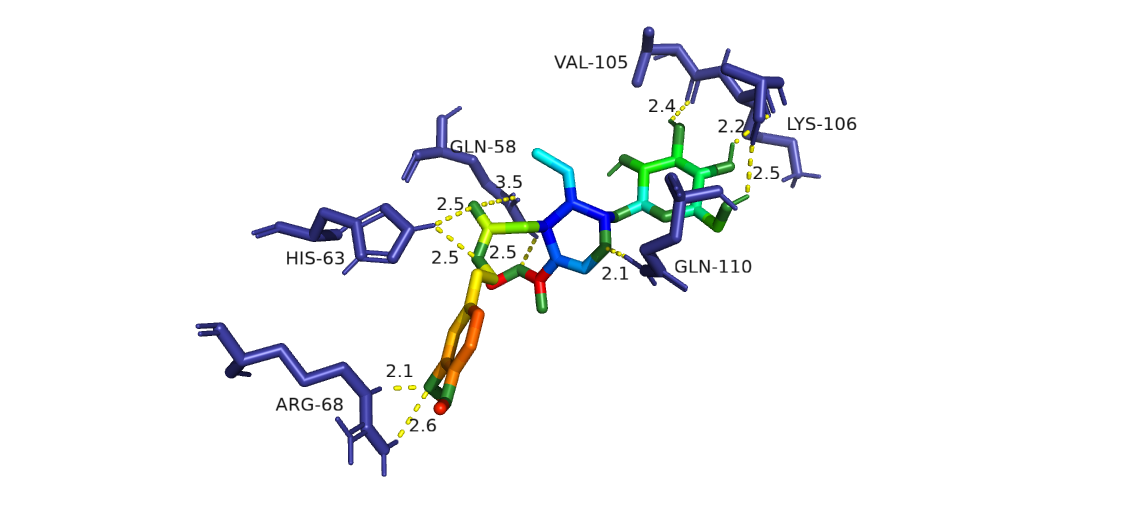

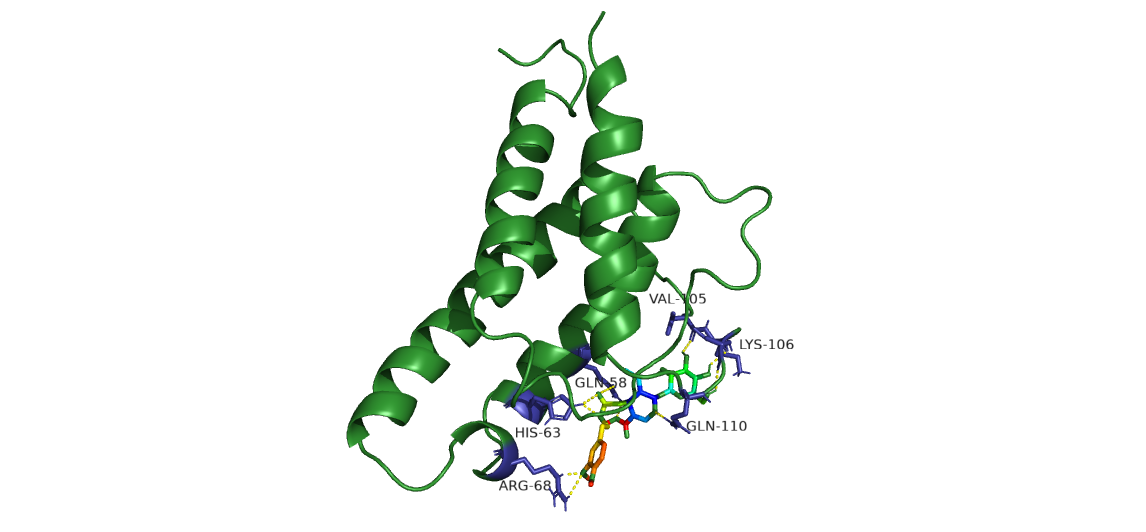


44. IL-4---luteolin


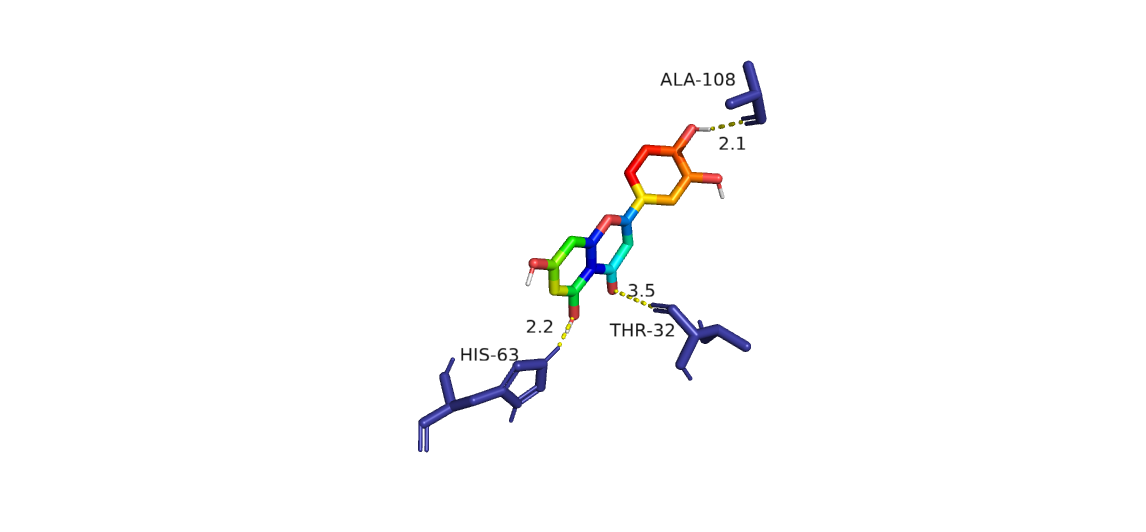

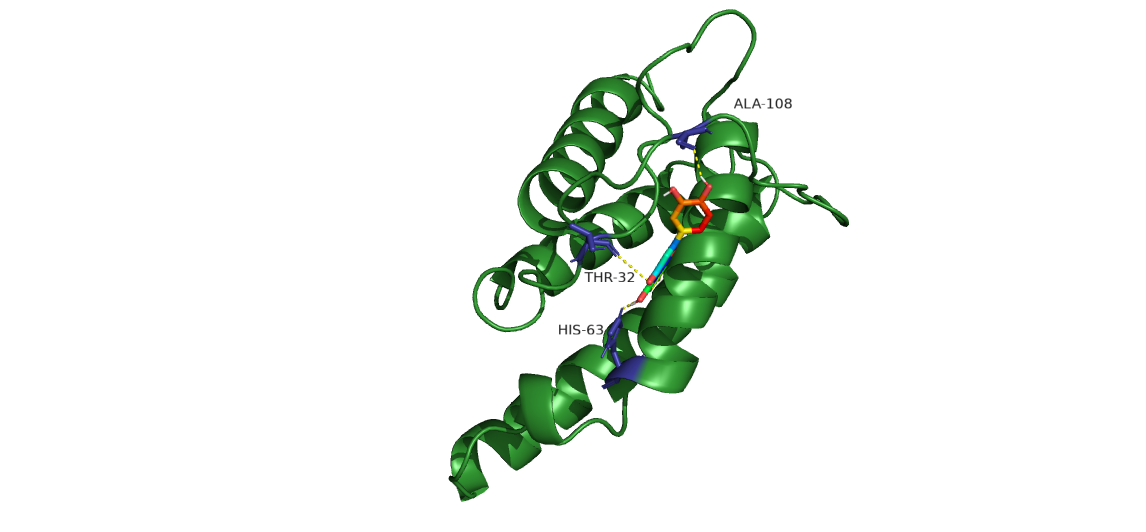


45. IL-4---Oleoside-dimethy lester-qt


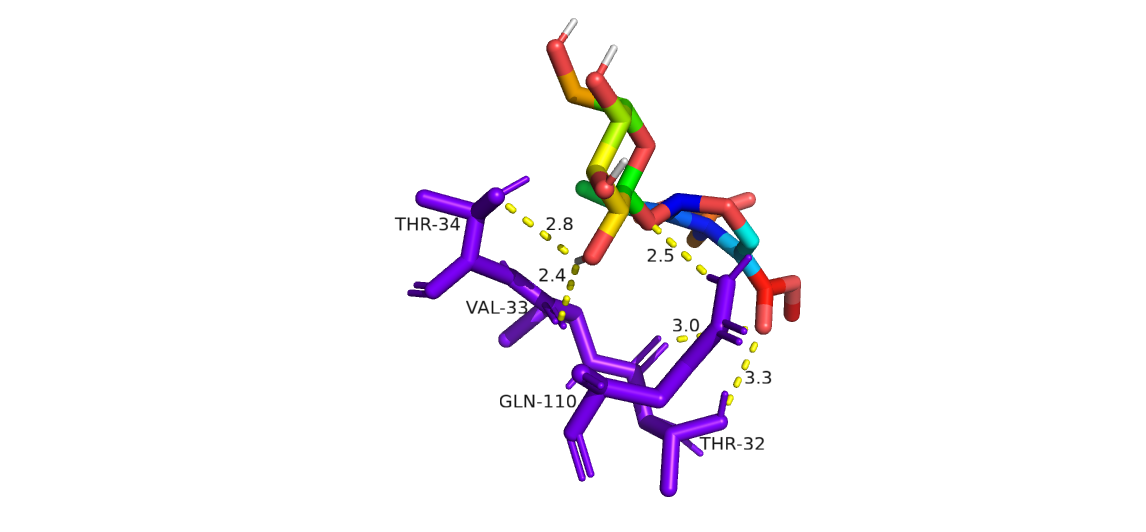

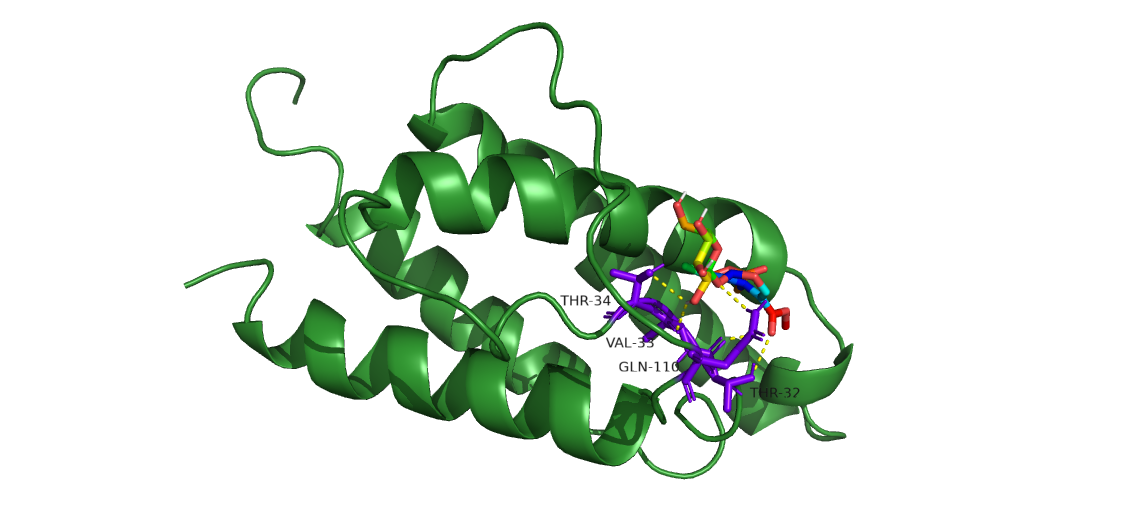


46. IL-4---Pratensein


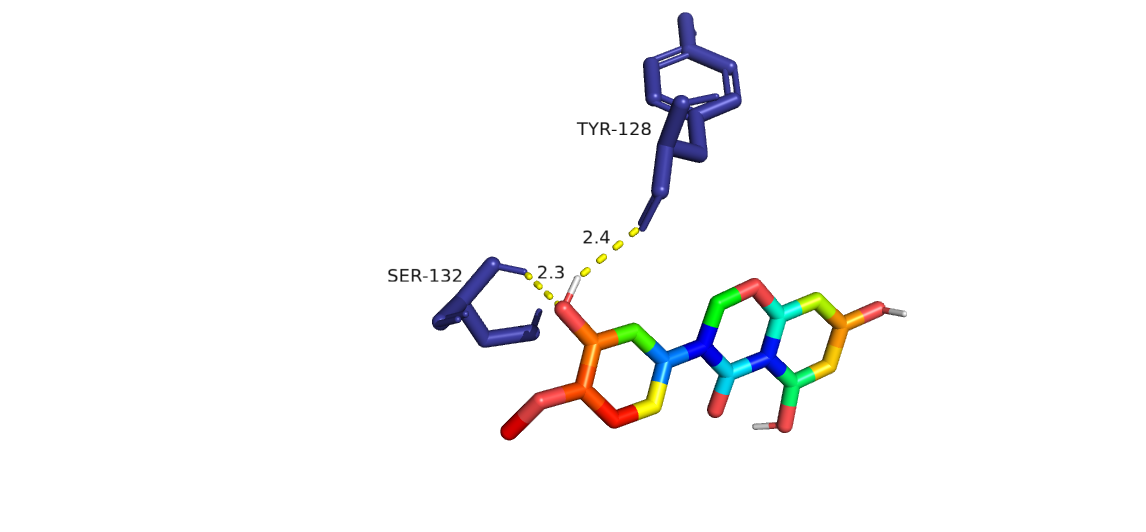

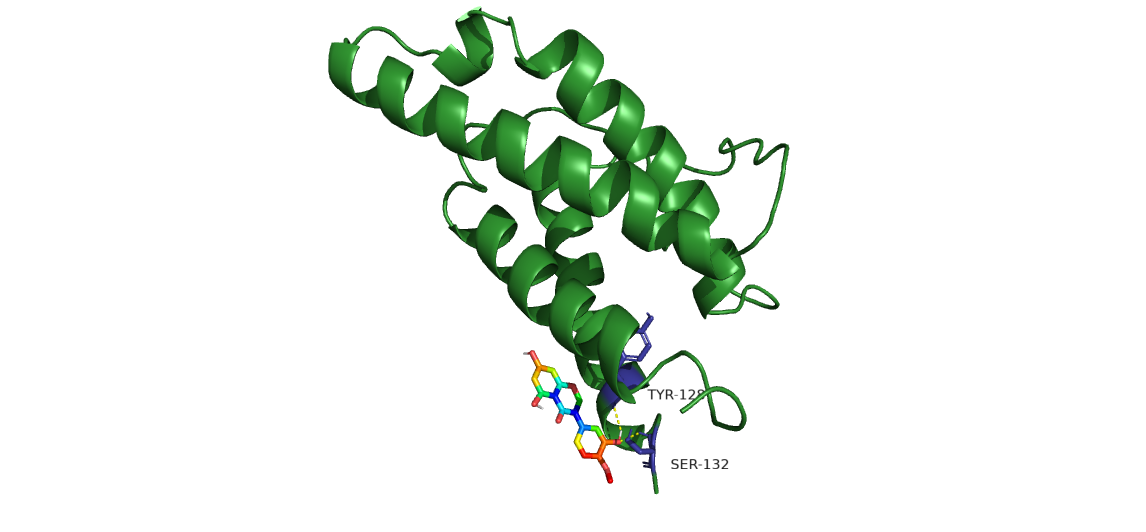


47. IL-4---quercetin


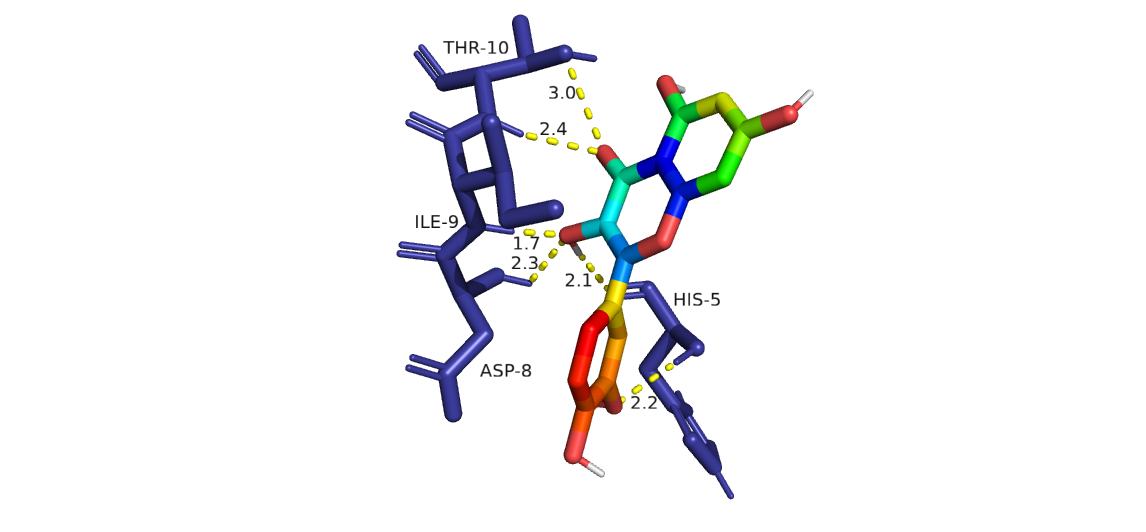

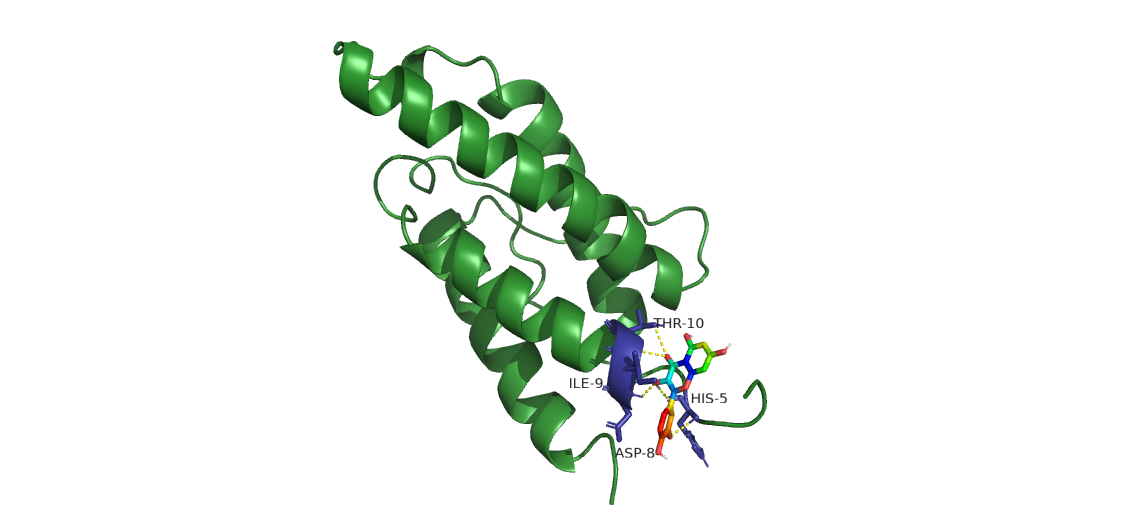


48. IL-4---salidroside


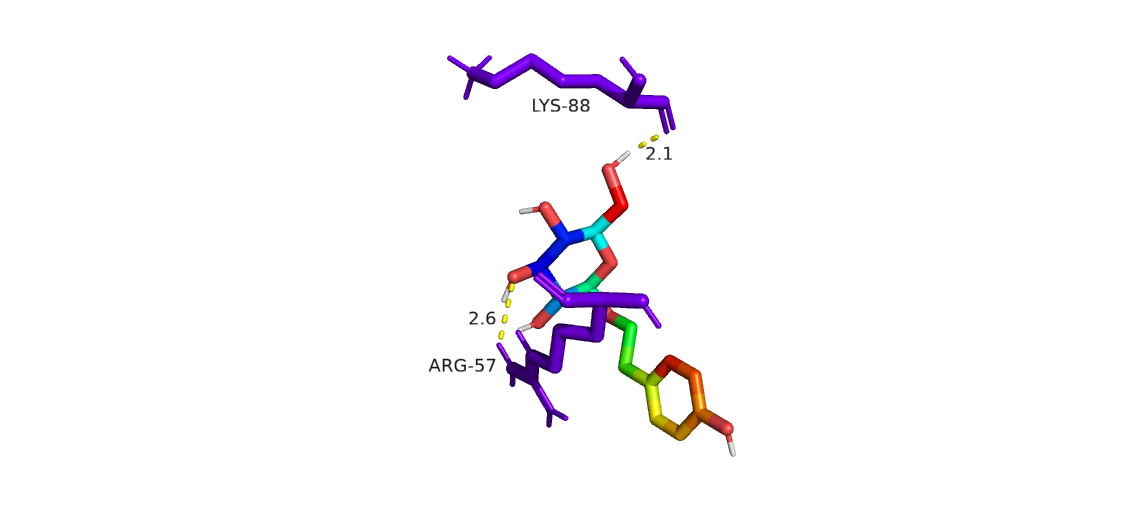

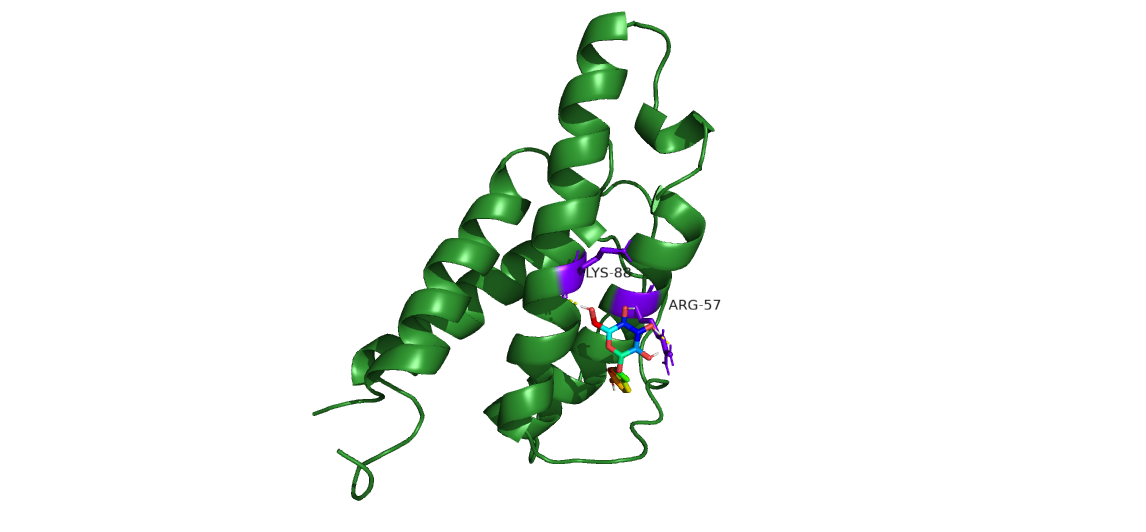


1. IL-4---Specnuezhenide


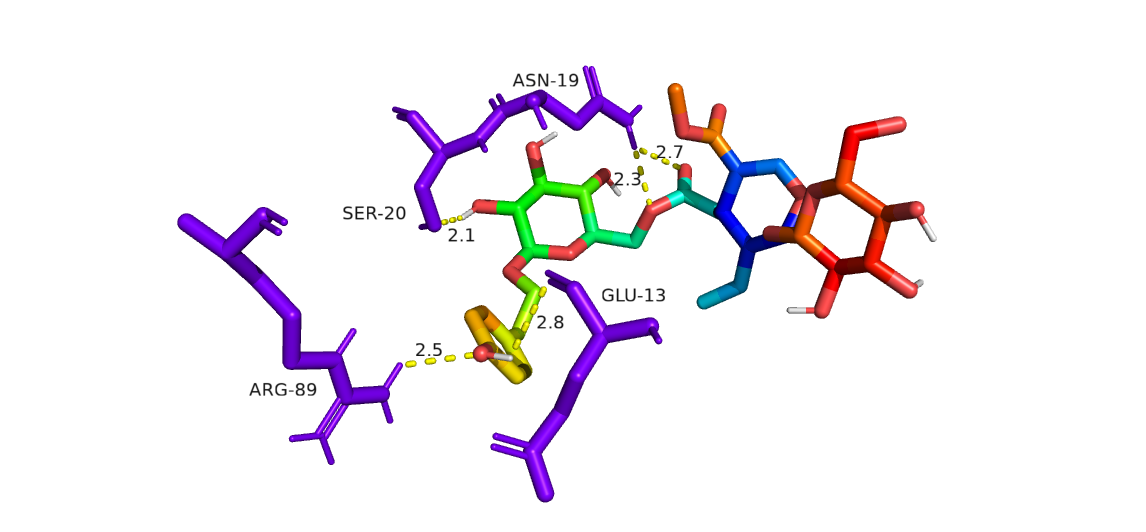

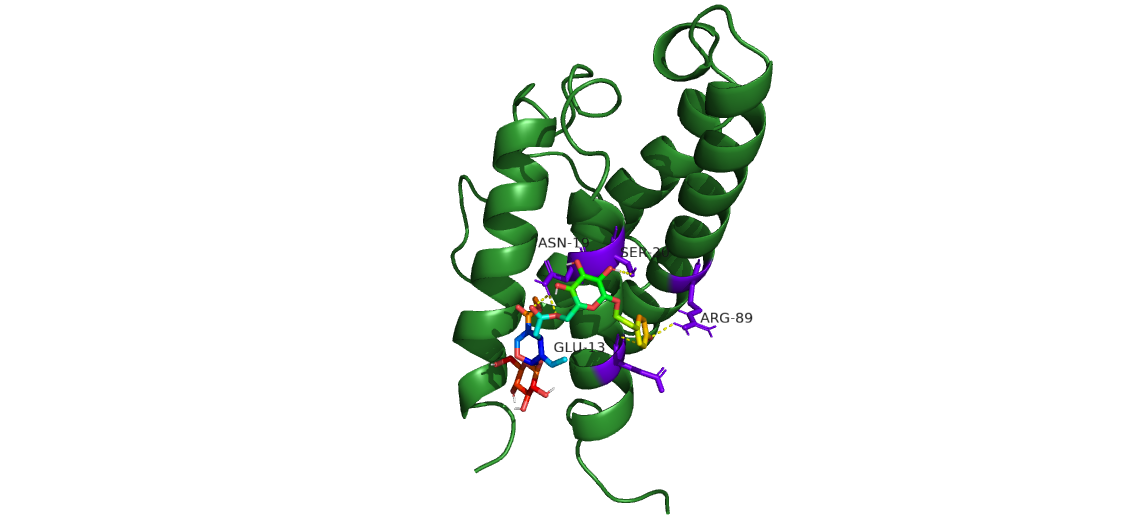


50. IL-4---ursolic-acid


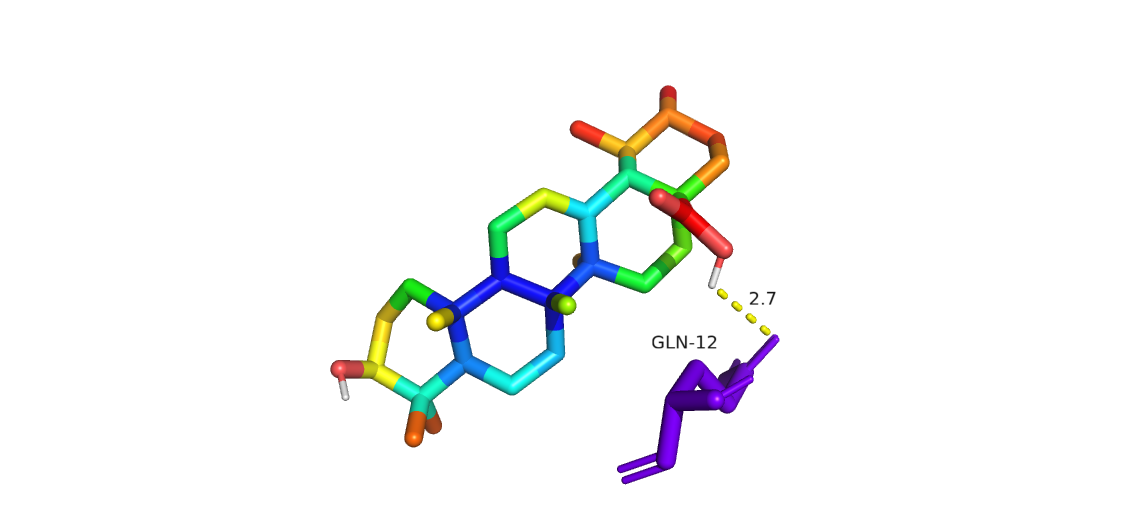

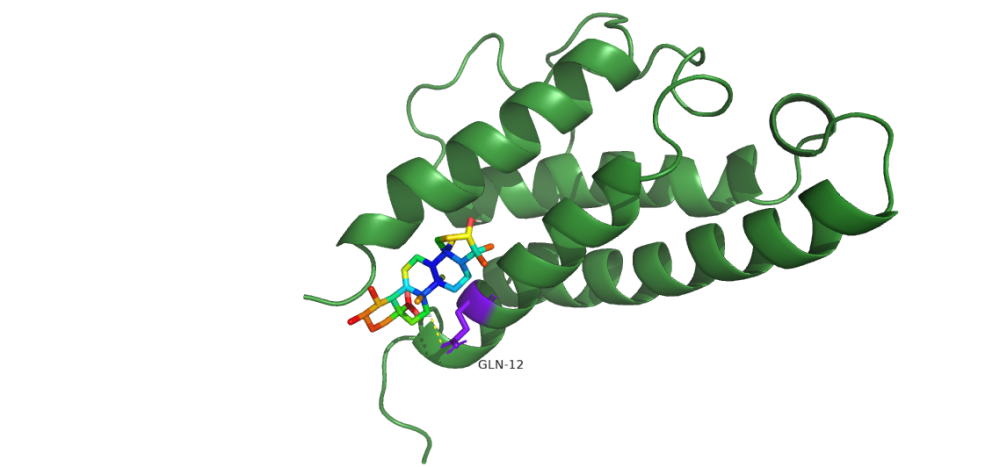


51. IL-4---wedelolactone


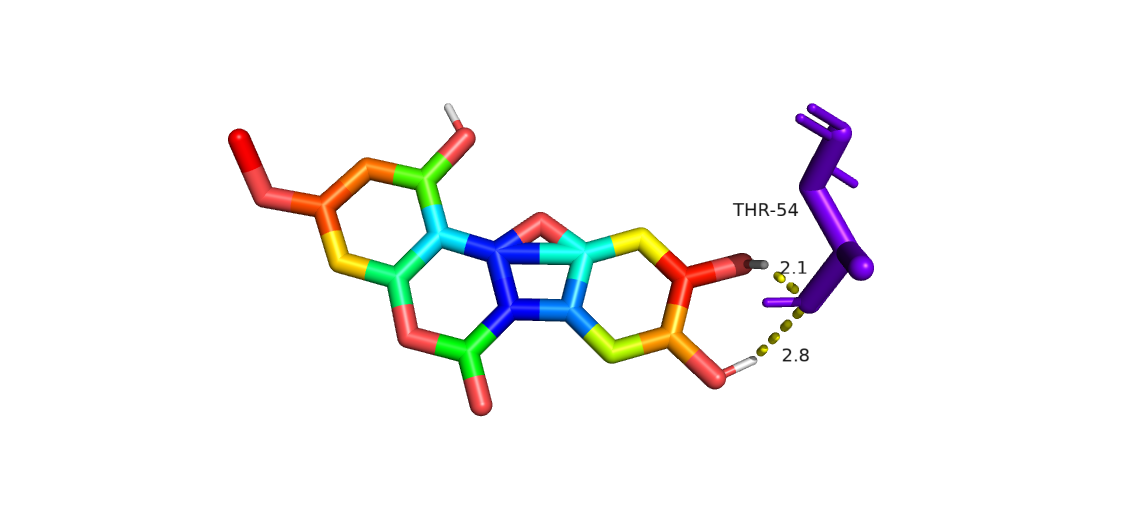

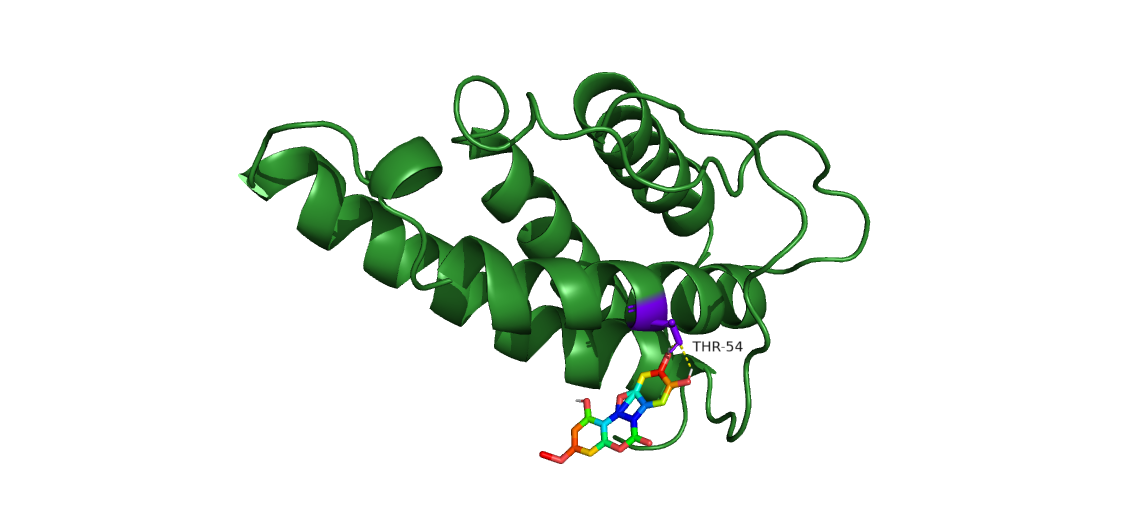

Supplement: Supplementary Materials — Supplementary File 1: 51 molecular docking results. Supplementary File 2: 51 molecular docking diagrams of EZF for treating GIOP. Supplementary File 3: determination of active components in EZF by HPLC. [file 7019792.f1.zip › 7019792.f1/Supplementary File 2 .docx]
