# Supplementary material for: Elaborate the Mechanism of Ancient Classic Prescriptions (Erzhi Formula) in Reversing GIOP by Network Pharmacology Coupled with Zebrafish Verification
Source: Evid Based Complement Alternat Med. 2022 Jan 10;2022:7019792. doi: 10.1155/2022/7019792 (PMC8763506; doi:10.1155/2022/7019792)
Supplement: Supplementary Materials — Supplementary File 1: 51 molecular docking results. Supplementary File 2: 51 molecular docking diagrams of EZF for treating GIOP. Supplementary File 3: determination of active components in EZF by HPLC. [file 7019792.f1.zip › 7019792.f1/Supplementary File 3.docx]

**Determination of active components in EZF by HPLC**

**1. Chromatographic conditions and methods**

**1.1 Preparation of reference solution**

Proper amounts of ligustroflavone, Specnuezhenide, and wedelolactone were accurately weighed, then dilute it to 10 mL with methanol to prepare the mixed reference solution. The contents of the three standard substances were all 200 μg·mL^-1^. Next, 0.05, 0.1, 0.2, 0.4, 0.6, 0.8 mL standard solution were precisely absorbed and diluted to 1 mL with methanol to prepare a series of mixed reference solution with solubility.

**1.2** **Preparation of EZF**

The lyophilized powder of the extract of EZF was accurately weighed and prepared into a solution of 1 mg·mL^-1^ with methanol in a constant volume. The solution was prepared by 0.45 μm microporous filtration membrane.

**1.3 Investigation of linear relation**

The chromatographic column was Aglient Zorbax SB-C_18_ (250 mm × 4.6 mm, 5 µm), the column temperature was 25 ° C, the detection wavelength was 227 nm, the flow rate was 1.0 mL/min, and the injection volume was 20 µL. Besides, the mobility A was acetonitrile, B was water. Gradient elution conditions were as follows: 0-2 min, 5%A; 2-40 min, 5-30% A; 40-70min, 30-80% A; 70-75 min, 80-5% A; 75-80 min, 5% A. The standard solution was determined according to the chromatographic conditions, and the peak areas of ligustroflavone, Specnuezhenide, and wedelolactone were recorded. X-axis represented concentration and y-axis represented chromatographic peak area. Finally, results were analyzed by linear regression.

**1.4 Precision test**

Under the same chromatographic conditions, standard solution was injected consecutively for 5 times.

**1.5 Repetitive test**

The freeze-dried powder of EZF was weighed according to method 1.2, so the sample solution was obtained. The contents of ligustroflavone, Specnuezhenide, and wedelolactone were measured five times in parallel, and then the RSD value was calculated.

**2. Results and Discussion**

**2.1 Chromatogram of sample solution and standard solution**

It can be detected that peaks appeared at the same time point both of the sample solution chromatogram and standard solution chromatogram. Among them, ligustroflavone, Specnuezhenide were detected when the retention time was 30 min, and wedelolactone was detected about 40 min. The results were shown as follows (Figure 1).


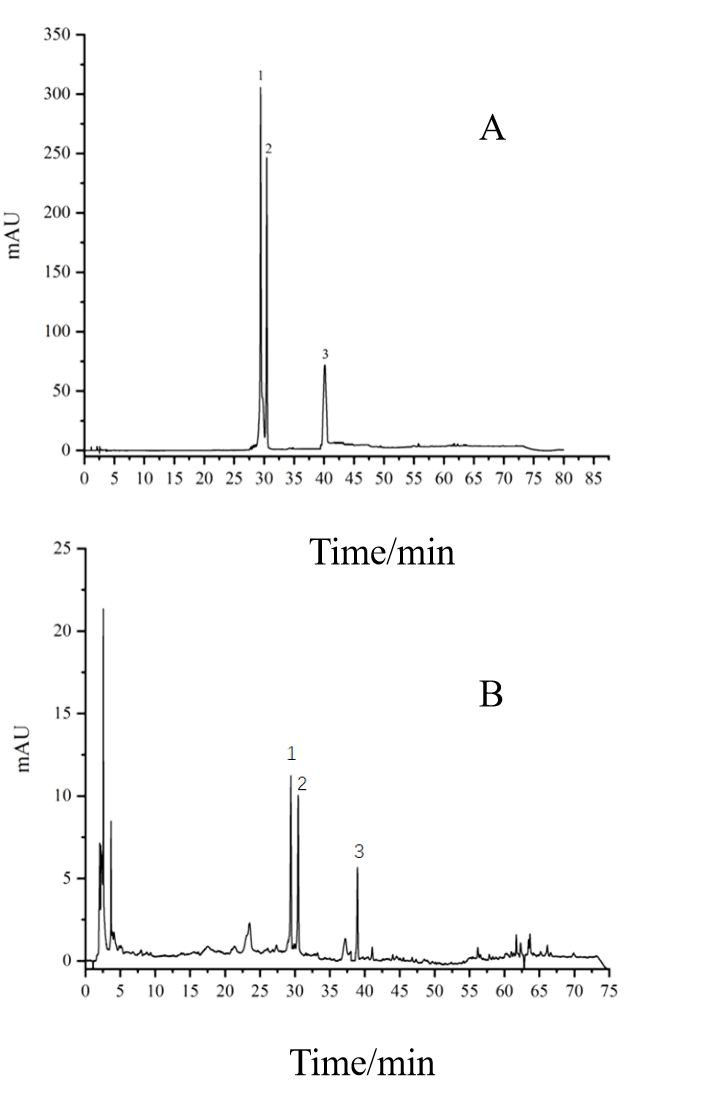


**Figure 1 The HPLC chromatogram of 3 active components in EZF**

A. Mixed standard solution; B. sample solution. 1. Ligustroflavone; 2. Specnuezhenide; 3. Wedelolactone.

**2.2 Linear relationship**

The linear equation, correlation coefficient and linear range of ligustroflavone, Specnuezhenide, and wedelolactone in EZF are determined at the same time, as shown in Table 1. It can be seen that the linear relationship of the standard curves of the three standard solution is good.

**Table. 1 linear relation of ligustroflavone, Specnuezhenide, and wedelolactone in EZF**

| **Chemical composition** | **linear equation** | **correlation coefficient** | **linear range (μg/mL)** |
| --- | --- | --- | --- |
| ligustroflavone | *Y* = 16.951*X*-98.987 | 0.9992 | 12.5~200 |
| Specnuezhenide | *Y* = 13.122*X*-105.15 | 0.9995 | 12.5~200 |
| wedelolactone | Y = 38.109X-528.74 | 0.9990 | 12.5~200 |

**2.3 Precision**

The solution was injected five times continuously, and the results were shown in Table 2. The RSD values of the three standards are less than 5%, indicating that the precision of the instrument is quite good.

**Table. 2 Peak area and precision of standard solution in EZF**

| **Number** | **ligustroflavone** | **RSD / %** | **Specnuezhenide** | **RSD / %** | **wedelolactone** | **RSD / %** |
| --- | --- | --- | --- | --- | --- | --- |
| 1 | 2660.4087 | 2.7107 | 2541.7998 | 1.8723 | 1800.4350 | 4.3634 |
| 2 | 2623.3654 |  | 2612.2645 |  | 1874.2346 |  |
| 3 | 2654.3456 |  | 2498.1234 |  | 1764.3584 |  |
| 4 | 2764.3546 |  | 2587.5412 |  | 1975.3245 |  |
| 5 | 2567.1458 |  | 2604.6974 |  | 1843.7812 |  |

**2.4 Repetitive test**

The average contents of ligustroflavone, Specnuezhenide, and wedelolactone in the freeze-dried powder of EZF extract were 2.8423 mg · g^-1^, 2.4506 mg · g^-1^ and 1.4441 mg · g^-1^ respectively, and the RSD values were 1.3216%, 1.2644% and 1.4854% respectively. They all less than 2.0%, which proved that the method had good repeatability. Detailed results are shown in Table 3.

**Table. 3 Repeatability test results of ligustroflavone, Specnuezhenide, and wedelolactone in EZF**

| **Number** | **ligustroflavone /(mg·g-1)** | **Average content** | **RSD / %** | **Specnuezhenide /(mg·g-1)** | **Average content** | **RSD / %** | **wedelolactone /（mg·g-1）** | **Average content** | **RSD / %** |
| --- | --- | --- | --- | --- | --- | --- | --- | --- | --- |
| 1 | 2.8974 | 2.8423 | 1.3216 | 2.4463 | 2.4506 | 1.2644 | 1.4365 | 1.4441 | 1.4854 |
| 2 | 2.8564 |  |  | 2.4687 |  |  | 1.4698 |  |  |
| 3 | 2.8365 |  |  | 2.4764 |  |  | 1.4123 |  |  |
| 4 | 2.8248 |  |  | 2.4631 |  |  | 1.4487 |  |  |
| 5 | 2.7968 |  |  | 2.3989 |  |  | 1.4536 |  |  |

**3. Discussion**

In this study, a method was established for the determination of three characteristic components in EZF. The method has good linearity, precision and repeatability. The chromatogram showed that the three components in the standard and the sample had good peak shapes. Ligustroflavone and Specnuezhenide are the characteristic components of FLL, while wedelolactone is the characteristic component of EP. The average contents of ligustroflavone, Specnuezhenide, and wedelolactone in EZF were 2.8423 mg·g-^1^, 2.4506 mg·g^-1^ and 1.4441 mg·g^-1^ respectively.
